# Canalization of genome-wide transcriptional activity in *Arabidopsis thaliana* accessions by MET1-dependent CG methylation

**DOI:** 10.1186/s13059-022-02833-5

**Published:** 2022-12-20

**Authors:** Thanvi Srikant, Wei Yuan, Kenneth Wayne Berendzen, Adrián Contreras-Garrido, Hajk-Georg Drost, Rebecca Schwab, Detlef Weigel

**Affiliations:** 1 Department of Molecular Biology, Max Planck Institute for Biology Tübingen, Tübingen, Germany; 2 Present address: Institute of Molecular Plant Biology, Department of Biology, ETH Zürich, Zürich, Switzerland; 3 Plant Transformation and Flow Cytometry Facility, ZMBP, University of Tübingen, Tübingen, Germany; 4 Computational Biology Group, Max Planck Institute for Biology Tübingen, Tübingen, Germany

**Keywords:** Natural variation, Epigenetics, DNA methylation, Methyltransferase, *Arabidopsis*

## Abstract

**Background:**

Despite its conserved role on gene expression and transposable element (TE) silencing, genome-wide CG methylation differs substantially between wild *Arabidopsis thaliana* accessions.

**Results:**

To test our hypothesis that global reduction of CG methylation would reduce epigenomic, transcriptomic, and phenotypic diversity in *A. thaliana* accessions, we knock out *MET1*, which is required for CG methylation, in 18 early-flowering accessions. Homozygous *met1* mutants in all accessions suffer from common developmental defects such as dwarfism and delayed flowering, in addition to accession-specific abnormalities in rosette leaf architecture, silique morphology, and fertility. Integrated analysis of genome-wide methylation, chromatin accessibility, and transcriptomes confirms that *MET1* inactivation greatly reduces CG methylation and alters chromatin accessibility at thousands of loci. While the effects on TE activation are similarly drastic in all accessions, the quantitative effects on non-TE genes vary greatly. The global expression profiles of accessions become considerably more divergent from each other after genome-wide removal of CG methylation, although a few genes with diverse expression profiles across wild-type accessions tend to become more similar in mutants. Most differentially expressed genes do not exhibit altered chromatin accessibility or CG methylation in *cis*, suggesting that absence of MET1 can have profound indirect effects on gene expression and that these effects vary substantially between accessions.

**Conclusions:**

Systematic analysis of MET1 requirement in different *A. thaliana* accessions reveals a dual role for CG methylation: for many genes, CG methylation appears to canalize expression levels, with methylation masking regulatory divergence. However, for a smaller subset of genes, CG methylation increases expression diversity beyond genetically encoded differences.

**Supplementary Information:**

The online version contains supplementary material available at 10.1186/s13059-022-02833-5.

## Background

Eukaryotic gene expression can be fine-tuned by epigenetic changes such as modifications to DNA, histones, and changes in chromatin architecture. DNA methylation is established and maintained by a cohort of methyltransferases, including MET1/DNMT1 (DNA METHYLTRANSFERASE 1), which semi-conservatively copies methylation marks in the CG nucleotide context from the template to the daughter strand. In plants, MET1 is the principal enzyme for establishing cytosine methylation in replicating cells, especially during fertilization and embryogenesis [1, 2].

In *A. thaliana*, even partial inactivation of *MET1* can profoundly alter the genome-wide distribution of cytosine methylation, often causing phenotypic abnormalities due to the emergence of epialleles affecting the activity of developmental genes [3, 4]. These effects are aggravated when *MET1* activity is reduced further, as in the EMS-induced *met1-*1 mutant and the T-DNA insertion mutant *met1-*3, in which genome-wide CG methylation is largely eliminated, particularly at pericentromeric heterochromatin [5, 6]. This in turn affects histone methylation [7–9], chromatin accessibility, and long-range chromatin interactions [10] and also leads to ectopic methylation by de novo cytosine methylation pathways [11, 12].

The genomes of natural accessions of *A. thaliana* vary considerably, with an average of one single nucleotide polymorphism (SNP) every 200 base pairs of the genome in a given pairwise comparison of accessions from different parts of the geographical range [13]. Natural accessions also vary substantially in their methylome, transcriptome, and mobilome (transposable element, TE) landscapes [14–17]. Large-scale structural variation along with methylome variation at TEs is influenced by genetic variation at loci encoding components of the methylation machinery, suggesting that the methylation machinery is a target of selection during adaptation to the environment [15, 16, 18–21]. Substantial variation in methylation is also apparent in genic regions, functioning as a storehouse of epialleles, some of which can impact key developmental processes and fitness under new environments [22, 23]. Despite the documented variation in methylome patterns and the known connections between DNA methylation and gene expression, how much variation in DNA methylation contributes to adaptive variation in gene transcriptional activity remains a matter of intense debate [24–27].

Given the large variation in methylomes across *A. thaliana* accessions, we hypothesized that reduction of methylation would reduce differences in chromatin accessibility and gene expression between accessions. To study genetic-background-dependent responses to genome-wide CG hypomethylation, we generated *met1* loss-of-function mutants in 18 *A. thaliana* accessions. The number of differentially expressed genes varied greatly in accessions. While TE-related genes behaved very similarly across accessions, responding nearly uniformly with a substantial increase in expression, non-TE-associated genes were much more variable, both in terms of the number of differentially expressed genes and the extent of expression change of individual genes. However, a small group of genes with divergent expression profiles across wildtypes became more similar in expression once MET1 was lost. We conclude that MET1-dependent DNA methylation has dual roles, reducing differences in the transcriptional activity of diverse genomes for most genes, but increasing transcriptional diversity of a minority of genes.

## Results

### Generation of *met1* mutants

We used CRISPR-Cas9 mutagenesis [28] to target the *MET1* gene (*AT5G49160*) in 18 early-flowering accessions of *A. thaliana*, creating frameshift mutations in exon 7 (“Methods,” Additional file 1: Table S1). For each accession, transgene-free lines were genotyped at the *MET1* locus and propagated. In the next generation, we obtained homozygous *met1* mutants from 17 accessions, with two mutant lines of independent origin for 14 accessions and one mutant line for Cvi-0, Ler-1, and Col-0. For Bl-1, we did not recover a sufficient number of homozygous progeny for in-depth analysis. Because Bl-1 heterozygotes already had morphological defects, we included these in our analyses, along with heterozygous mutants in Col-0, Ler-1, and Bu-0. We also included second-generation *met1* homozygotes from Tsu-0 and Tscha-1 (descended from siblings of first-generation homozygotes) to glean first insights into progressive changes at later generations of homozygosity. For one Bs-1 line with bi-allelic mutations in *MET1,* we only analyzed second-generation progeny that was homozygous for one of the two alleles.

Previous work showed that epigenetic states across the genome can diverge in different lineages of *met1* mutants over several generations [5, 11]. To ensure that we could directly link chromatin state and gene expression, we performed paired BS-seq, ATAC-seq, and RNA-seq on leaf tissue of the same plant rosettes, collected as three biological replicates for both wild-type and *met1* mutant lines (“Methods”). All together, we obtained 73 BS-seq, 158 ATAC-seq, and 158 RNA-seq libraries that passed quality control.

### MET1 can both buffer and increase transcriptomic variation across accessions

**Fig. 1.**
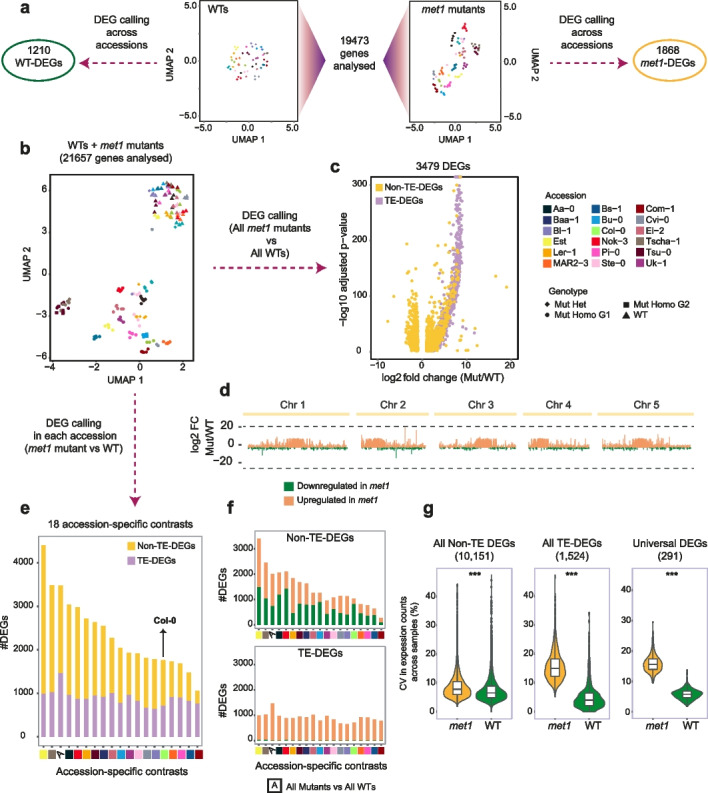
Transcriptomic variation among accessions in *met1* mutants and wildtypes. **a** UMAP projections of transformed RNA-seq read counts in 19,473 genes similarly compared for wildtypes (left) and *met1* mutants (right). These genes were further analyzed to identify DEGs across accessions, separately for WTs and *met1* mutants. **b** UMAP representation of transformed RNA-seq counts from 158 samples (104 hetero- or homozygous *met1* mutants and 54 wild-type plants) across 21,657 genes. Colors indicate accessions, and shapes indicate genotype. WT, wild-type; Mut Het, heterozygous *met1* mutants; Mut Homo G1, first-generation homozygous *met1* mutants; Mut Homo G2, second-generation homozygous *met1* mutants. **c** Volcano plot of 3479 DEGs identified in a contrast between all *met1* mutant samples and all wild-type samples. TE-associated DEGs (TE-DEGs) are colored purple, and Non-TE-DEGs yellow. **d** Chromosomal distribution of 3479 DEGs from the all-*met1*-against-all-wild-type contrast, and their log_2_(fold change) in mutants relative to the corresponding wildtypes. Upregulated DEGs are colored orange and downregulated DEGs green. **e** DEGs in the 18 accession-specific contrasts, compared to the all-*met1*-against-all-wild-type contrast (denoted by “A,” third column from the left). **f** Variation in numbers of upregulated and downregulated Non-TE-DEGs and TE-DEGs across different contrasts, bars colored similarly to **d**. For **e** and **f**, colors below bars indicate accession-specific contrasts. **g** Boxplots showing distribution of the coefficient of variation (CV) for expression level (measured in transformed read counts) across accessions, compared between 104 *met1* mutant and 54 wild-type samples at 10,151 Non-TE-DEGs, 1,524 TE-DEGs, and 291 Universal DEGs. *** indicates Wilcoxon-test *p*-value <0.0001

Since natural accessions of *A. thaliana* are known to express diverse transcriptomes in their wild-type state [16], we hypothesized that MET1-induced CG methylation may contribute to generating this diversity and that the transcriptomes would become more similar to each other in *met1* mutants. We therefore first grouped all wildtypes (54 samples) and all *met1* mutants (104 samples) separately, and analyzed 19,473 genes with sufficient read counts in both groups. Contrary to our hypothesis, UMAP projections of read counts at these genes revealed greater differences between accessions in *met1* mutants compared to their respective wild-type samples (Fig. 1a). This suggested an alternate hypothesis where MET1 functions in buffering transcriptomic diversity across accessions, with its absence therefore unmasking larger regulatory differences. Wild-type samples had 1210 differentially expressed genes (DEGs; defined as genes with |log_2(_fold change)|≥1, FDR adjusted *p*-value≤0.01) (Additional file 2: Table S2, Additional file 3: Dataset S1) between accessions, fewer than those identified between accessions in *met1* mutant samples (1868 DEGs) (Additional file 2: Table S2, Additional file 4: Dataset S2). There were only 406 DEGs that were shared by wildtypes and *met1*, i.e., which were differentially expressed independently of *MET1* activity. These are likely to include genes that are associated with structural variation or major differences in cis-regulatory sequences. There were 804 DEGs that were unique to wild-type samples, i.e., where differences in expression between accessions were greatly reduced upon loss of *MET1*. Finally, the largest group in this comparison comprised 1462 DEGs that were unique to *met1* samples, i.e., which were expressed at similar levels across wildtypes, but became differentially expressed in *met1* mutants. These inferences were also apparent from heatmaps (Additional file 5: Fig. S1) and density distributions (Additional file 5: Fig. S2) of Pearson correlation coefficients between accessions. Together, these observations indicate that there is evidence for both of our alternative hypotheses—MET1 reduces transcriptomic diversity across accessions for many genes, but for a smaller group of genes it adds another layer of expression complexity to diversify transcriptomes.

### TE- and Non-TE genes are differentially expressed in *met1* mutants

A major role of *MET1* is the transcriptional silencing of TEs by establishing and maintaining CG methylation [29, 30]. We next asked whether there were any accession-specific differences in the requirement of *MET1* in regulating the expression of both TE genes (genes associated with TEs, see “Methods”) and other protein-coding genes. We first analyzed RNA-seq read counts of 21,657 genes in all samples (wildtypes and *met1* mutants) and generated a UMAP visualization. Two distinct clusters of samples could be observed, one encompassing wild-type and the other *met1* plants (Fig. 1b). In agreement with our initial results, wild-type samples were less spread out than mutant samples, indicating greater gene expression heterogeneity in *met1* mutants than in wild-type plants. Comparison of first- and second-generation homozygous mutants in the Tscha-1 and Tsu-0 accessions did not indicate major changes upon propagation of mutant lines.

To obtain first insights into MET1 function across all accessions, we examined protein-coding genes whose expression levels changed significantly in a contrast of all *met1* mutants against all wild-type plants, thereby identifying 3479 DEGs. Of these, 1466 genes (42% of all DEGs) were associated with TEs, which we called TE-DEGs (Additional file 2: Table S2, Additional file 6: Dataset S3), and these corresponded to 87% of the 1678 TE-associated genes with sufficient information in our dataset (“Methods”). Almost all of them were upregulated in *met1* mutants compared to wild-type plants, and often greatly so (Fig. 1c). This was consistent with previous findings from early- and late-generation homozygous *met1* mutants [31–35], including the observation that Class II DNA TEs of the En/Spm superfamily were most often among activated TE-DEGs [36, 37]. Consistent with TE density being highest near the centromeres [38], TE-DEGs were enriched in pericentromeric regions (Fig. 1d). Many of the TE-DEGs were strongly overexpressed in *met1* mutants, hundreds of times or more (Fig. 1c).

The picture was different for the remaining 2013 DEGs that were not associated with TEs (Non-TE-DEGs, Additional file 2: Table S2, Additional file 6: Dataset S3), and which corresponded to 10% of the 19,979 Non-TE genes with sufficient information in our dataset. Although the majority was also upregulated in *met1* mutants, more than a third, 728, was downregulated (Fig. 1c). Non-TE-DEGs were also more uniformly distributed along the chromosome arms, and overall expression changes for both up- and downregulated genes were much more moderate (Fig. 1d). Gene Ontology (GO) enrichment of Non-TE-DEGs revealed several terms related to abiotic and biotic stresses and stimulus response (Additional file 5: Fig. S3). We conclude that across all accessions, Non-TE-associated genes vary much more in their sensitivity to loss of MET1 than TE-associated genes.

### Mis-regulated genes vary among *met1* mutants of different accessions

A closer examination of DEGs called by contrasting all *met1* mutants against all wildtypes showed that DEGs were not uniformly induced or repressed across accessions (a random subset of such DEGs is shown in Additional file 5: Fig. S4). Hence we individually examined each of the 18 accessions for genes that were differentially expressed between *met1* mutants and the corresponding wild-type parents. We found that the number of DEGs in each accession varied substantially (Fig. 1e, Additional file 2: Table S2, Additional file 6: Dataset S3), but that this was much more true for Non-TE-DEGs than TE-DEGs. In addition, while downregulated TE-DEGs were rare in all accessions, the ratio of up- to downregulated Non-TE-DEGs in *met1* mutants was much more variable (Fig. 1f, Additional file 5: Fig. S5). The number of Non-TE-DEGs ranged from 278 (26% of all DEGs) in Com-1 to 3409 (77% of all DEGs) in Est. Notably, even though we could only analyze heterozygous Bl-1 *met1* mutants, these had more Non-TE-DEGs than homozygous *met1* mutants in several other accessions, suggesting that even the removal of only one of the two functional *MET1* copies was sufficient to alter expression levels of a large number of genes, at least in this accession.

In total, there were 10,151 Non-TE-DEGs and 1524 TE-DEGs that were differentially expressed in at least one of the accessions or in the all-*met1*-against-all-wild-type contrast. Expression levels of both DEG sets were significantly more variable across accessions in *met1* mutants compared to wildtypes (Fig. 1g, Additional file 5: Fig. S6), once again demonstrating that expression diversity across accessions was overall higher in the absence of *MET1* activity.

We used all 10,151 Non-TE-DEGs to build a weighted gene co-expression network (“Methods”), finding nine modules. Genes from module “D” were the most consistent in their expression levels across all accessions, being on average always upregulated in *met1* mutants compared to the respective wild-type plants (Additional file 5: Fig. S7, Additional file 7: Dataset S4). GO enrichment analyses revealed that many “D module” genes were associated with nucleic acid metabolic processes, DNA repair and transcription, although only weakly significantly so. This suggests that MET1 likely affects core metabolic machinery genes, which may further impact a different subset of downstream genes in each accession.

**Fig. 2:**
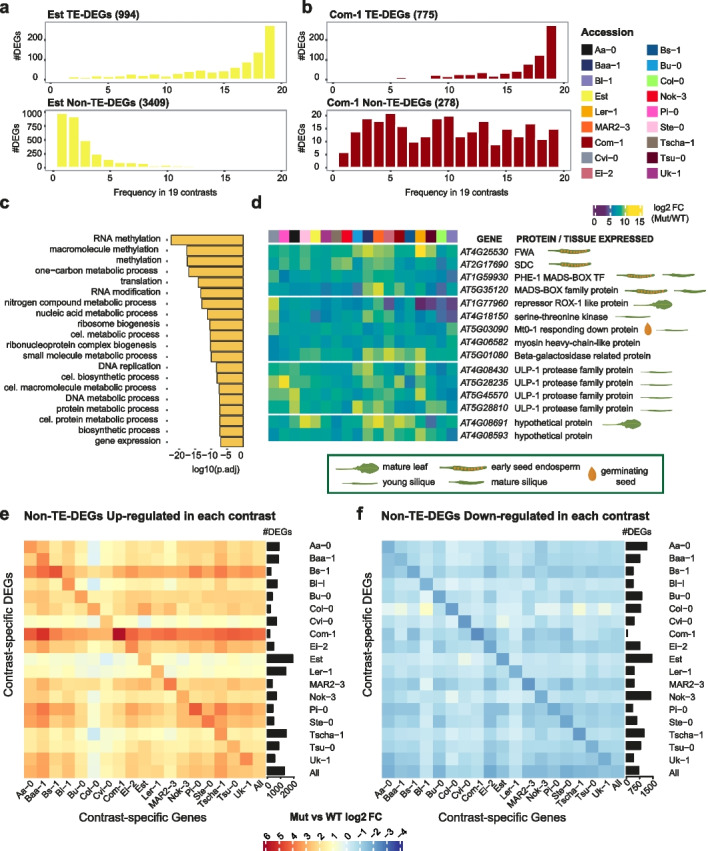
Qualitative and quantitative comparisons of accession-specific DEGs. **a, b** Frequency spectrum of Est and Com-1 TE-DEGs and Non-TE-DEGs across all other accessions**. c** The top 20 GO terms enriched for 983 DEGs unique to Est. **d** Heatmap of log_2_(fold change) of 15 universal Non-TE-DEGs across all accessions, grouped by protein function categories. TAIR10 gene names, encoded proteins and their preferential tissues of expression in wild-type (where known) are shown. **e, f** Heatmaps of average log_2_(fold change) for all Non-TE-DEGs from one accession in each of the other accessions. Barplots on the right indicate the absolute frequency of Non-TE-DEGs in each accession

We next generated a frequency spectrum to examine the overlap of DEGs between accessions. When we focused on the two extreme accessions, Est and Com-1, a great majority of TE-DEGs were shared across most contrasts, while the picture for Non-TE-DEGs was very different. While almost 30% of the 3409 Non-TE-DEGs in Est were not detected in any other accession, 98% of the 278 Com-1 Non-TE-DEGs were found in at least one other accession (Fig. 2a,b, Additional file 5: Fig. S8). The 983 unique Non-TE-DEGs in Est were enriched for functions with a common theme of RNA and DNA metabolism (Fig. 2c), compatible with a scenario in which mis-regulation of one or a few specific master regulators had led to expression changes in numerous Non-TE-DEGs in Est.

When overlaying DEGs from the 18 accession-specific *met1*-against-wild-type comparisons and the all-*met1*-against-all-wild-type comparison, we found 291 universal DEGs (Additional file 8: Dataset S5), albeit with the extent of expression change in *met1* mutants differing considerably across accessions (Fig. 1g, Additional file 5: Fig. S6). Only 15 of the universal DEGs were Non-TE-DEGs (Fig. 2d), and eight of these 15 genes are strongly expressed in siliques in Col-0 wild-type plants [39]. We had measured gene expression in rosette leaves, and many of these genes were expressed at low levels in our wild-type samples, but strongly upregulated in *met1* mutants. Included among the 15 genes were three maternally imprinted genes, *FWA*, *SDC*, and *AT1G59930* [40, 41], along with *AT5G35120*, which we suspect may be maternally imprinted as well, given its sequence similarity with *AT1G59930* and selective expression in the endosperm. Ectopic *FWA* expression is known to delay flowering in both Col-0 and Ler-1 accessions [42, 43], while ectopic activation of *SDC* has been shown to lead to dwarfism and leaf curling in Col-0 [41, 44]. This is consistent with the whole-plant phenotypes described in detail below. Four consistently observed DEGs encoded an ULP-1 protease family domain. *ULP-1* sequences have been found to be associated with *Mutator*-like TEs in *Cucumis melo* (“CUMULEs”), although there is no evidence that similar CUMULEs in rice or *A. thaliana* assist in TE mobilization [45]. Because these genes are not annotated as “TE genes” in TAIR10, they are included in our list of Non-TE-DEGs.

Finally, we assessed to what extent DEGs from one accession changed in the other accessions. Genes identified as up- or downregulated Non-TE-DEGs after loss of MET1 in one accession changed on average almost always in the same direction in each of the other accessions, but did so less strongly (Fig. 2e, f). TE-DEGs on the other hand were similarly upregulated in all accessions, but downregulated TE-DEGs were often variably expressed in other accessions (Additional file 5: Fig. S9). This finding is consistent with the idea that MET1 activity often homogenizes gene expression levels in different genetic backgrounds.

### *met1* mutants exhibit reduced DNA methylation and increased overall chromatin accessibility

To investigate how much of the variation in DEGs across accessions arose from differences in DNA methylation and chromatin architecture, we characterized the methylomes and chromatin accessibility in our collection of *met1* mutants and corresponding wild-type lines. As expected from the knockout of *MET1*, cytosine methylation in the CG sequence context was drastically and consistently reduced in homozygous *met1* mutants, dropping to an average genome-wide level of 0.2%, representing a 98.5% decrease from mean wild-type levels (Additional file 5: Fig. S10). Cytosine methylation in non-CG contexts was moderately affected in first-generation *met1* mutants, being on average 6.8% higher in the CHG context and 21% lower in the CHH context. Second-generation *met1* mutants of Tsu-0 and Tscha-1 had greatly increased CHG methylation, both relative to first-generation *met1* mutants and wild-type parental lines (Additional file 5: Fig. S10). This increase in methylation during successive rounds of inbreeding has previously been described for *met1-*3 mutants in the Col-0 accession [11].

**Fig. 3:**
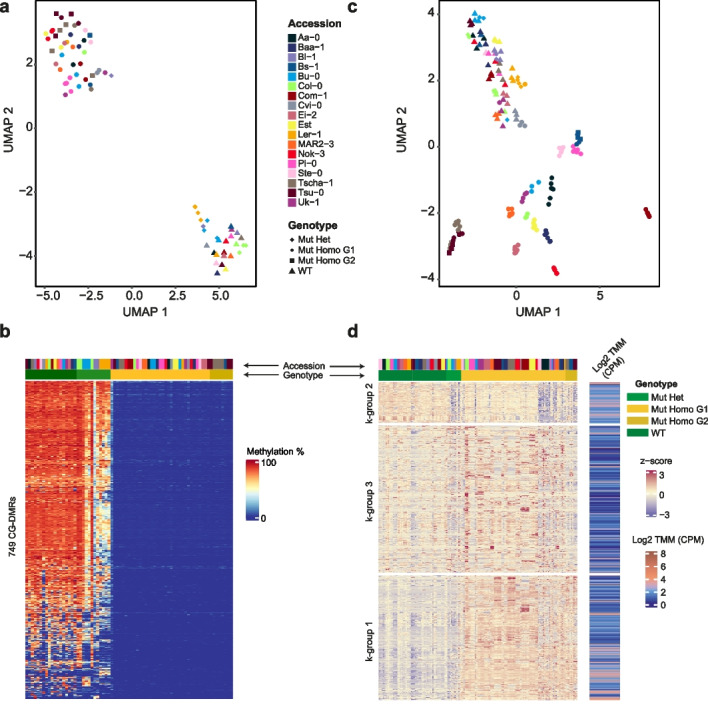
Reduced CG methylation and increased chromatin accessibility in *met1* mutants. **a, b** UMAP representation and heatmap of CG methylation levels in wild-type plants and *met1* mutants across 749 CG-DMRs (from a total of 2388 CG-DMRs). **c** UMAP representation of chromatin accessibility in log_2_(CPM) of 9505 highly variable dACRs (HV-dACRs) across wild-type plants and *met1* mutants. **d** Heatmap of *z*-scaled values of 9505 HV-dACRs grouped by *k*-means clustering, with mean accessibility for each dACR indicated on the right. TMM, trimmed mean of M-values. CPM, counts per million

Because overall methylation patterns were greatly altered in *met1* mutants, we wanted to closely examine genomic regions with the most significant changes in methylation. We contrasted 73 methylomes including both *met1* mutants and the wild-type parents from all 18 accessions (“Methods”) to identify differentially methylated regions (DMRs) in the CG, CHG, and CHH contexts (Fig. 3a, b, Additional file 5: Fig. S11, Additional file 9: Dataset S6, Additional file 10: Dataset S7, Additional file 11: Dataset S8). The 2388 CG-DMRs overlapped with the vast majority of the 350 CHG-DMRs and 1023 CHH-DMRs. Approximately half of all CG-DMRs overlapped with TE sequences, a quarter overlapped TE genes, while 42% overlapped Non-TE genes (Additional file 5: Fig. S10, Additional file 2: Table S2).

While most CG-DMRs in *met1* mutants retained minimal or no CG methylation, the extent of reduction in methylation differed across accessions, consistent with accession-specific methylation patterns in the presence of MET1 [16] (Fig. 3a, b, Additional file 5: Fig. S12). To assess the functional consequences of this variation, if any, we asked how loss of cytosine methylation impacted genome-wide chromatin architecture in each accession. To this end, we identified accessible chromatin regions (ACRs) by ATAC-seq in all *met1* mutants and the corresponding wildtypes. With this analysis, which allowed us to define differential ACRs (dACRs) with accessibility changes in at least two accessions (“Methods,” Additional file 12: Dataset S9), we could focus on 9505 highly variable dACRs (HV-dACRs) with particularly stark variation in accessibility across accessions and genotypes (“Methods,” Additional file 13: Dataset S10). We visualized variation in accessibility levels at these HV-dACRs using UMAP (“Methods”), which similarly to the RNA-seq data (Fig. 1b) revealed two distinct clusters of wild-type plants and *met1* mutants (Fig. 3c), with wild-type plants from different accessions being more similar to each other than *met1* mutants from different accessions. Approximately one third of all HV-dACRs overlapped in position with Non-TE genes, 24% with TE genes, and 62% with TE sequences (Additional file 5: Fig. S13, Additional file 2: Table S2).

Genome wide, the chromatin of *met1* mutants was more accessible than chromatin of wild-type plants, and this difference was particularly pronounced for two subgroups, k-groups 1 and 3, of HV-dACRs identified by *k*-means clustering (Fig. 3d). Across all accessions, approximately one third (31%) of CG-DMRs overlapped with HV-dACRs, but at the same time, most HV-dACRs (88%) did not overlap with DMRs (Additional file 2: Table S2), indicating that the vast majority of HV-dACRs appeared due to *trans* effects of methylation changes in the genome.

To see whether epigenetic profiles at TEs, which included both TE genes and other TE sequences, differed from Non-TE genes, we averaged methylation levels using genome-wide methylated cytosines in all contexts (“Methods”) and chromatin accessibility levels using 34,993 consensus ACRs (“Methods”) for all *met1* mutants and wild-type plants across 31,189 TEs and 29,699 Non-TE genes including 1 kb flanking sequences (Additional file 5: Fig. S14). Genome-wide chromatin accessibility patterns in *met1* mutants mirrored cytosine methylation levels, with increases in accessibility accompanied by decreases in methylation for most accessions. This pattern was much more pronounced in magnitude over TEs (Additional file 5: Fig. S14a) than in Non-TE genes (Additional file 5: Fig. S14b). These observations once again demonstrate that TEs are highly sensitive to the absence of MET1, in agreement with TE genes being strongly upregulated in *met1* mutants.

Finally, we asked how much of the expression changes at DEGs were explained by these epigenetic alterations. We focused on Est and Com-1, the two accessions with the highest and lowest number of DEGs. While TE-DEGs behaved similarly in the two accessions, Non-TE-DEGs showed contrasting patterns, with accessibility differences between *met1* mutants and wildtypes being on average much smaller in Est than in Com-1 (Additional file 5: Fig. S14c), confirming a complex relationship between methylation, chromatin accessibility and gene expression changes at Non-TE-DEGs, which in turn leads to very different numbers of Non-TE-DEGs in different accessions.

### Non-TE-DEGs show varying methylation and chromatin accessibility profiles across accessions

Observing that differential gene expression can be accompanied by epigenetic changes, we quantitatively assessed mutual relationships of cytosine methylation and chromatin accessibility with gene expression, having analyzed all of the three factors from the same individuals across our collection of *met1* mutants and wildtypes.

To investigate whether the presence or absence of methylation within or in proximity to a gene could influence its expression level, we examined how DEGs were regulated in the presence of a DMR. We first defined a consensus set of 7132 DEGs from all 18 accessions (“Methods,” Additional file 5: Fig. S15, Additional file 14: Dataset S11, Additional file 15: Dataset S12), containing TE-DEGs (1401) and Non-TE-DEGs (5731) and intersected their genomic coordinates with 1569 CG-DMRs with sufficient methylation calls across all samples that were identified from all mutant and wild-type samples (“Methods,” Additional file 16: Dataset S13).

While only a small fraction of consensus DEGs were located close to a CG-DMR (21% of TE-DEGs and 7% of Non-TE-DEGs) (Additional file 2: Table S2), the converse was also true: only a minority of CG-DMRs was found next to DEGs (21% next to TE-DEGs and 27% to Non-TE-DEGs). Local differences in CG methylation are thus neither necessary nor sufficient for differences in gene expression between wild-type and *met1* mutant plants. At the loci where both expression and CG methylation were altered, we examined expression counts and methylation levels in homozygous *met1* mutants and in corresponding wild-type plants from 17 accessions. As a control, we randomly sampled Non-DEGs (genes that were not differentially expressed; “Methods”) that positionally overlapped with DMRs.

Most TE-DEGs with CG-DMRs in either their extended gene bodies (“Methods,” Additional file 5: Fig. S16, Additional file 5: Fig. S17c) or 1.5 kb up- or downstream sequences (“cis” regions) (“Methods,” Additional file 5: Fig. S16, Additional file 5: Fig. S18c) had lost CG methylation in *met1* mutants*.* These TE-DEGs were upregulated in all *met1* mutants, albeit to a different extent in each accession. Non-TE-DEGs, which were already observed to be very variable in their differential expression levels, exhibited a wide gradient of methylation differences between *met1* mutants and the respective wild-type parents (ranging from −100 to +10%). This was observed both when DMRs were located in extended gene bodies (Additional file 5: Fig. S17a) and in 1.5 kb up- or downstream sequences (Additional file 5: Fig. S18a). In both cases, one group of Non-TE-DEGs was strongly upregulated and had highly reduced methylation in *met1* mutants, suggesting that methylation could have a strong effect on gene regulation at these genes. There was also another group of more moderately up- or downregulated Non-TE-DEGs with negligible methylation changes in *met1* mutants. On examining the accession-of-origin of each of these Non-TE-DEGs, we found that the same gene in different accessions could be found in either of the groups identified above, indicating considerable epigenetic plasticity across accessions.

**Fig. 4:**
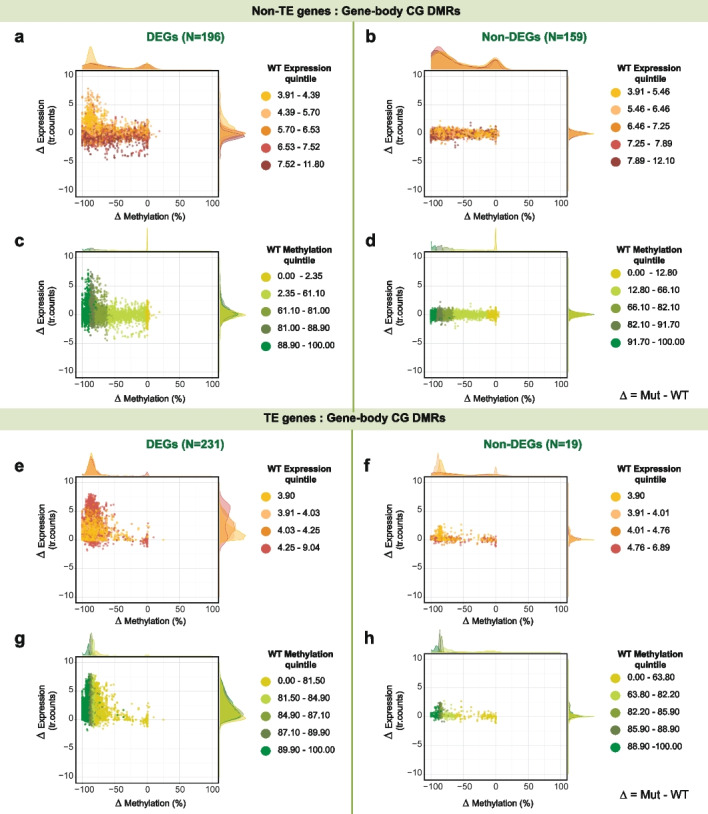
CG-DMRs in gene bodies of Non-TE-DEGs and TE-DEGs. Differences in CG methylation between *met1* mutants and wild-type plants plotted against differences in gene expression. Dots are colored by wild-type expression quintiles (**a,b,e,f**) and wild-type methylation quintiles (**c,d,g,h**) with density distributions shown on top and left. Expression levels are represented as transformed read counts (tr. counts) and methylation levels as % CG methylation in CG-DMRs

Consequently, we asked whether parental methylation and expression state can predict epigenetic and transcriptional response in *met1* mutants. DEGs classified in the top quintiles of wild-type CG methylation levels (“Methods”) were more likely to increase in expression than DEGs from the bottom quintiles, which were similarly likely to be up- or downregulated in *met1* mutants (Fig. 4c, g, Additional file 5: Fig. S19c, Fig. S19g). In terms of expression changes, we found that DEGs from the lowest quintile of expression counts in the wild-type parents were the ones that were the most upregulated in *met1* mutants (Fig. 4a, e, Additional file 5: Fig. S19a, Fig. S19e). These observations are consistent with high levels of CG methylation in the wild-type state serving to silence genes.

In *A. thaliana*, many constitutively active genes are marked by gene body CG methylation (gbM) [46] and the methylation levels of these genes are known to vary in tandem with their differential expression across *A. thaliana* accessions [22, 23]. Among Non-TE-DEGs with gene body CG-DMRs, 91 out of 196 genes (46%) were methylated in the wild-type state (“gbM like” genes; “Methods”) in at least one accession. Most of these “gbM-like” genes were downregulated in *met1* mutants (Additional file 5: Fig. S20a). When we examined the larger set of all Non-TE genes (19,979), we found three genes that were consistently gbM-like in wildtypes of 17 accessions, but with highly reduced methylation in homozygous *met1* mutants. These were *AT5G20490* (*XIK*, encoding a myosin family protein involved in root hair growth and trichome development), *AT5G37130* (encoding a prenylyltransferase superfamily protein), and *AT5G44800* (*PICKLE*, encoding a protein that affects histone methylation levels and impacts floral meristem identity) (Additional file 5: Fig. S20b). Only *AT5G37130* was a consensus Non-TE-DEG (being significantly downregulated in *met1* mutants in Aa-0, Com-1, Pi-0 and Cvi-0) (Additional file 5: Fig. S20c). Incidentally, genes that were highly methylated and weakly expressed in wildtypes (exhibiting TE methylation characteristics in the CG context or “CG teM-like” genes; “Methods”) were always upregulated in *met1* mutants (Additional file 5: Fig. S21), similarly to TE-DEGs. The two groups of genes, with gene body or TE methylation in the CG context, did not overlap.

We next carried out a similar quantitative analysis to examine the relationship between differential expression changes and differential chromatin accessibility at DEGs (“Methods,” Additional file 5: Fig. S22). Approximately 37% of all consensus DEGs (“Methods”) were associated with HV-dACRs within 1.5 kb upstream and downstream of their gene body (Additional file 2: Table S2). While chromatin accessibility increased almost proportionally with expression in *met1* mutants for TE-DEGs, increased accessibility could be associated with, but did not necessitate an increase in gene expression for Non-TE-DEGs (Additional file 5: Fig. S23, Additional file 5: Fig. S24). Accessible chromatin is well known to favor gene transcription by facilitating transcription-factor binding [47, 48] although there is also evidence that inaccessible regions can occur in some long and highly transcribed genes [49]. Together, these observations point to complex interactions between gene expression and chromatin accessibility levels in *cis*, especially at Non-TE-DEGs.

### *MET1* can have indirect effects on the expression of Non-TE genes

**Fig. 5:**
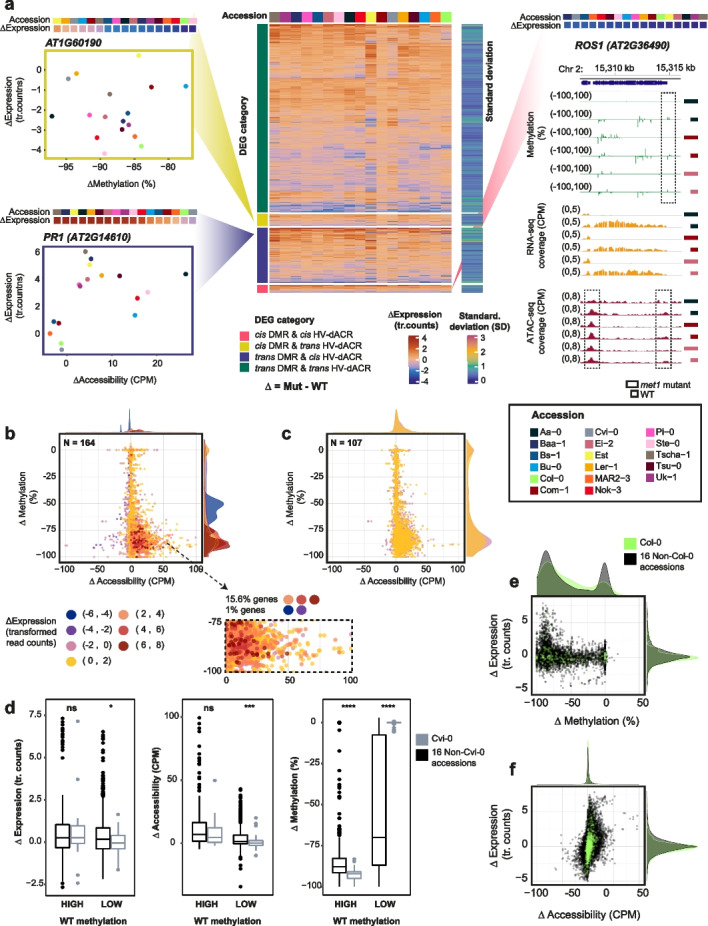
Non-TE-DEGs in *met1* mutants can have different epigenetic states in different accessions. **a** Heatmap of expression changes across 5731 Non-TE-DEGs in 17 accessions, with an adjacent heatmap showing variance expressed as standard deviation (SD) across accessions, and scatterplots of changes in expression and accessibility in representative genes, *AT1G60190* and *PR1*, from two different DEG categories (based on overlap with *cis* CG-DMRs and HV-dACRs). A genome browser screenshot of ATAC-seq, RNA-seq, and BS-seq data in three accessions is shown for a third example gene locus, *ROS1*, harboring both *cis* DMRs and *cis* dACRs. **b** Scatterplot of changes in chromatin accessibility and methylation in Non-TE-DEGs across 17 accessions. Colors and density distributions represent custom bins of expression changes. A closeup of a selected region is shown below. **c** Scatterplots similar to **b** for Non-TE genes. **d** Boxplots showing *MET1*-dependent changes in chromatin accessibility, gene expression, and CG methylation of genes that are weakly (“LOW”) or highly (“HIGH”) methylated in wild-type Cvi-0. The same genes are compared for Cvi-0 (dark green) and 16 other accessions (gray). **e** Scatterplot of changes in methylation and expression in Non-TE-DEGs with gene body CG-DMRs, colored by DEGs specific to Col-0 (black) against the same genes in other accessions (yellow). **f** Scatterplot of changes in chromatin accessibility and expression in Non-TE-DEGs carrying *cis* dACRs, colored by DEGs specific to Col-0 (black) against the same genes in other accessions (yellow). Expression levels are represented as transformed read counts (tr. counts); chromatin accessibility levels as TMM (trimmed mean of M-values) normalized values in counts per million (CPM), and methylation levels as % CG methylation

Since variation in the response of gene expression, methylation and chromatin accessibility to loss of *MET1* was most apparent for Non-TE-DEGs, we focused on these genes to explore the nature of their epigenetic plasticity. Among 5731 consensus Non-TE-DEGs that varied in their expression response across accessions (Fig. 5a), 21% had differentially accessible chromatin regions (HV-dACRs) in their vicinity, but lacked a *cis* CG-DMR (“*cis*” here including the gene body) (Additional file 2: Table S2). Conversely, a minority of Non-TE-DEGs, 255 (5%) had *cis* CG-DMRs but no nearby HV-dACRs, and a majority of Non-TE-DEGs, 4105 (72%) had neither a nearby HV-dACR nor CG-DMR (Additional file 2: Table S2). Closer inspection of several genes from these categories, *AT1G60190*, *PR1*, *ROS1* (Fig. 5a), showed that the relationship between altered gene expression, CG methylation, and accessibility in *met1* mutants varied substantially, both across genes and accessions.

To focus on close-range interactions between changes in methylation, chromatin accessibility, and gene expression at the same locus, we examined Non-TE genes associated with both *cis* HV-dACRs and CG-DMRs—comprising 164 DEGs, which we compared with 107 randomly sampled Non-DEGs (“Methods”), by overlaying methylation, accessibility, and gene expression data for each gene across 17 accessions. We observed that 3073 genes (in 17 accessions) with strongly reduced CG methylation (>75% methylation reduction) had a tendency to have increased chromatin accessibility. Of these, 15.6% were strongly upregulated in *met1* mutants, while only 1% were strongly downregulated (Fig. 5b). Incidentally, strongly upregulated genes in this group also stood out from the rest because they exhibited moderately increased accessibility in *met1* mutants, consistent with methylation changes being directly responsible for induction of expression (Fig. 5b). For both DEGs and Non-DEGs (Fig. 5b, c), there were many other different combinations of methylation and accessibility states, and these did not always cluster by degree of expression change. Together, these observations suggested the presence of multiple epigenetic states, both for different genes in the same accession and for the same gene in different accessions.

We next examined Non-TE genes (271 genes including 164 DEGs and 107 Non-DEGs) as a group for accession-specific epigenetic patterns, first comparing the methylation levels of these genes in wildtypes and mutants. Cvi-0 had the highest fraction of genes with minimal reduction in methylation in *met1* mutants (Additional file 5: Fig. S25a). This was explained by Cvi-0 wild-type having already many genes with low methylation levels, limiting the extent of any further reduction in methylation (Additional file 5: Fig. S25a). We asked how genes with low CG methylation in Cvi-0 wild-type, that is, genes with methylation levels that could not be reduced much further by loss of *MET1*, fared in other accessions. While the average reduction in methylation level was greater in other accessions, as expected, changes in accessibility and expression level after inactivation of *MET1* were similar in magnitude when compared to Cvi-0 (Fig. 5d). This observation provides further support for genome-wide hypomethylation indirectly affecting the expression of many genes. Finally, comparing the relationship between *MET1-*dependent changes in methylation, chromatin accessibility, and gene expression in the reference accession Col-0 with changes in other accessions confirms that Col-0 is not particularly representative of *A. thaliana* accessions at large (Fig. 5e, f).

### *met1* mutants express signatures of known epialleles

Many of our *met1* mutants had strong methylation and expression changes at well-known loci sensitive to epigenetic regulation, such as *FWA* [42] (Additional file 5: Fig. S25c,d), *SDC* [44] (Additional file 5: Fig. S26), the *PAI* genes [50], *IBM1* [51], *SNC1* (with alleles similar to the *bal* variant of *SNC1*) [52], the *ROS1* demethylase, which is known to function as a methylation sensor [11, 53, 54] (Fig. 5a, Additional file 5: Fig. S25b), *AG* [4] (Additional file 5: Fig. S27), and *SUP* (with alleles similar to the *clark-kent* variant of *SUP*) [55] (Additional file 5: Fig. S28). New epialleles at several of these loci have been reported before in Col-0 and C24 *met1* mutants [3, 56]. We observed a variety of methylation patterns at these loci depending on the accession of origin, with considerable differences in chromatin accessibility and gene expression across accessions; several examples at the *FWA* locus are shown in Additional file 5: Fig. S25c,d. As seen before [11], some epialleles arose only in second-generation *met1* mutants (Additional file 5: Fig. S27), suggesting that epialleles continue to accumulate in the absence of *MET1* during inbreeding.

### *met1* mutants vary in phenotypes and segregation distortion

**Fig. 6:**
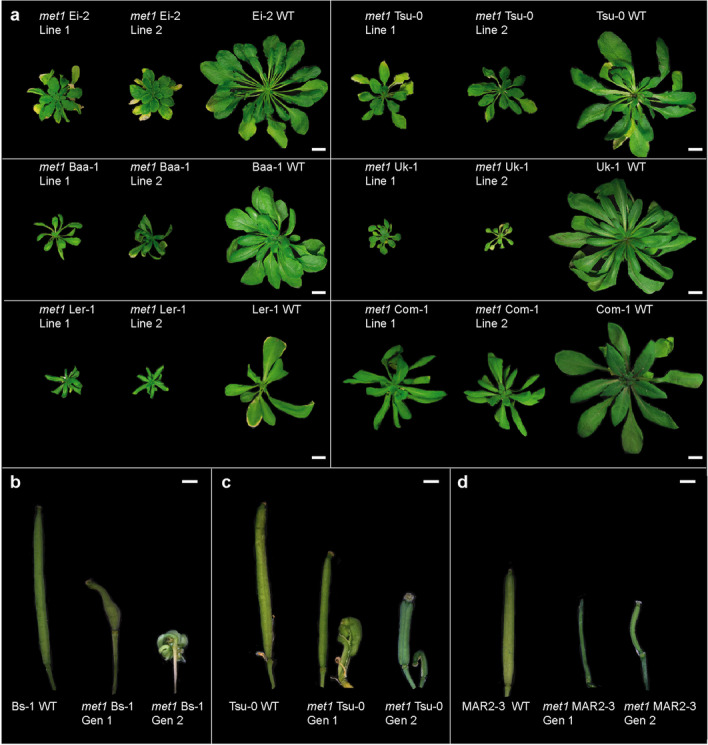
Rosette and silique morphology of *met1* mutants. **a** Representative images of two independently derived mutants and the corresponding wild-type (WT) for six accessions at 6 weeks post germination; scale bars represent 1 cm. **b**–**d** Silique morphology in three accessions. Scale bars represent 1 mm. Gen1, first-generation homozygous mutants; Gen 2, second-generation homozygous mutants

**Fig. 7:**
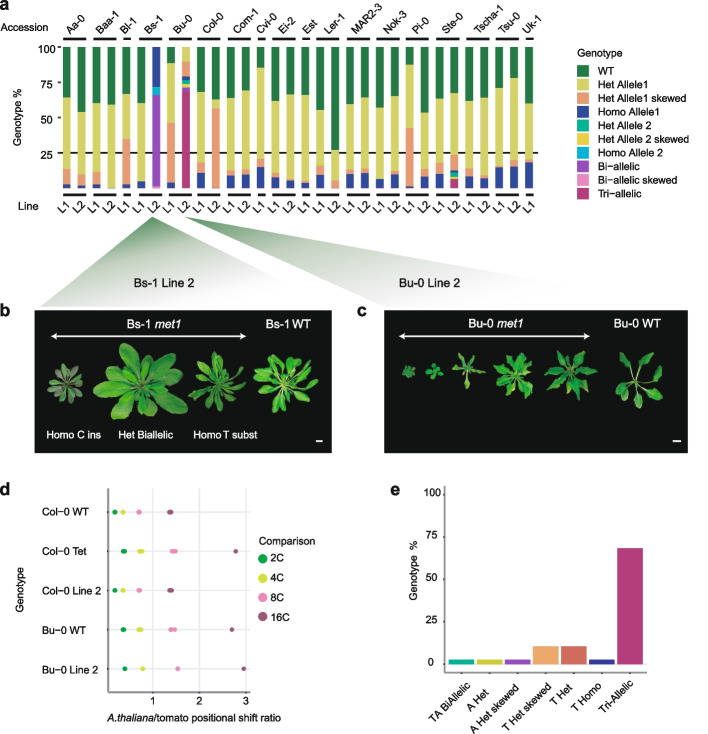
Segregation distortion in *met1* mutants. **a** Proportions of wild-type and *met1* mutant genotypes in progeny of heterozygous individuals. L1, L2, Line 1, Line 2. **b** Different phenotypes in *met1* Bs-1 Line 2. “Ins” refers to “insertion” and “subst” refers to “substitution.” **c** Phenotypic diversity in Bu-0 Line 2. **d** Endopolyploidy peak position ratios (from flow cytometry profiles) in Bu-0 and Col-0 lines relative to tomato internal standard. “Col-Tet,” Col-0 tetraploid line. **e** Fractions of segregating genotypes in *met1* Bu-0 Line 2 progeny. Scale bars in **b** and **c** = 1 cm

Compared to their wild-type parents, homozygous *met1* mutants were dwarfed, flowered late, and had altered rosette leaf architecture—although to different degrees in each accession (Fig. 6a, Additional file 5: Fig. S29-S30). *met1* mutants also suffered from silique abnormalities, in some cases affecting fertility (Fig. 6b–d). Additionally, we observed during the preparation of ATAC-seq libraries that *met1* mutant nuclei had lower levels of endopolyploidy (Additional file 5: Fig. S31). Homozygous mutants were underrepresented in the progeny of heterozygous parents, consistent with reduced transmission of *met1* alleles, as described previously [6, 57]. To estimate the extent of segregation distortion, we grew up to 96 progeny of heterozygous mutants and genotyped all individuals. In all accessions, homozygotes were underrepresented (Fig. 7a, Additional file 17: Table S3), from 2% in Aa-0 to 18% in Uk-1.

Bs-1 Line 2 included two different types of homozygous mutants that were phenotypically distinct, with the frameshift allele, which we used for the genomic analyses described above, causing more severe phenotypic defects and being more strongly underrepresented in a segregating populations (Fig. 7b, Additional file 17: Table S3). Bu-0 Line 2 and Ste-0 Line 2 segregated individuals with different combinations of two mutant alleles along with a wild-type allele, pointing not only a bi-allelic origin of the *met1* alleles but also a change in the generative ploidy level. Genotyping by sequencing also revealed heterozygous individuals with a skewed ratio of reads for the wild-type and mutant alleles, especially in Bu-0 Line 1, Pi-0 Line 1, Col-0 Line 2, and Bl-1 Line 1 (Additional file 17: Table S3). Since heterozygotes of these four lines also exhibited phenotypic variation (Fig. 7c, Additional file 5: Fig. S32), we suspected that there was variation in ploidy. Both Bu-0 Line 1 and Line 2 as well as wild-type Bu-0 plants used in this study turned out to be tetraploid, possibly explaining the altered segregation ratios (Fig. 7d, e). However, other lines with skewed heterozygous allele frequencies were derived from diploid parents (Additional file 5: Fig. S32), awaiting a more comprehensive explanation of genotypic and phenotypic variation in these lines.

## Discussion

The genomes, methylomes, and transcriptomes of different *A. thaliana* accessions can vary substantially [13, 16], and studying their interplay has often focused on TEs, which are silenced by DNA methylation [14, 19]. DNA methylation is also prevalent throughout euchromatin, both near and inside protein-coding genes that are not associated with TEs [23, 58]. MET1 is well known to establish methylation in the predominantly occurring CG nucleotide context and cause major phenotypic consequences when its function has been lost. The availability of a collection of *met1* mutants in several accessions enables the investigation of gene expression diversity associated with variation in the parental genomes and epigenomes. We analyzed first- and second-generation homozygous *met1* mutants, finding that the majority of qualitative and quantitative variation in gene expression across accessions arises from genes that are not associated with TEs (Non-TE-DEGs).

The absence of CG methylation can disturb the regulatory balance of hundreds to thousands of genes, and that it does so to a remarkably different extent in genetically diverse backgrounds. Moreover, the comparison of transcriptome diversity across accessions between wild-type and *met1* mutants shows that CG methylation by MET1 can mask underlying genetic diversity at some genes, but at the same time increase expression diversity at another, albeit smaller set of genes.

When analyzing a consensus set of DEGs from all accessions together, we systematically find that the effects of MET1 inactivation can be linked to the initial epigenetic state of the wild-type parent. Genes that are highly CG methylated, expressed at low levels, and have inaccessible chromatin in wild-type plants are the ones that are most likely to become strongly expressed and to have greatly increased chromatin accessibility in the corresponding *met1* mutants. This pattern is typical for most TE-DEGs, but also seen at some Non-TE-DEGs. From first principles, genes not associated with TEs are much more likely than those genes associated with TEs to change in expression due to indirect effects of *MET1*, and this is confirmed by Non-TE-DEGs being more variable in their methylation and accessibility changes in *met1* mutants. This is also associated with more variation in gene expression changes: While TE-DEGs are almost always upregulated, Non-TE-DEGs change in both directions in *met1* mutants.

Genomic regions that become more accessible in Col-0 *met1* mutants have been associated with multiple gene groups, classified by their expression changes [10]. In our study, we find that this association can be multi-layered, varying by gene, initial methylation level in wild-type, and overall genetic background. Our most important conclusion is perhaps that CG methylation, which requires *MET1* activity, cannot be simply thought of as a factor that masks genetic differences or that increases expression diversity beyond genetic variation, but that its effects are highly context-dependent.

Our results show that the same gene across different wild-type backgrounds can not only exist in multiple epigenetic states, but also that it can vary in its regulatory response to genome-wide CG hypomethylation, thereby unveiling distinct associations between methylation, chromatin accessibility, and gene expression. For example, the study by Zhong and colleagues [10] demonstrated how local changes in methylation were sufficient to alter chromatin accessibility at the *FWA* epiallele in the reference accession Col-0. Upon examining the *FWA* locus in homozygous *met1* mutants in different accessions, we find that the increases in accessibility can vary substantially despite a similar degree of CG hypomethylation in each *met1* mutant, starting from different initial parental methylation levels (Additional file 5: Fig. S25). Additionally, the relationship between accessibility changes and expression changes is non-linear for this gene (Additional file 5: Fig. S25), suggesting that either variation in cis-regulatory sequences or trans regulators make an important contribution to *FWA* expression beyond methylation.

*FWA* is only one example from 11,675 unique DEGs in our set of *met1* mutants from 18 accessions (compared to 1759 DEGs, when comparing only Col-0 wild-type and *met1*). That only 291 DEGs among these 11,675 unique DEGs are universal across all accessions highlights the differential sensitivity of each genetic background to methylation-dependent changes in gene expression. Accession-specific DEGs include developmental and epigenetic regulators such as *ROS1*, *IBM1*, and *SUVH3*, many of which feature well-examined epialleles. We note that because we only sampled leaves, we almost certainly have not discovered all genes that are sensitive to loss of *MET1*, and indeed many other tissues besides leaves are phenotypically affected in *met1* mutants.

Methylation and chromatin accessibility changes are clearly neither necessary nor sufficient for changes in gene expression in *met1* mutants, again revealing how methylation and chromatin architecture must interact with cis-regulatory sequences to effect gene expression [59]. Genetic variation in both regulatory and non-regulatory sequences can also influence gene expression, a factor that we have ignored so far. In maize, TE insertion polymorphisms can affect both chromatin accessibility state as well as expression levels of adjacent genes [60]. Genetic variants within conserved non-regulatory sequences in maize [61] and *A. thaliana* [62] accessions can be associated with gene expression, and at least in some cases these sequences overlap with accessible chromatin regions. In other cases, structural variants within gene bodies may influence expression independently of underlying epigenetic variation [22]. De novo genome assemblies in five *A. thaliana* accessions have shown that highly diverged sequences within DNase-I hypersensitive sites are often associated with differential gene expression, even though most chromatin architecture changes are largely independent of genetic variation [63]. All of these examples demonstrate that high-quality genome assemblies of the 18 accessions used in our study will almost certainly provide additional insight into the interplay between the genome, epigenome, and transcriptome at the intraspecific level. More importantly, genome assemblies will help identify which signals of reduced accessibility or gene expression are merely due to absence of the underlying DNA sequences, instead of more subtle sequence variation or even *trans* effects.

It will also be interesting to determine whether genes with altered methylation and chromatin accessibility are more likely to change in their expression during further cycles of propagation. Since TEs are known to mobilize upon inbreeding of epigenetic mutants, high-quality reference genomes will also be useful to identify induced copy number variation and presence-absence variation of TEs in different accessions. In addition, deeper investigation of non-CG and residual CG methylation in *met1* mutants, catalyzed by other methyltransferases and compensatory pathways, examined together with chromatin marks, may further improve our understanding of methylation-induced gene regulation.

A wide range of silique abnormalities and distorted segregation ratios observed in our *met1* mutants indicates that the absence of MET1 function may be detrimental for gametogenesis, fertilization or post-zygotic development, at least in some accessions. Among the accessions used in our study, Ler-1 and Tsu-0 carry a microRNA haplotype that impairs silencing of specific TEs in male gametes (Ler-0 *MIR845* haplotype [64]). Furthermore, epigenetic variation across Col-0, Ler-1, and Cvi-0 has been shown to impact seed development due to differential imprinting [65, 66]. For example, the comparatively higher fertility of *met1* mutants in Cvi-0 could be explained by the natural hypomethylation of the *HDG3* (*AT2G32370*) locus in wild-type Cvi-0 plants (Additional file 5: Fig. S28), thereby reducing dramatic effects in seed size as observed in mutants of other accessions. Future studies on the epigenetic and epigenomic landscape of gametophytic, embryonic, and endosperm tissue of our *met1* mutants will shed light on how hypomethylation in somatic cells can impact fertilization processes.

## Conclusion

The absence of *MET1* in *A. thaliana* has long been known to affect the chromatin landscape [7, 8, 10, 11]. Here we highlight the value of studying accessions beyond the reference accession Col-0, finding not only that CG methylation, gene expression, and chromatin accessibility can widely vary, but also that the impact of *MET1* can be much greater in other accessions, especially at genes that are not associated with TEs.

Our *met1* mutant collection provides future opportunities for investigating how epigenetic and epigenomic regulation at individual loci are fine-tuned across accessions to determine plant phenotype. Another opportunity will be to study the TE mobilization landscape in these mutants, and to ask whether sites with newly inserted or newly excised TEs have similar epigenetic states across different genetic backgrounds and how these in turn affect adjacent protein-coding genes [60]. Finally, while the resources and insights we have generated have already improved our understanding of the variation in epigenetic and epigenomic regulation that evolution has produced, high-quality genome assemblies of the studied accessions will make our resources even more valuable.

## Methods

### CRISPR/Cas9 knockout of *MET1* in 18 *A. thaliana* accessions

Using a plant molecular cloning toolbox [28], a supermodule destination binary vector carrying a plant-codon optimized *Cas9* driven by a *UBQ10* promoter was cloned with a single guide-RNA (gRNA) targeting the *A. thaliana MET1* (*AT5G49160* ) gene. The gRNA was designed using the CRISPR design tool in *Benchling* (www.benchling.com) targeting a 20-bp region in exon 7 of *MET1* (Additional file 1: Table S1), which is the same exon where previously described *met1-3* mutants are known to harbor a T-DNA insertion [6]. This exon is present in the catalytic domain of the protein and harbors a motif that is a binding site for cytosine nucleotide substrates [67]. Eighteen early-flowering *A. thaliana* accessions were transformed with the above CRISPR construct by *Agrobacterium-*mediated floral dipping [68], carried out twice with a 7–10-day interval. Seeds of primary transformants (T_1_) were screened for the presence of the transgene by selecting for the mCherry fluorescence marker, and sown on soil. These T_1_ plants were subjected to heat treatment cycles for enhancing Cas9 activity [69]. Genotyped lines carrying a mutation in the gRNA target region were propagated to the T_2_ generation after segregating the transgene (by selecting for non-mCherry seeds), followed by identification of lines carrying heritable heterozygous mutations. One to two heterozygous T_2_ lines per accession were further subjected to one or two more rounds of propagation to identify first-generation homozygous plants in the segregating progeny.

### Genotyping transformants and identification of homozygous mutants

First- and second-generation homozygous mutants were genotyped either using Sanger sequencing of a 649 bp PCR-amplicon, or by amplicon-sequencing of a 152-bp PCR-amplicon (Additional file 1: Table S1), both covering the CRISPR guide-RNA target region. Most of the mutations identified in all mutants were single bp insertions or deletions that occurred within the first 4 bp from the 5′ end of the target region and disrupted the open reading frame due to frameshifting. Candidate homozygous mutants in segregating T_3_ populations were first identified by visual phenotyping, followed by genotyping.

### Plant growth conditions and tissue collection for large-scale sequencing

Seeds were sterilized by treatment with chlorine gas for 4 h, followed by stratification in the dark at 4**°**C for 4 days in 0.1% agar. All plants were grown in controlled growth chambers at 23 **°**C, long-day conditions (16 h light/8 h dark) with 65% relative humidity under 110 to 140 μmol m^−2^ s^−1^ light provided by Philips GreenPower TLED modules (Philips Lighting GmbH, Hamburg, Germany) with a mixture of 2:1 DR/W LB (deep red/white mixture with ca. 15% blue light) and W HB (white with ca. 25% blue light), respectively, and watered at 2-day intervals.

Since homozygous mutants from several accessions had reduced fertility and did not set sufficient seeds for further propagation, sampling for all sequencing experiments was carried out in the first homozygous generation. Segregating populations of T3 mutant plants were grown from 1 to 2 lines per accession, and homozygous individuals were marked by their distinct phenotype (as identified in previous growth experiments) and later confirmed by Sanger sequencing of DNA used for BS-seq libraries. Wild-type plants from the same accessions were grown in parallel to all mutant lines.

At 25 days after germination, three homozygous individuals per parental line per accession were collected as separate biological replicates, along with three wild-type individuals. Sampling involved the collection of two sets of rosette leaves from the same individual plant. One set of leaves was immediately frozen in liquid nitrogen containers (and subsequently at −80°C) to be homogenized and split for bisulfite sequencing and RNA-sequencing analysis. The second set of leaves were collected for ATAC-sequencing analysis and were subjected to syringe-infiltration with 0.1% formaldehyde in phosphate-buffer saline, followed by 0.125 M glycine in phosphate-buffer saline, washed with autoclaved water and dried before storage at −80°C. All tissue sampling and fixation was carried out within a 30-min time window.

### Bisulfite-seq library prep

Frozen leaf tissue from three biological replicates was mixed and grinded together. This powder was used for isolating genomic DNA using the DNeasy Plant Mini Kit (Qiagen). One hundred nanograms of this genomic DNA was subsequently used to prepare Bisulfite libraries with the TruSeq Nano kit (Illumina, San Diego, CA, USA) according to the manufacturer’s instructions, with the modifications used in [71]. The libraries were sequenced in paired-end mode, with approximately 8.5 million 150 bp reads/library on an Illumina HiSeq3000 instrument.

### Processing of Bisulfite-seq data and DMR calling

Raw BS-seq reads were aligned using Bismark with default parameters [72] and mapped to the *A. thaliana* (TAIR10) reference genome. The bisulfite conversion efficiency for each sample was estimated by evaluating the fraction of positions correctly called as unmethylated in the chloroplast genome. It was consistently above 99.6% in all samples. The mapping efficiency for all samples varied between 40 and 65%, with an average of 49% (Additional file 18: Dataset S14). Deduplicated bam files generated by Bismark were sorted and then processed using *MethylScore* (https://github.com/Computomics/MethylScore [73];) in the CLIP cluster of the Vienna Biocenter (VBC), to identify DMRs (differentially methylated regions) across all 73 samples (55 mutants and 18 wildtypes) with the following parameters: DMR_MIN_C=10 (minimum 10 cytosines in each DMR), DMR_MIN_COV=3X (minimum 3× coverage in each cytosine), MR_FREQ_CHANGE=20 (at least 20% of samples showing a change in MR frequency to be tested as a candidate DMR), CLUSTER_MIN_METH_DIFF=20 (which sets a 20% cutoff for methylation difference between clusters in the CG, CHG, and CHH contexts). All other parameters were based on default settings.

DMR coordinates were intersected with individual genome-wide cytosine methylation levels based on the *genome_matrix* file generated by MethylScore. A total of 2388 CG-DMRs (Additional file 9: Dataset S6), 350 CHG-DMRs (Additional file 10: Dataset S7), and 1023 CHH-DMRs (Additional file 11: Dataset S8) were called across 73 samples (55 mutants and 18 wildtypes). In some cases, a DMR was enriched for more than one context, resulting in partial redundancies. Subsequently, the three context-specific DMRs (CG, CHG, CHH) were evaluated for context-specific average methylation levels by intersecting with sample-specific cytosine methylation data. This was achieved using the *bedtools* software (v2.26.0) with the following command:

*bedtools map -a DMR_coordinates.bed -b methylated_cytosines_sampleX.bed -c 5 -o mean -nonamecheck -null "NA" -g TAIR10genomesize > DMR_Methavg_sampleX.bed*

Although the DMR calling was performed with a three-read cutoff for each cytosine in a DMR, there remained some samples which did not have sufficient coverage. Therefore, we retained the same DMRs, but calculated average methylation by lowering the cutoff to 2 reads per cytosine. For downstream analysis, DMRs with a maximum of 7 NAs (insufficient coverage) out of 73 samples were retained. For the CG methylation context, this resulted in 1569 DMRs (Additional file 16: Dataset S13), which were used for intersecting with dACRs and DEGs.

### Intersections between DMRs, transposable elements, and Non-TE genes

DMR positions were intersected with positions of TAIR10 transposable elements and non-TE genes (TAIR10 genes that are not associated with the term “transposable element gene”) using *bedtools intersect*, with a minimum overlap of 1bp (Additional file 2: Table S2).

### Nuclei isolation for ATAC-seq

For ATAC-seq analyses, each of the biological replicates was processed individually. Fixed tissue was chopped finely with 500 μl of General Purpose buffer (GPB; 0.5 mM spermine•4HCl, 30 mM sodium citrate, 20 mM MOPS, 80 mM KCl, 20 mM NaCl, pH 7.0, and sterile filtered with a 0.2-μm filter, followed by the addition of 0.5% of Triton-X-100 before usage). The slurry was filtered through one-layered Miracloth (pore size: 22-25 μm), followed by filtration twice through a cell-strainer (pore size: 40 μm) to collect nuclei.

### Fluorescence-activated cell sorting (FACS) for ATAC-seq

Liberated nuclei were sorted with a MoFlo XDP (Beckman Coulter) instrument outfitted with a 488-nm (elliptical focus, 100 mW) and a 375-nm (spherical focus, 35 mW) laser for scatter and DAPI emission, respectively. Nuclei were sorted with a 70-μm cytonozzle, sheath PBS [pH 7.0] at psi 30.5/30.0 sample/sheath, purify 1 drop, triggered off the DAPI emission (465/30nm). The 2C endoreduplicated population was identified as the first clear DAPI emitting population over scatter debris. DAPI emission was utilized to reduce further contaminating debris, followed by a clean-up utilizing 530/34 emission from the 488-nm laser. 488-nm channels: SSC (488/6), FL1 (520/34). 375-nm channels: FL8 (405/30), FL9 (465/30), FL10 (542/27). See Additional file 5: Fig. S31 for the gating scheme. Approximately 20,000 DAPI-stained nuclei were sorted using fluorescence-activated cell sorting (FACS) for each of two technical replicates. For samples from dwarfed mutant lines where leaf tissue was scarce, approximately 8000 nuclei were sorted per technical replicate.

### ATAC-seq library prep

Sorted nuclei were heated at 60°C for 5 min, followed by centrifugation at 4°C (1000*g*, 5 min). The supernatant was removed, and nuclei were resuspended with a transposition mix (1 μl homemade Tn5 transposase, 4 μl of 5X-TAPS-DMF buffer, and 15 μl autoclaved water) followed by a 37°C treatment for 30 min. Two hundred microliters SDS buffer and 8 μl 5 M NaCl were added to the reaction mixture, followed by 65°C treatment overnight. Nuclear fragments were then cleaned up using Zymo PCR column-purification (DNA Clean and Concentrator). Two microliters of eluted DNA was subjected to 14 PCR cycles, incorporating Illumina indices, followed by a 1.8:1 ratio clean-up using SPRI beads.

Genomic DNA libraries (10 ng input from the DNA extracts used for BS-seq-library prep) were prepared using a similar library prep protocol starting with Tn5 enzymatic digestion (0.5 μl homemade Tn5 transposase, 4 μl of 5X-TAPS-DMF buffer, and autoclaved water made up to a final reaction volume of 20 μl including the DNA template). Digested gDNA was immediately column-purified, followed by PCR (2 μl of eluted DNA was used as template for 11 PCR cycles) incorporating Illumina indices, followed by a 1.6:1 ratio clean-up using SPRI beads.

### Processing of ATAC-seq libraries and peak calling

Libraries were sequenced on an Illumina HiSeq3000 instrument with 2 × 150bp paired-end reads. Each technical replicate derived from nuclei sorting was sequenced at approximately 7 million paired-end reads per library. The reads were aligned as two single-end files to the TAIR10 reference genome using *bowtie2* [default options], filtered for the SAM flags 0 and 16 (only reads mapped uniquely to the forward and reverse strands), converted separately to bam files. The bam files were then merged and sorted, and PCR duplicates were removed using *picardtools.* The sorted bam files were then merged with the corresponding sorted bam file of a second technical replicate (samtools merge --default options) to obtain a final average of 11 million mapped reads for each biological replicate (Additional file 19: Dataset S15). Genomic DNA libraries were similarly aligned, with an average of 4.5 million mapped reads per library (Additional file 20: Dataset S16). Peak calling was carried out for each biological replicate using *MACS2* (Additional file 19: Dataset S15) using the following parameters:

*macs2 callpeak -t [ATACseqlibrary].bam -f BAM --nomodel --extsize 147 --keep-dup=all -g 1.35e8 -n [Output_Peaks] -B -q 0.01*

After peak calling, every peak set was further filtered based on their respective *q*-values in the MACS2 peaks.xls files, retaining peaks with *q* ≤ 0.001, thereby reducing the false positives when all 158 samples were subsequently tested together. This additional filtering step was carried out separately after MACS2 calling to minimize the effect on peak size based on the *q*-value.

Filtered peak files and .bam alignment files from a total of 158 samples (104 mutant samples plus 54 wild-type samples) were processed with the R package DiffBind to identify consensus peaks which overlapped in at least two out of three biological replicates per group, and represented peaks unique to at least one group (FDR adjusted *p*-value <0.01). To normalize peak accessibility counts with the background probability of Tn5 integration biases in the genome, .bam files of the control gDNA libraries were also provided in the DiffBind sample sheet (thereby ensuring that peak accessibility counts were normalized to controls). Further details for the DiffBind commands used are provided in the Additional file 21: Extended Methods.

A total of 35,049 consensus peaks were identified, with accessibility scores in each peak per sample evaluated in counts per million (CPM) after TMM (trimmed mean of M-values) normalization. Except for three out of 158 ATAC-seq libraries, FRIP (frequency of reads in peaks) scores relative to the consensus peak set was between 0.2 and 0.31 for all samples, reflecting the average representation of sample-specific peaks in the consensus dataset. After removing peaks which occurred in chloroplast and mitochondrial genomes, 34,993 peaks remained. These peaks were further processed to identify differentially accessible chromatin regions (dACRs, Additional file 12: Dataset S9) and highly variable dACRs (HV-dACRs, Additional file 13: Dataset S10) as explained below.

### Metaplot generation

For the 34,993 consensus ATAC-peaks (ACRs) identified from all 158 samples (representing all accessible regions throughout the genome), we first obtained accessibility values for each peak region. In a separate step, the final DiffBind object was used to identify peak summits for each of the 158 samples across every peak region (commands provided in Additional file 21: Extended Methods).

For every sample, a bed file with consensus peak coordinates, the position 100 bp upstream and downstream of the peak summit, and the mean accessibility value (for the entire peak region) were used to generate a bigWig file (.bw) using the *bedGraphtobigWig* command (UCSC software). Similarly, for cytosines in all contexts and their corresponding positions, methylation levels were derived for each Bisulfite library, and converted to bigWig files.

Metaplots were generated using the deepTools (3.5.0) package (https://deeptools.readthedocs.io/en/develop/index.html), first with the *computeMatrix* function to evaluate the mean value of the epigenetic factor tested (methylation in % or chromatin accessibility in CPM) across 10 bp non-overlapping bins, within 1000 bp upstream and downstream of a given set of reference regions (TAIR10 Transposable elements/TAIR10 Non-TE protein-coding genes). The output bed file from this command was subsequently used to generate metaplots using the *plotProfile* function.

### RNA extraction and RNA-seq library prep

RNA from each biological replicate was extracted individually using a column-based protocol adapted from [74]. RNA quality was validated with the Nanodrop spectrophotometer and normalized to 500 ng in a 50 μl volume. Normalized RNA was subsequently used for mRNA library prep using an in-house custom protocol adapted from Illumina’s TruSeq library prep, with details provided in [75].

### Mapping and identification of DEGs

RNA-seq libraries were sequenced at an average coverage of 8 million 150 bp single-end reads per library using HiSeq3000. Reads of the same sample from multiple sequencing lanes of the same flow cell were merged together, and 9 samples with > 12.5 million total reads were subsampled (using different seeds) to 80% using seqtk (v.2.0-r82-dirty, https://github.com/lh3/seqtk) with the following command:

*seqtk sample -sX <merged_fastq> 0.80* *> subsampled_output.fastq*

All samples were aligned using *bowtie2* to the TAIR10 reference genome, prepared using the *rsem-prepare-reference* function of the RSEM software. Aligned bam files were sorted and indexed using *samtools* V1.9 (mapping statistics in Additional file 22: Dataset S17). Gene transcript counts for each sample were estimated using *rsem-calculate-expression*. From each sample, chloroplast genes, mitochondrial genes, and rDNA cluster genes were excluded from downstream analyses. Twelve genes with excessive read counts across all samples were also excluded (Additional file 23: Dataset S18).

Transcript counts per sample and corresponding metadata were then imported using the R packages “tximport” and “tximportData” for creating a DESeq object (R package “DESeq2”).

#### Identification of DEGs (differentially expressed genes) between 18 accessions for *met1* mutants and wildtypes

Two DESeq objects containing 104 samples of *met1* mutants and 54 samples of wildtypes were generated separately. After filtering genes with low read counts, a common set of 19,473 genes were retained in both objects, and the DESeq function was applied under a one-factor model (~Accession) and default parameters (nbinomWald test). DEGs for each group (*met1* mutants or wildtypes) were identified from the DESeq output as those genes with a *p*-value < 0.01 and |log_2_ FoldChange| >1 (Additional file 3: Dataset S1, Additional file 4: Dataset S2).

#### Identification of DEGs between *met1* mutants and wildtypes

One DESeq object containing 158 samples (104 *met1* mutants and 54 wildtypes) was created and filtered for genes with low total read counts, resulting in a set of 21,657 genes to be analyzed. The DESeq function was applied to the object under the two-factor interaction model ~*genotype + accession + genotype:accession* (where *genotype ==* wild-type *or mutant*) using default parameters (nbinomWald test). To obtain DEGs between wild-type and mutant genotypes across all accessions (Additional file 6: Dataset S3), a contrast was performed set to *genotype*, retaining only those genes with a *p*-value< 0.01 and |log_2_ FoldChange| >1. For identifying accession-specific DEGs (Additional file 6: Dataset S3), similar contrasts were performed, nested within each accession. DEGs common to all 19 contrasts (18 accession-specific contrasts and the all-mutants-vs-all-wildtypes contrast) were identified as universal DEGs (Additional file 8: Dataset S5).

DEGs from each contrast were further classified as TE-DEGs or Non-TE-DEGs based on the TAIR10 gene annotation. Genes annotated with the term “transposable element gene,” as described in https://www.arabidopsis.org/portals/genAnnotation/gene_structural_annotation/annotation_data.jsp, were called “TE genes” in our analyses. All remaining 29,699 protein-coding genes were called “Non-TE genes.”

### Weighted gene co-expression network analysis

Transformed and normalized RNA-seq counts (vsd counts) were extracted for 10,151 unique Non-TE-DEGs identified from all 19 contrasts, across 158 samples. This matrix was then used to generate a weighted gene co-expression network using the R package WGCNA [76]. A soft threshold power of 4 was chosen and subsequently used for constructing the TOM (topological overlap matrix) with minModuleSize set to 30 and mergeCutHeight set to 0.25. This resulted in the generation of 9 distinct gene modules. Module eigengenes were examined for correlation to the sample genotype (1 for mutant and 0 for wild-type). The highest eigengene significance (0.9) was observed for Module “D” with 814 genes (Additional file 7: Dataset S4).

### Generation of consensus datasets

#### Consensus DEGs

These were generated by including DEGs from a total of 19 contrasts—18 pairwise contrasts (*met1* mutants vs wild-type plants) in each accession, and a contrast between all *met1* mutants and all wild-type plants. This set of DEGs was filtered to retain only DEGs that occurred in at least 2 out of the total 19 contrasts, to obtain a final set of 7132 consensus DEGs. These were further classified as 1401 TE-DEGs (Additional file 14: Dataset S11) and 5731 Non-TE-DEGs (Additional file 15: Dataset S12). The metric for evaluating expression levels in DEGs was chosen as the variance stabilized transformed read counts (vsd counts) generated by the DESeq2 package for each of the 158 RNA-seq libraries. We refer to these as transformed read counts in all figures.

#### HV-dACRs

ATAC-peaks found across all samples (34,993) were also filtered in several steps. First, peaks that showed similar accessibility between both mutant lines relative to the WT line in each accession were retained. The second round of filtering retained peaks that showed an accessibility change between mutants and wild-type plants in at least two out of 18 accessions. This resulted in 31,295 filtered consensus ATAC-peaks, which we refer to as differential ACRs (dACRs) (Additional file 12: Dataset S9). To identify highly variable dACRs (HV-dACRs), the following steps were carried out:

1. For every dACR, the coefficient of variation (CV) of mean accessibility levels between homozygous mutant samples and wildtypes was identified. From a distribution of CV values across all dACRs, only those among the top 25% were retained.
2. For every dACR, the coefficient of variation (CV) of accessibility levels across samples (acessions*genotype) was identified. From a distribution of CV values across all dACRs, only those among the top 25% were retained.

A union set of peaks from steps (1) and (2) resulted in 9505 highly variable dACRs (HV-dACRs, Additional file 13: Dataset S10), which we used for all visualizations and downstream processing. Chromatin accessibility levels were measured in counts per million (CPM) after TMM (trimmed mean of M-values) normalization generated by the DiffBind package. *k*-means clustering of HV-dACRs was carried out using the functions *kmeans()* in the R *stats* package, with *k* = 3.

#### Consensus DMRs

From the complete set of context-specific DMRs identified, DMRs with a maximum of 7 NA values (insufficient coverage) out of 73 samples were retained. This resulted in 1569 CG-DMRs, 207 CHG-DMRs, and 614 CHH-DMRs. Since we were primarily interested in understanding the effects of MET1 on genome-wide methylation changes, we considered only the 1569 CG-DMRs (Additional file 16: Dataset S13) for intersecting with other features. The methylation level for each CG-DMR was measured as arithmetic mean over methylation percentage of all sample-specific CG cytosines within the assigned chromosomal region.

### Generation of feature intersections between DEGs, DMRs, and HV-dACRs

#### DMR–DEG intersections

DMRs occurring at the extended gene body (between 100bp upstream of the TSS and 100bp downstream of the TTS of a gene) were called “gene body DMRs,” while those that occurred within 1.5kb upstream or downstream of the TSS/TTS respectively were named “cis DMRs.” Several DEGs had multiple DMRs associated with them, and therefore, we retained only one DMR for each DEG, which showed the largest difference in methylation level between mutant and WT for each mutant genotype, thereby aiming to represent only the strongest methylation signals that could explain gene expression differences. To determine the extent at which MET1-induced CG methylation could influence gene expression and compare it with methylation in all contexts, we intersected DEGs (TE-DEGs and Non-TE-DEGs separately) with CG-DMRs. For each DEG, we measured changes in gene expression levels between *met1* mutants and wild-type plants, and corresponding changes in methylation levels of their closest DMRs. For generating scatter plots, we divided wild-type methylation/expression levels of all examined genes into quintiles and colored points based on these quintiles.

#### HV-dACR–DEG intersections

Similarly, we analyzed the HV-dACRs closest to each DEG. Since a large majority of HV-dACRs occurred in proximity to the transcription start site, we grouped all HV-dACRs occurring either over the gene body or within 1.5 kb upstream or downstream of the TSS/TTS respectively, under a single “cis” category. For each DEG, a single HV-dACR which showed the largest difference in accessibility between mutant and WT for each mutant genotype was retained. We next measured changes in gene expression levels between *met1* mutants and wild-type plants, and corresponding changes in accessibility levels of their *cis* HV-dACRs. For generating scatter plots, we divided wild-type accessibility/expression levels of all examined genes into quintiles and colored points based on these quintiles.

#### Intersections of DMRs and HV-dACRs with Non-DEG genes

As a control for the DEGs, we generated similar feature intersections for Non-DEGs as well. From a total of 1678 TE genes and 19,979 Non-TE genes analyzed using DESeq2, we identified 277 TE genes and 14,248 Non-TE genes which were not classified as “consensus DEGs,” and we subsequently referred to these as “Non-DEGs.” To ensure that the number of Non-DEGs analyzed were comparable to the number of DEGs in each category, 5731 Non-TE genes were randomly subsampled from the total set of 14,248 Non-TE (Non-DEG) genes. However, only 250 TE genes were randomly subsampled from the total set of 277 TE (Non-DEG) genes, since the total number of TE-DEGs (1401) exceeded the number of TE (Non-DEG) genes.

#### DMR–HV-dACR–DEG intersections

For simplicity, the above class of three-way intersections was only carried out for CG-DMRs and Non-TE genes. In short, Non-TE-DEGs carrying CG-DMRs in both the extended gene body and in *cis* were combined together. These combined DEGs were then filtered to identify only those which carried a HV-dACR in *cis*. This resulted in a final set of 164 DEGs which carried both a CG-DMR and a HV-dACR in *cis*. Similarly, when a control set of Non-TE Non-DEG genes were used to generate similar intersections, a final set of 107 Non-DEGs carrying both a CG-DMR and a HV-dACR in *cis* were identified.

### gbM-like and CG teM-like genes

To follow conventional definitions of gene body methylation (gbM), only Non-TE-DEGs with CG-DMRs overlapping the gene body were considered. These criteria were satisfied by 196 DEGs across homozygous *met1* mutants in 17 accessions (since one accession, Bl-1, did not have homozygotes). From this set of genes, we identified 91 gbM-like genes, which had >80% methylation in the wild-type parent, and transformed expression counts in wild-type ≥6.53, which represented moderate to high expression in wild-type samples (among the top three quintile ranges of the distribution of gene expression levels in wildtypes) for all Non-TE genes. In a separate analysis, 601 out of 19,979 Non-TE genes (comprising DEGs and Non-DEGs) were identified to carry CG-DMRs in their gene body. From these, 366 genes were found to be gene body methylated (using the criteria explained above) in at least one wildtype among 17 accessions. The number of accessions represented by every gene was then counted to identify 3 genes that were similarly gbM-like in wildtypes of all 17 accessions (see diagram in Additional file 5: Fig. S20b).

Next, we identified 51 CG teM-like genes that exhibited >80% CG methylation in the wild-type state, and transformed expression counts in wild-type state being ≤4.39, which represented genes in the lowest quintile range of the distribution of wild-type expression levels for all 196 genes.

Metadata (transformed expression counts and methylation levels in *met1* and mutants wild-type plants) for gbM-like and CG teM-like genes were then extracted from the total set of 196 genes (thereby representing the same genes in all accession backgrounds) and used for generating visual plots in Additional file 5: Fig. S20a and Additional file 5: Fig. S21.

### Gene Ontology enrichment and visualization

GO enrichment was carried out using agriGO (http://bioinfo.cau.edu.cn/agriGO), with the singular enrichment analysis (SEA) analysis tool and Arabidopsis (TAIR10) gene model as the reference. Graphical results of significant GO terms were generated in agriGO. The GO terms were further visualized with ReviGO (http://revigo.irb.hr).

### Segregation distortion analyses

#### Experimental design

To accurately estimate the extent of this segregation distortion in mutants of various accessions, we grew a maximum of 96 segregating progeny from heterozygous parent lines (2 mutant lines per accession) and genotyped them individually using amplicon-sequencing of the *MET1* locus, amplifying a 150-bp region around the CRISPR/Cas9-induced frameshift mutations.

#### Amplicon-seq library preparation, sequencing, and genotyping

Amplicon-seq libraries were prepared according to the CRISPR-finder system [77], where amplicons from multiple 96-well plates can be pooled together for high-throughput sequencing by incorporating frameshifted primers and TruSeq adapters with 96 barcodes. The amplicons were designed as 152-bp sequences spanning the gRNA target site in the *MET1* gene locus (Additional file 1: Table S1)*.* A total of 2788 individual samples were sequenced at an average coverage of 12,000 reads per sample on a HiSeq3000 instrument with 2 × 150 bp paired-end reads.

Sequenced read pairs were first merged using FLASH (Fast Length Adjustment of SHort reads) (https://ccb.jhu.edu/software/FLASH/), followed by demultiplexing based on plate-specific frameshifted primers (see Additional file 24: Table S4) using the *usearch10 fastx_truncate* function. Only samples with ≥80 reads were retained for downstream processing. For all samples within a plate (i.e., segregating progeny), amplicon reads per individual were counted for the ratio of wild-type alleles to mutant alleles, to estimate whether the genotypes were homozygous for the mutant allele (wild-type reads ≤ 15%), heterozygous (wild-type reads ≥ 42% or ≤ 58%), or wild-type (wild-type reads ≥ 90%). A fourth genotypic classification, “skewed heterozygous” was made for individuals where the read ratio between the wild-type and mutant alleles were either 0.15–0.42 or 0.58–0.90 (i.e., if either one of the alleles were more represented than the other, but not approximately equal in counts).

For Bu-0 Line 2, Ste-0 Line 2, and Bs-1 Line 2 samples that had more than one mutant *MET1* allele, additional genotypic categories were specified: homozygous allele 2, heterozygous allele 2, skewed heterozygous allele 2, bi-allelic, skewed bi-allelic and tri-allelic (Additional file 17: Table S3).

### Cytometric ploidy analysis

Cytometric determination of generative ploidy levels was conducted on a CytoFlex (Beckman Coulter) outfitted with a 488-nm laser, 10 μL min^−1^ flow rate. Nuclei were freshly liberated by chopping into cold General-purpose Buffer [78], filtered through 40-μm mesh, and stained with 50 μg mL^−1^ propidium iodide and 50 μg mL^−1^ RNase for 10 min at 20°C. The 2C endoreduplication population was identified as the first clear PI emitting population over scatter debris. The 2C nuclei of *Solanum lycopersicum* (var. Moneymaker) provided by the Zentrum für Molekularbiologie der Pflanzen (ZMBP) Cultivation Facility and *Capsicum annuum* provided by Annett Strauss (ZMBP) were used as internal standards to determine the relative Arabidopsis generative ploidy levels.

## Supplementary Information

**Additional file 1: Table S1.** (1) CRISPR guide-RNA design for *MET1* knockout, primers used for PCR-based genotyping of frameshift mutations. (2) List of accessions used in this study. (3) List of mutants in each accession and identified mutations.

**Additional file 2: Table S2.** (1) Numbers of DEGs, DMRs and HV-dACRs identified across all samples. (2) Numbers of DEGs identified across all contrasts. (3) Numbers of unique genes identified in intersections between DEGs, DMRs and HV-dACRs. (4) Intersections between DMR and HV-dACRs with TEs and genes.

**Additional file 3: Dataset S1.** List of DEGs identified within 18 accessions among WT samples.

**Additional file 4: Dataset S2.** List of DEGs identified within 18 accessions among *met1* mutant samples.

**Additional file 5: Supplementary Figures S1-S32**(with legends).

**Additional file 6: Dataset S3.** Names and coordinates of DEGs identified in 18 accession-specific contrasts and an all mutant vs all WT contrast.

**Additional file 7: Dataset S4.** List of 814 genes in module D (identified from gene network analysis).

**Additional file 8: Dataset S5.** Names and coordinates of DEGs universal across 19  mutant vs WT contrasts.

**Additional file 9: Dataset S6.** Positional coordinates of CG-DMRs.

**Additional file 10: Dataset S7.** Positional coordinates of CHG-DMRs.

**Additional file 11: Dataset S8.** Positional coordinates of CHH-DMRs.

**Additional file 12: Dataset S9.** Positional coordinates and TMM values of dACRs.

**Additional file 13: Dataset S10.** Positional coordinates and TMM values of highly variable (HV) dACRs.

**Additional file 14: Dataset S11.** vsd-transformed read counts for Consensus TE-DEGs across 158 samples (*met1* mutants and WTs).

**Additional file 15: Dataset S12.** vsd-transformed read counts for Consensus Non-TE-DEGs across 158 samples (*met1* mutants and WTs).

**Additional file 16: Dataset S13.** Methylation values and coordinates of subset of CG-DMRs with a maximum of 7 'NA' calls across 73 libraries.

**Additional file 17: Table S3.** Segregation distortion results for each mutant line.

**Additional file 18: Dataset S14.** Mapping efficiency, chloroplast read conversion efficiency of Bisulfite-seq libraries.

**Additional file 19: Dataset S15.** Mapping efficiency of ATAC-seq libraries, number of MACS2 peaks identified for each sample.

**Additional file 20: Dataset S16.** Mapping efficiency of genomic DNA libraries used as a control for ATAC-seq libraries.

**Additional file 21.** Extended Methods.

**Additional file 22. Dataset S17.** Mapping efficiency of RNA-seq libraries.

**Additional file 23: Dataset S18.** High count genes which were filtered out before DEG calling (in mutant vs WT contrasts).

**Additional file 24: Table S4.** (1) Frameshifted oligonucleotides (forward and reverse primers) used for amplicon-sequencing of multiple samples. (2) List of individual samples analyzed for each mutant line.

**Additional file 25: Dataset S19.** Sample metadata of ATAC-seq, Bisulfite-seq, RNA-seq and Amplicon-seq libraries uploaded on the ENA browser.

**Additional file 26:** Review history.

## Data Availability

Raw Data Availability: SRA links for raw sequencing data have been deposited at ENA (European Nucleotide Archive, https://www.ebi.ac.uk/ena/browser/home) under the accession numbers PRJEB53354 (RNA-seq data [79]), PRJEB54034 (ATAC-seq data [80]), PRJEB54036 (Bisulfite-seq data [70]), and PRJEB54071 (Amplicon-seq data [81]). Sample metadata for the uploaded files are provided in Additional file 25: Dataset S19.
