# Supplementary material for: Canalization of genome-wide transcriptional activity in Arabidopsis thaliana accessions by MET1-dependent CG methylation
Source: Genome Biol. 2022 Dec 20;23:263. doi: 10.1186/s13059-022-02833-5 (PMC9768921; doi:10.1186/s13059-022-02833-5)
Supplement: Supplementary file 5 — Additional file 5: Supplementary Figures S1-S32(with legends). [file 13059_2022_2833_MOESM5_ESM.pdf]

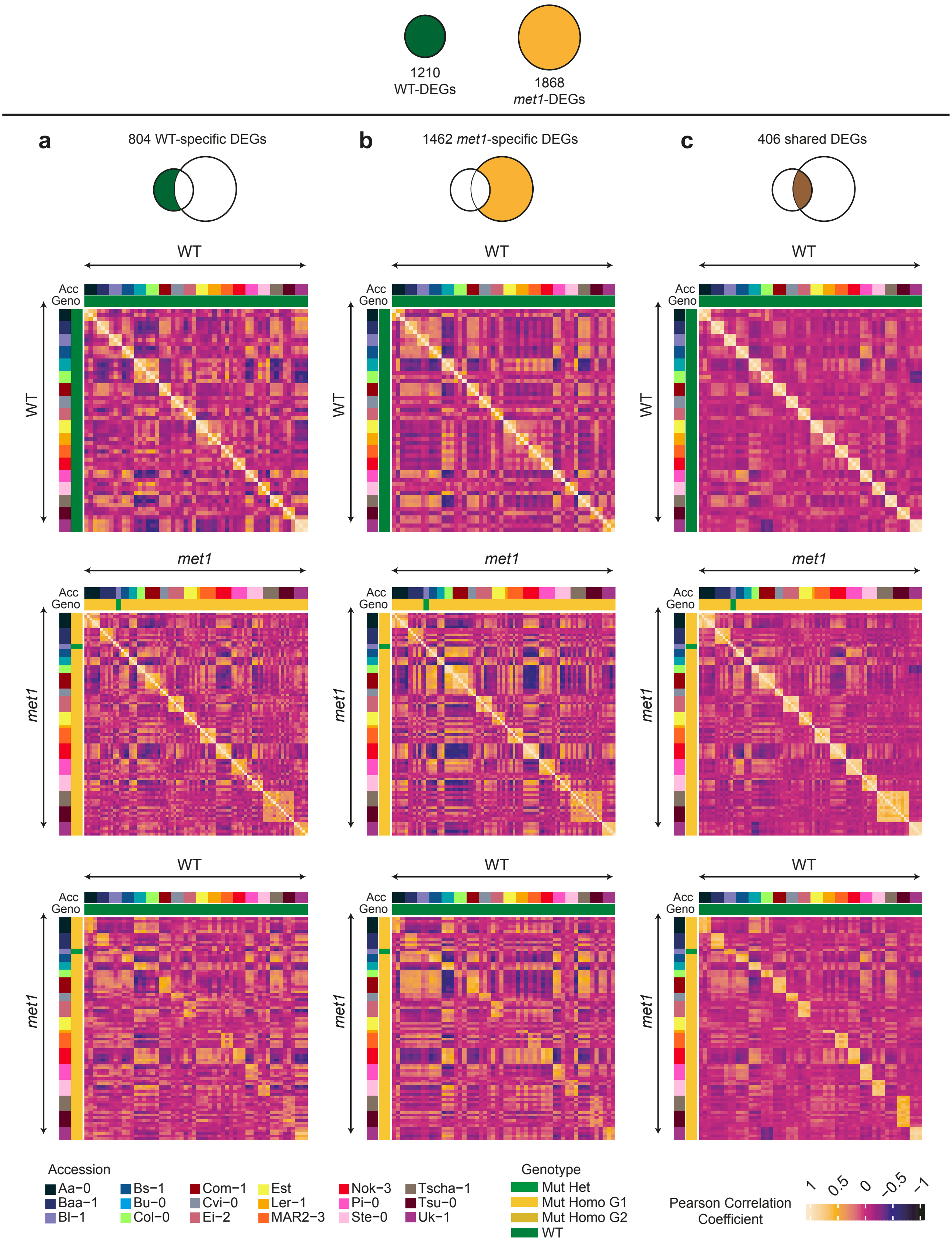

Figure S1. Transcriptome changes in met1 mutants are gene- and accession-specific. Pearson correlation heatmaps of gene expression levels between accessions in *met1* mutants and wildtypes, for DEGs specific to wildtypes (a), DEGs specific to *met1* mutants (b) and DEGs shared between *met1* mutants and wildtypes (c). WT, wild type; Mut Het, heterozygous *met1* mutants; Mut Homo G1, first-generation homozygous *met1* mutants; Mut Homo G2, second-generation homozygous *met1* mutants.

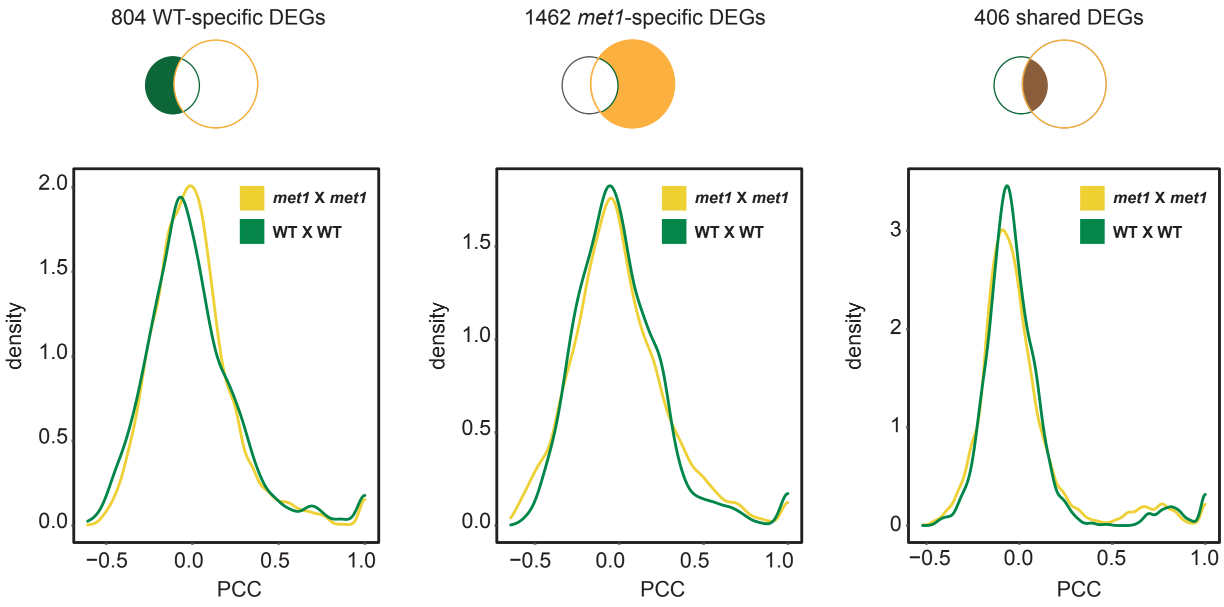

Figure S2. Density distributions of Pearson Correlation Coefficients among *met1* mutants of different accessions and wildtypes of different accessions respectively, for three different groups of DEGs.

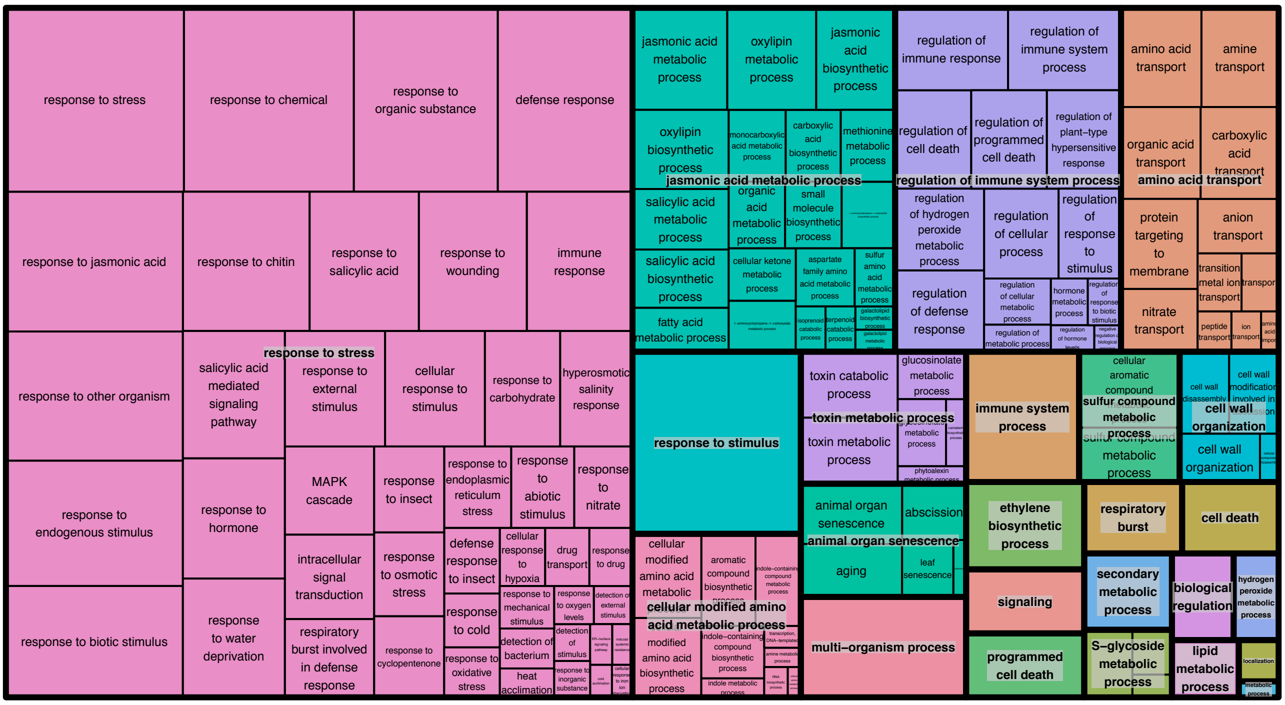

Figure S3. Gene Ontology enrichment for 2,013 Non-TE-DEGs identified in a contrast between all *met1* mutants and all wild-type samples.

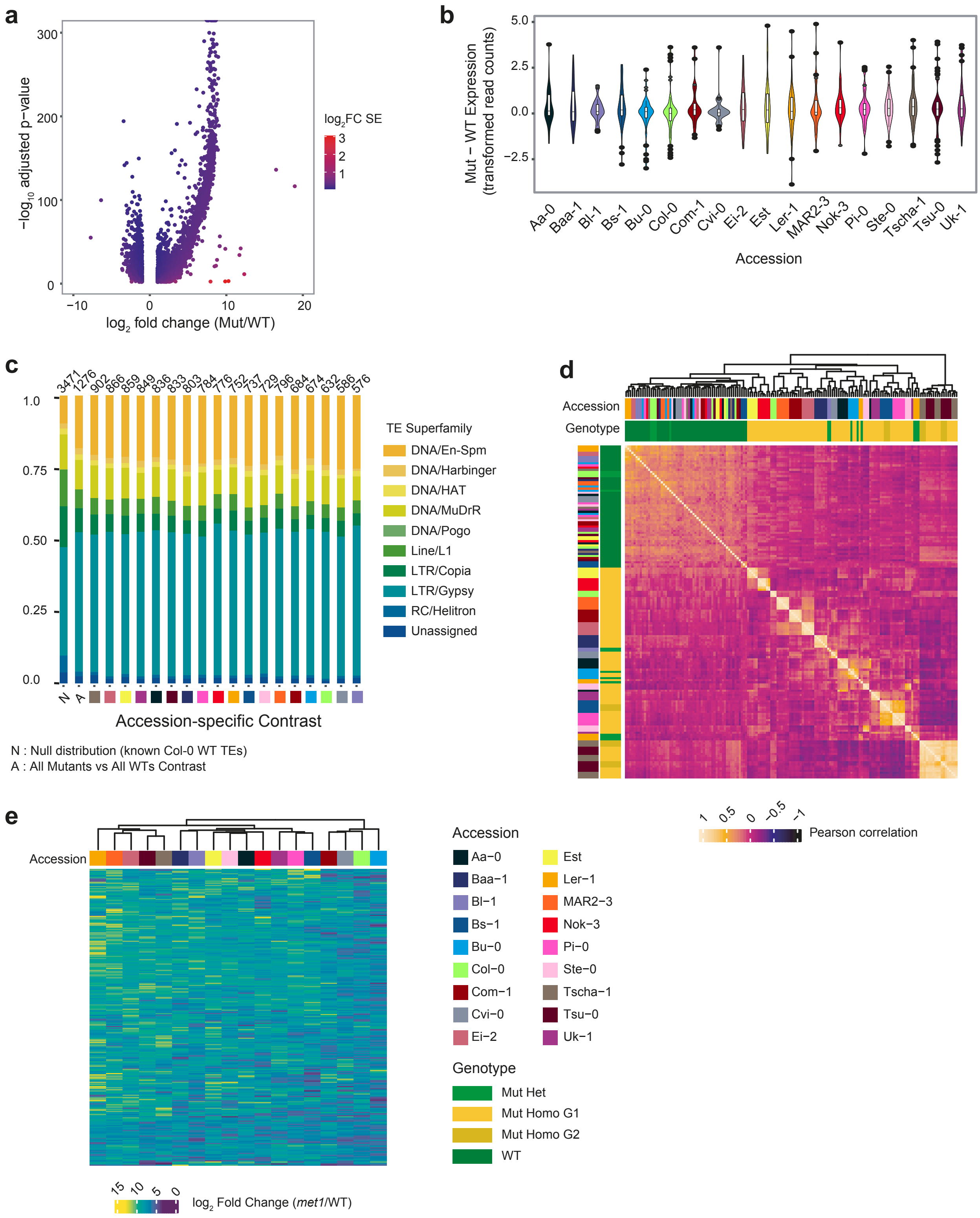

Figure S4. Accession-specific variation of differentially expressed genes (DEGs) in *met1* mutants. (a) Volcano plot colored by standard error (SE) of $\log_2$ fold change (FC) in all-mutants-against-all-wild-type DEGs. (b) Thirty random genes examined for accession-specific variation in expression changes (measured as transformed read counts). (c) Distribution of TE superfamilies in TE-DEGs across 19 contrasts, and a null distribution of all TE genes (denoted by 'N'). (d) Correlation between 291 universal DEGs across 158 RNA-seq libraries (104 *met1* mutant and 54 wild-type samples). (e) Heatmap showing $\log_2$ fold change in expression (Mut/WT) of 276 universal TE-DEGs. Color code of accessions in (b), (c) and (d) follows the same legend shown in (e). WT, wild type; Mut Het, heterozygous *met1* mutants; Mut Homo G1, first-generation homozygous *met1* mutants; Mut Homo G2, second-generation homozygous *met1* mutants.

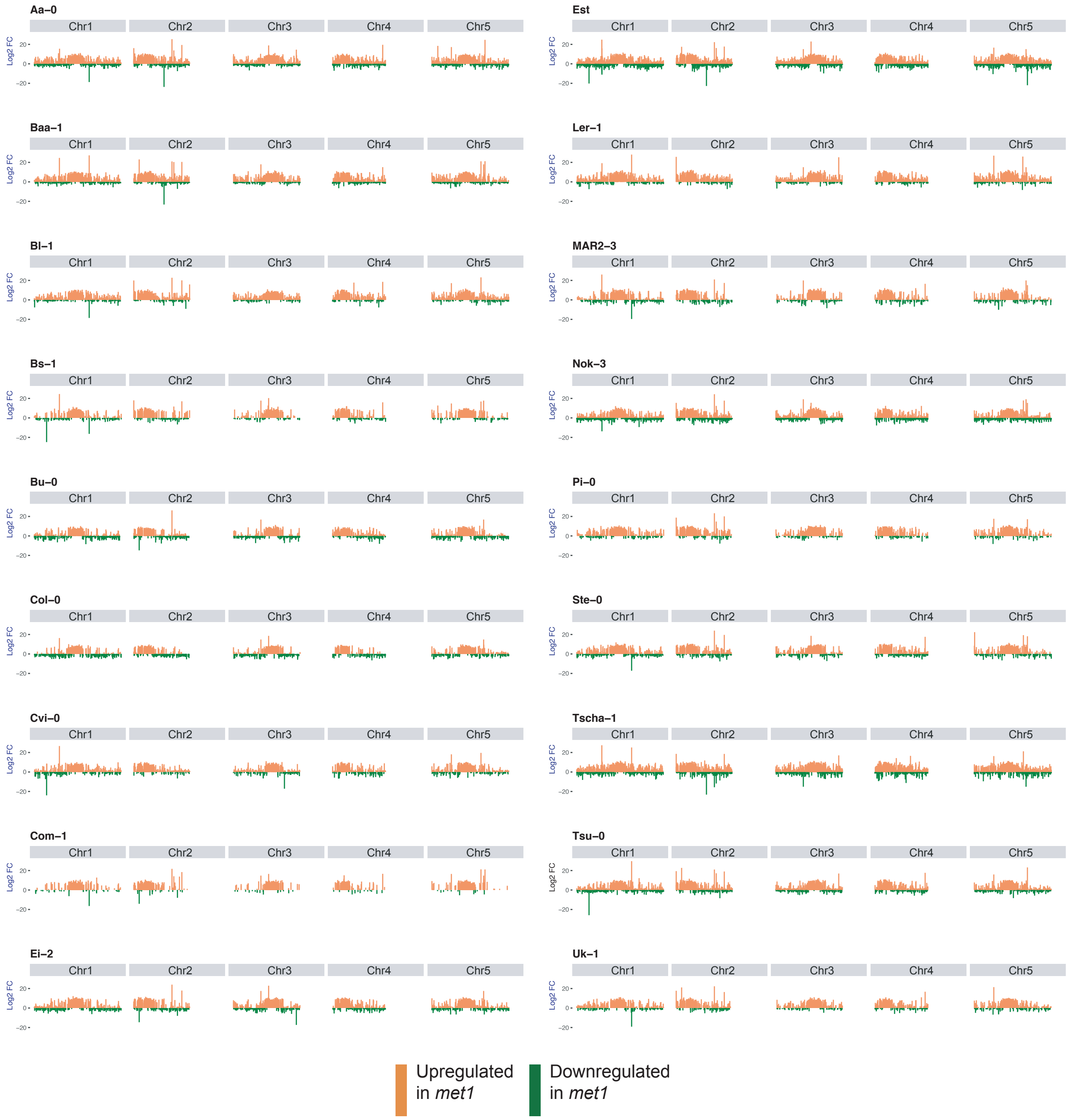

Figure S5. Accession-specific variation in the chromosomal distribution of up- and down-regulated DEGs.

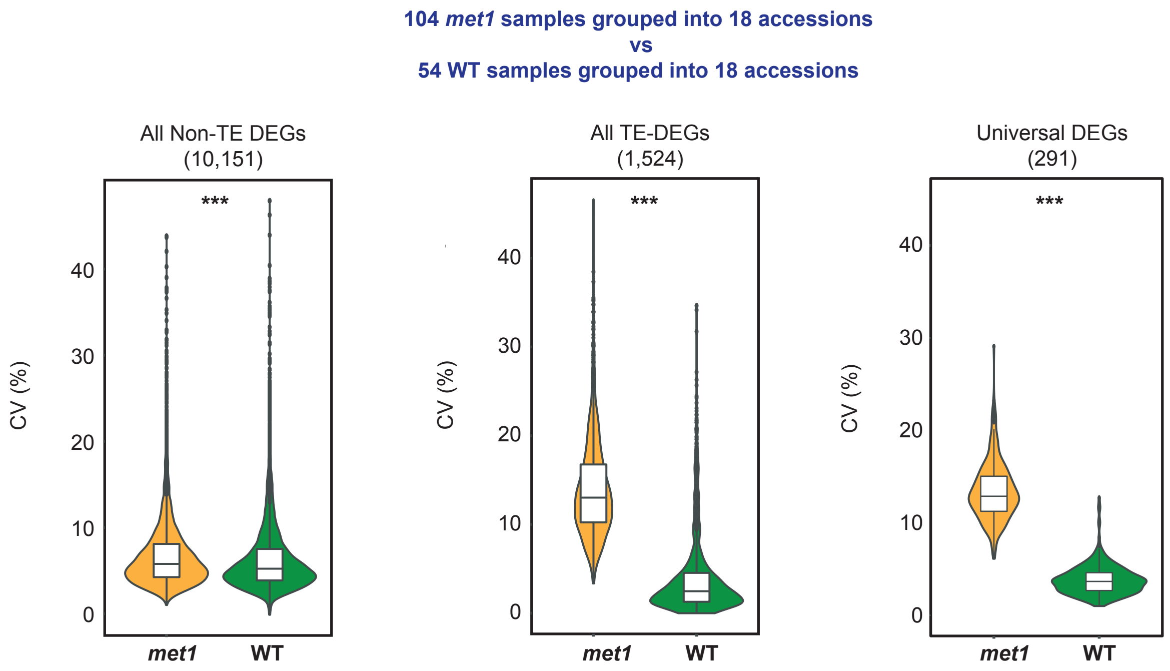

Figure S6. Boxplots showing distribution of the Coefficient of Variation (CV) for expression level (measured in transformed read counts) across accessions between *met1* mutants and wild-types (samples grouped by accession) at 10,151 Non-TE-DEGs, 1,524 TE-DEGs and 291 Universal DEGs. *** indicates Wilcoxon-test p-value <0.0001.

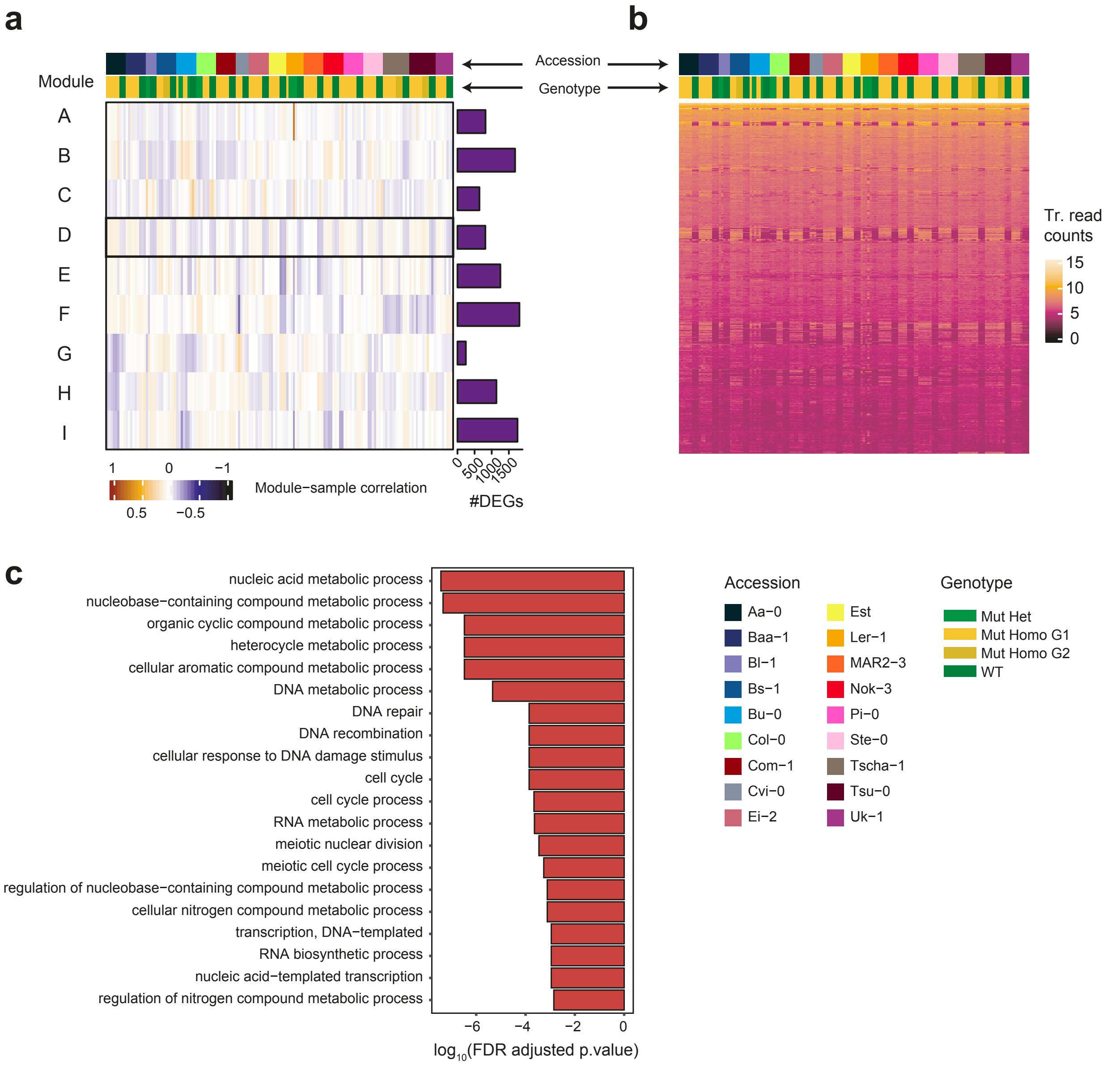

Figure S7. Gene network analysis on unique Non-TE-DEGs across all 19 contrasts. (a) Heatmap showing module-sample correlation levels of 10,151 unique Non-TE-DEGs from 19 contrasts, across 158 samples. The rows represent 9 modules (labeled A - I) based on weighted gene co-expression network analysis, with module 'D' highlighted due to its high association with sample genotype. The marginal barplots along the vertical axis indicate the number of genes in each module. (b) Heatmap of transformed read counts across 158 RNA-seq libraries for 814 genes in module 'D'. (c) Results of the top 20 most significant Gene Ontology enrichment terms for 814 genes in module 'D'. WT, wild type; Mut Het, heterozygous met1 mutants; Mut Homo G1, first-generation homozygous *met1* mutants; Mut Homo G2, second-generation homozygous *met1* mutants.

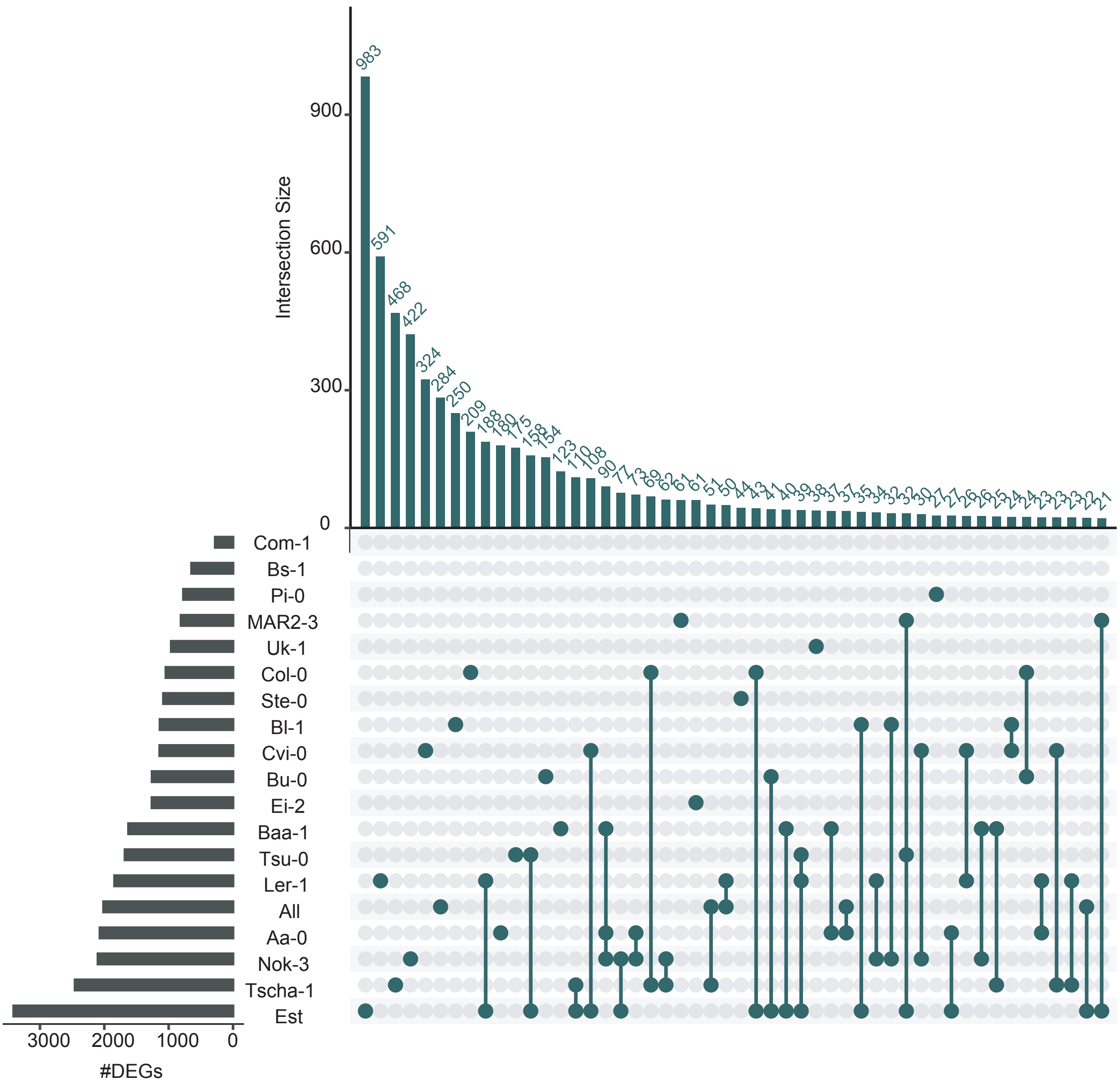

Figure S8. Comparisons and intersections between accession-specific DEGs. Upset plot of the top 50 all-pairwise intersections between Non-TE-DEGs in 19 accession-specific contrasts.

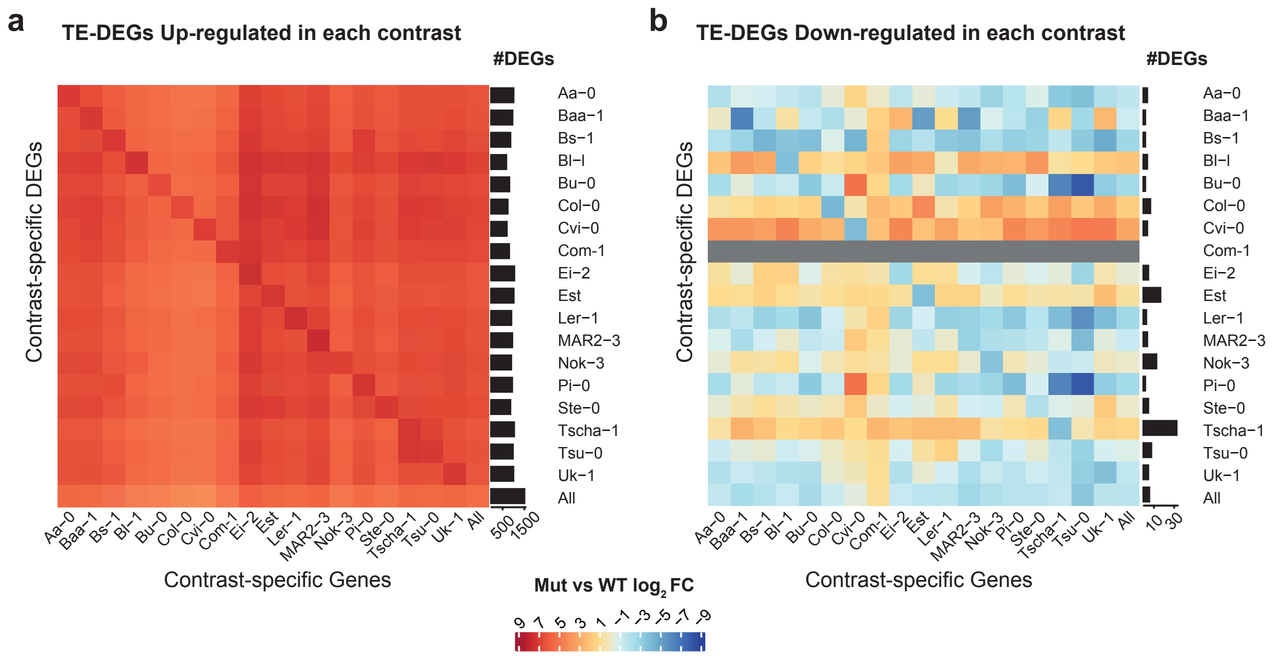

Figure S9. Quantitative comparisons between TE-DEGs across accession-specific contrasts. Heatmaps showing average $\log_2$ fold change (Mut/WT) for all TE-DEGs which are (e) upregulated and (f) downregulated in each accession-specific contrast, measured for the same genes across all other contrasts.Mut, *met1* mutant; WT, wildtype. Marginal barplots indicate the number of TE-DEGs in every accession-specific contrast. Com-1 did not exhibit any downregulated TE-DEGs and therefore the corresponding heatmap tiles in (b) are colored grey.

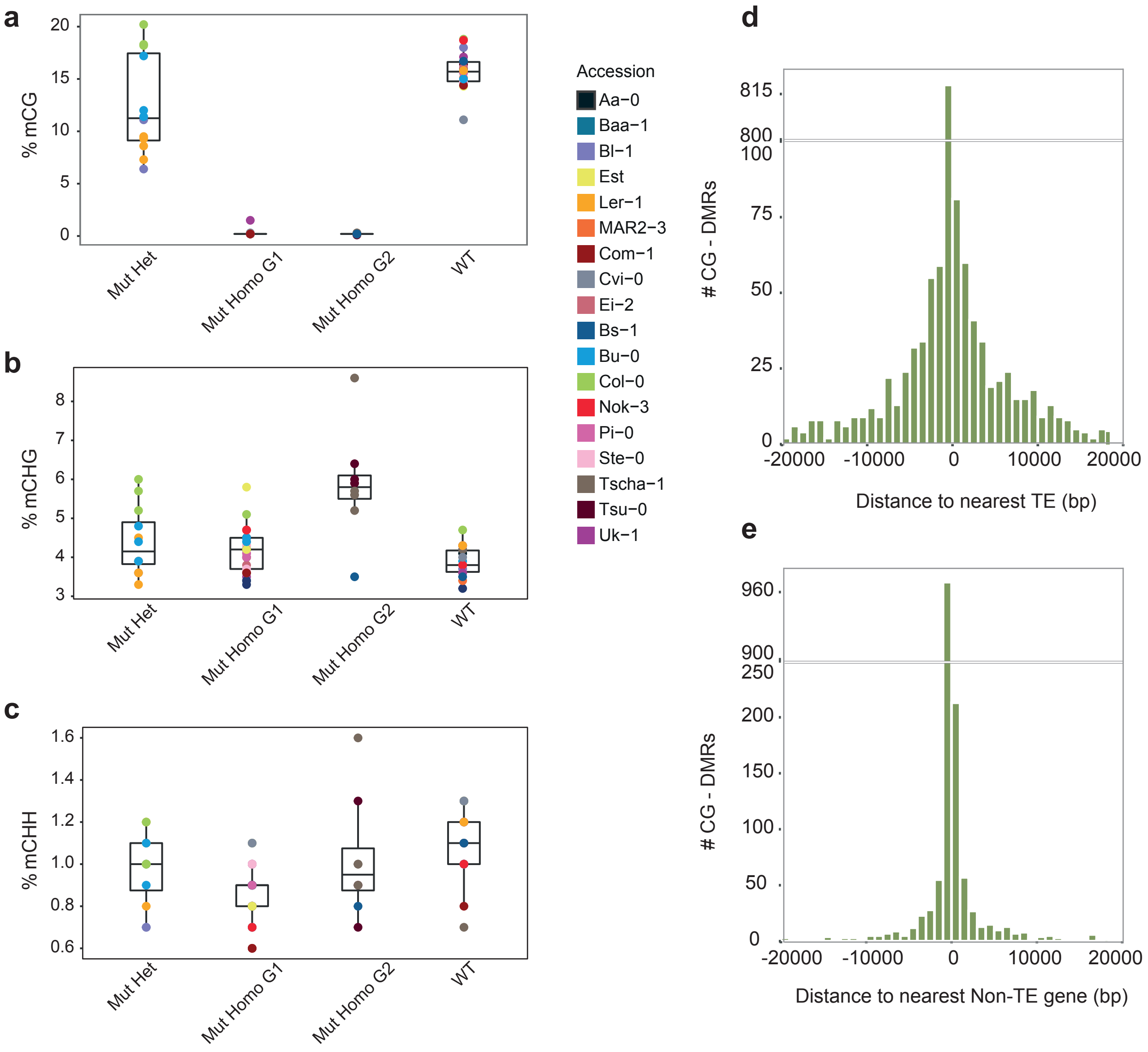

Figure S10. Altered genome-wide methylation levels in *met1* mutants compared to wild-type individuals. Genome-wide methylation levels of 73 samples (55 *met1* mutants and 18 wild-type plants) in the (a) CG, (b) CHG and (c) CHH contexts. Histogram showing distance of CG-DMRs to (d) nearest TE and (e) nearest Non-TE gene. WT, wild type; Mut Het, heterozygous *met1* mutants; Mut Homo G1, first-generation homozygous *met1* mutants; Mut Homo G2, second-generation homozygous *met1* mutants.

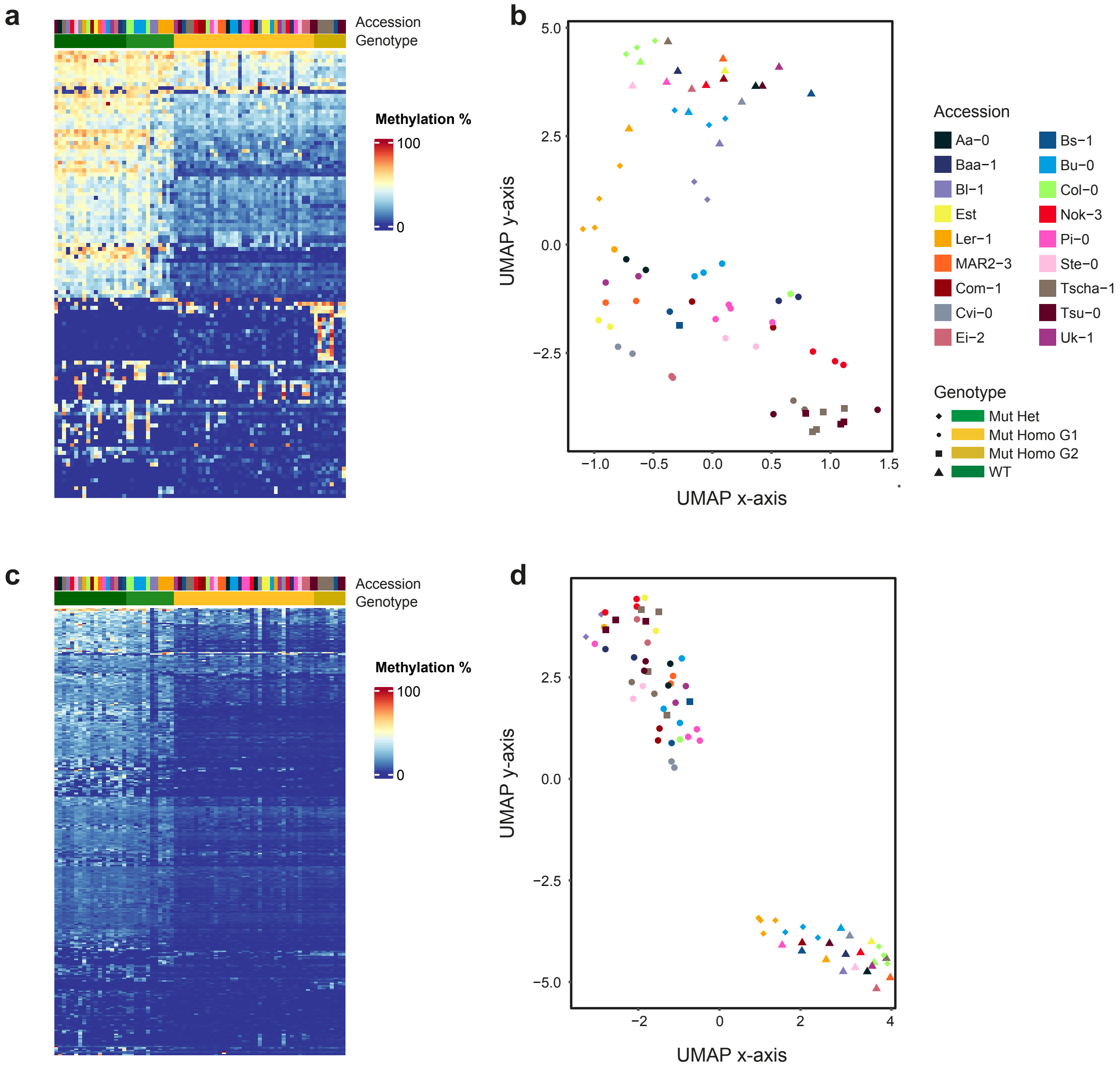

Figure S11. Differential methylation in non-CG contexts in *met1* mutants and wild-type individuals. (a) Heatmap and (b) UMAP visualization of CHG methylation levels in 73 samples (55 mutants and 18 wild-type plants) across 114 CHG-DMRs (from a total of 350 CHG-DMRs). (c) Heatmap and (d) UMAP visualization of CHH methylation levels in 73 samples (55 mutants and 18 wild-type plants) across 334 CHG-DMRs (from a total of 1,023 CHG-DMRs). WT, wild type; Mut Het, heterozygous *met1* mutants; Mut Homo G1, first-generation homozygous *met1* mutants; Mut Homo G2, second-generation homozygous *met1* mutants.

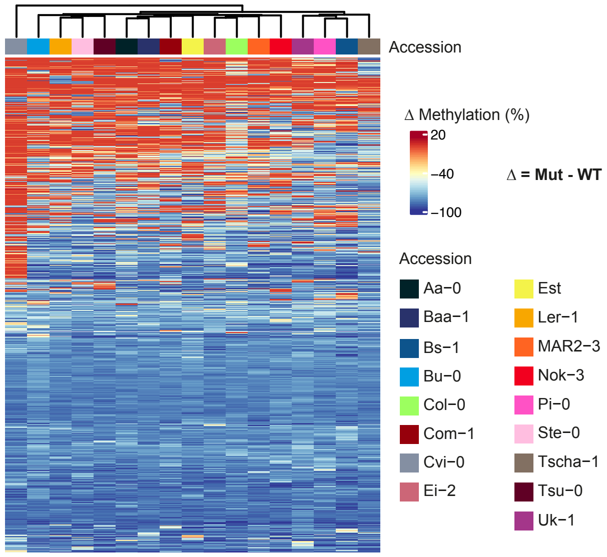

Figure S12. Heatmap of differences in CG methylation between first generation *met1* homozygotes and wild-type samples for 17 accessions across 749 CG-DMRs.

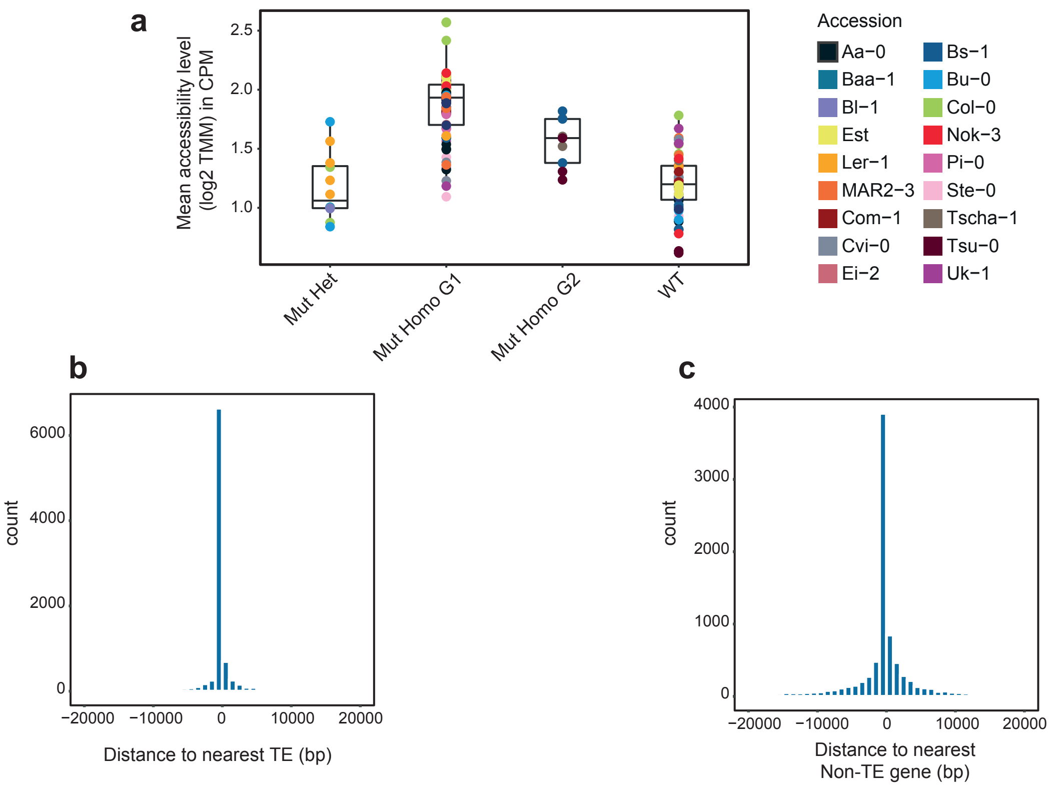

Figure S13. Altered genome-wide chromatin accessibility in *met1* mutants compared to wild-type individuals. (a) Boxplots showing mean chromatin accessibility levels across 9,505 HV-dACRs for various genotypes. (b) Histograms showing distance of HV-dACRs to nearest TE and (c) nearest Non-TE protein coding gene. WT, wild type; Mut Het, heterozygous *met1* mutants; Mut Homo G1, first-generation homozygous *met1* mutants; Mut Homo G2, second-generation homozygous *met1* mutants.

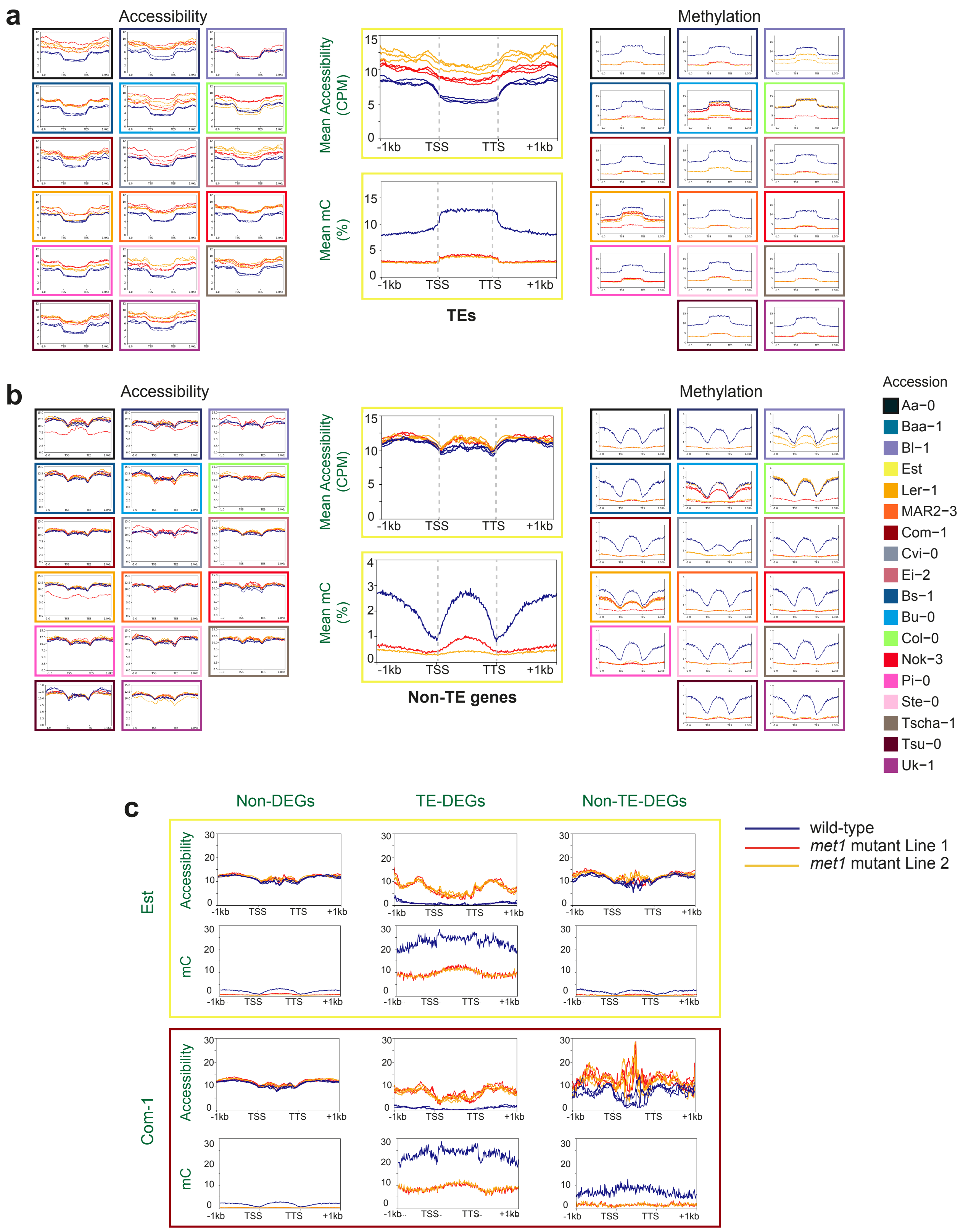

Figure S14. *met1* mutants have more accessible chromatin and are hypomethylated over TEs and genes. Metaplots of mean chromatin accessibility across all (a) TAIR10 TEs and (b) Non-TE protein-coding genes in 18 accessions. Boxes are color-coded by accession, and data are colored by genotype. (c) Metaplots of chromatin accessibility and methylation levels for Est and Com-1, across Non-DEGs, TE-DEGs and Non-TE-DEGs. TSS and TTS denote transcription start site and transcription termination site respectively. Methylation levels are represented as % cytosine methylation (all-contexts) and accessibility levels are represented as TMM normalized values in counts per million (CPM).

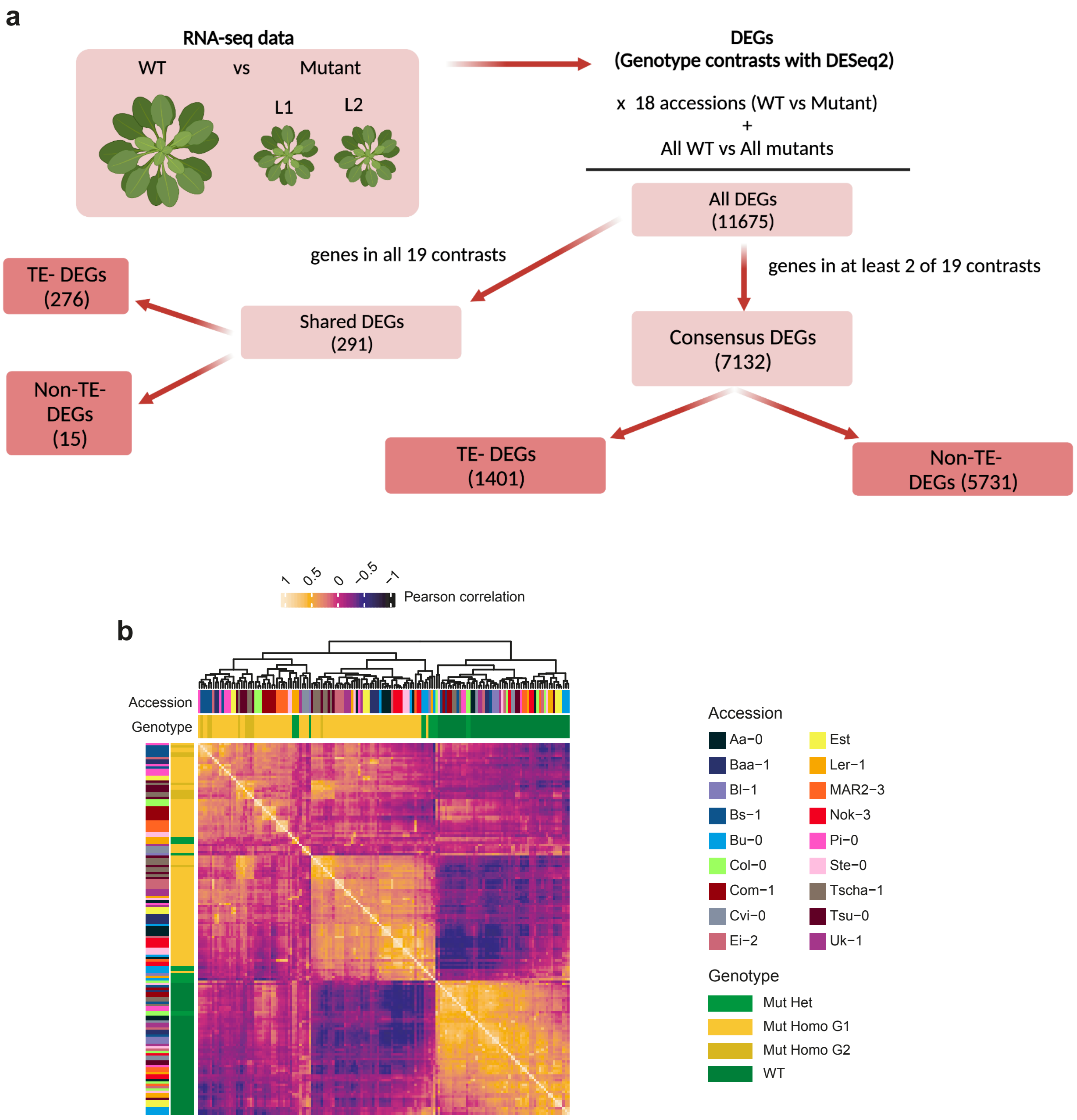

Figure S15. (a) Diagram of generating consensus DEGs from RNA-seq data. (b) Correlation between 7132 consensus DEGs across 158 RNA-seq libraries (104 *met1* mutant and 54 wild-type samples). Genotypes represented are wildtypes ('WT'), heterozygous *met1* mutants ('Mut Het'), first generation homozygous *met1* mutants ('Mut Homo G1') and second generation homozygous *met1* mutants ('Mut Homo G2').

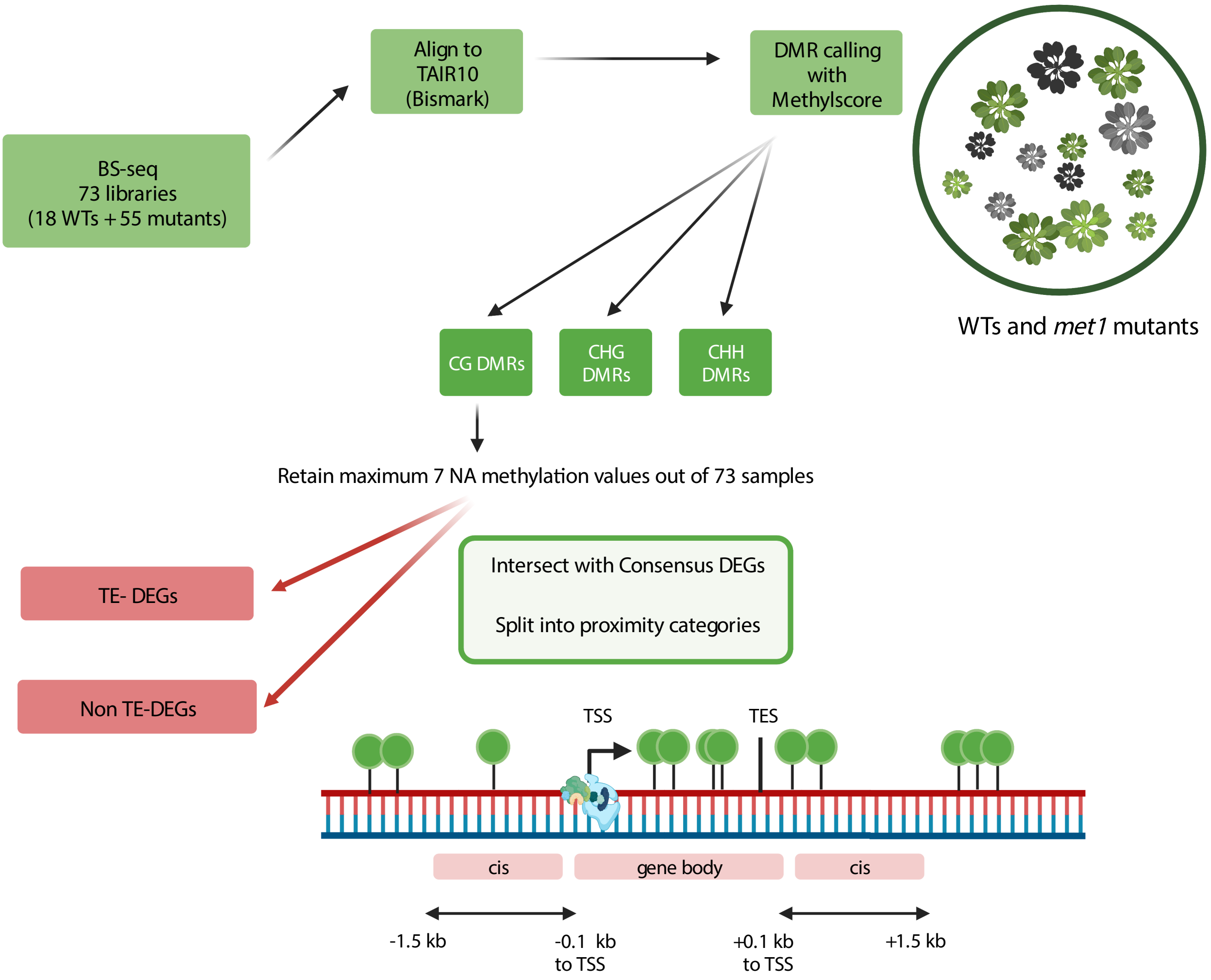

Figure 16. Diagram of generating DMRs from BS-seq data and intersections with consensus DEGs.

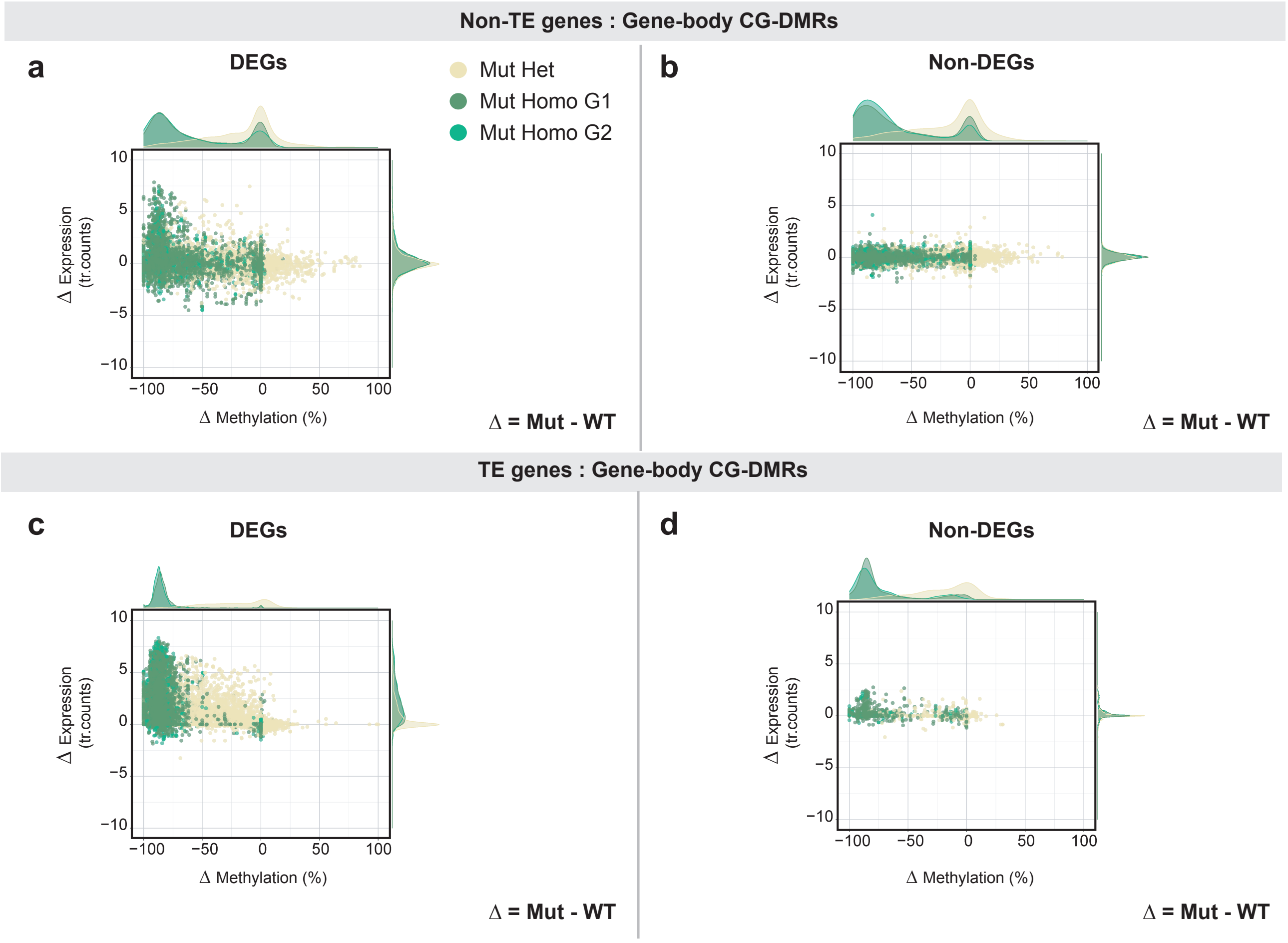

Figure S17. CG-DMRs in gene-bodies of Non-TE genes (a-b) and TE genes (c-d). Scatter plots showing differences in CG methylation between *met1* mutants and wild-type plants against differences in gene expression. Dots in the scatter plot are colored by genotype of *met1* mutants; wildtypes ('WT'), heterozygous met1 mutants ('Mut Het'), first generation homozygous *met1* mutants ('Mut Homo G1') and second generation homozygous *met1* mutants ('Mut Homo G2') with x- and y-axis density distributions of each genotype. Expression levels are represented as transformed read counts (Methods) and methylation levels are represented as % CG methylation.

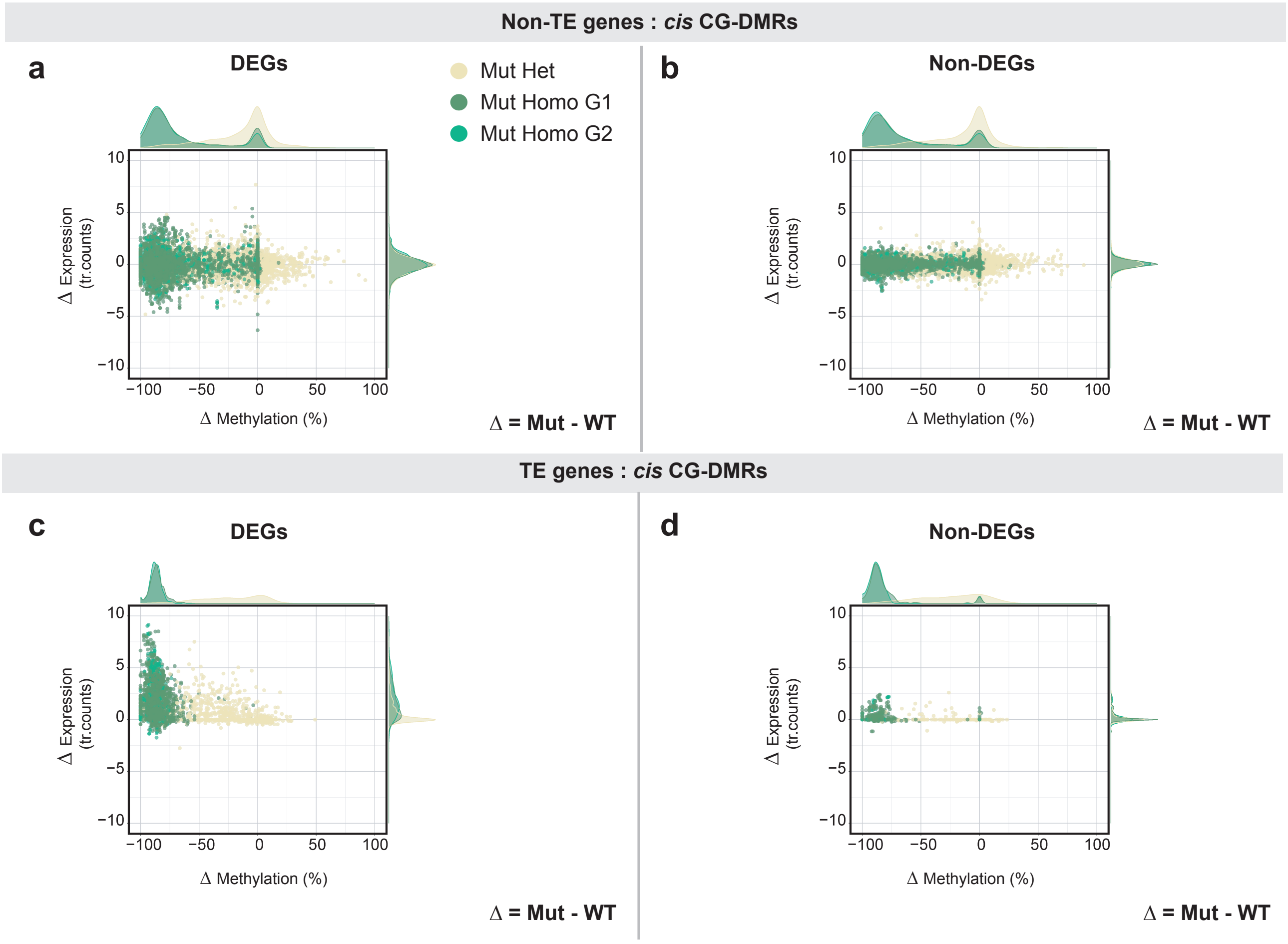

Figure S18. CG-DMRs in cis to Non-TE genes (a-b) and TE genes (c-d). Scatter plots showing differences in CG methylation between met1 mutants and wild type plants against differences in gene expression. Dots in the scatter plot are colored by genotype of *met1* mutants; wildtypes ('WT'), heterozygous *met1* mutants ('Mut Het'), first generation homozygous*met1* mutants ('Mut Homo G1') and second generation homozygous *met1* mutants ('Mut Homo G2') with x- and y-axis density distributions of each genotype. Expression levels are represented as transformed read counts and methylation levels are represented as % CG methylation.

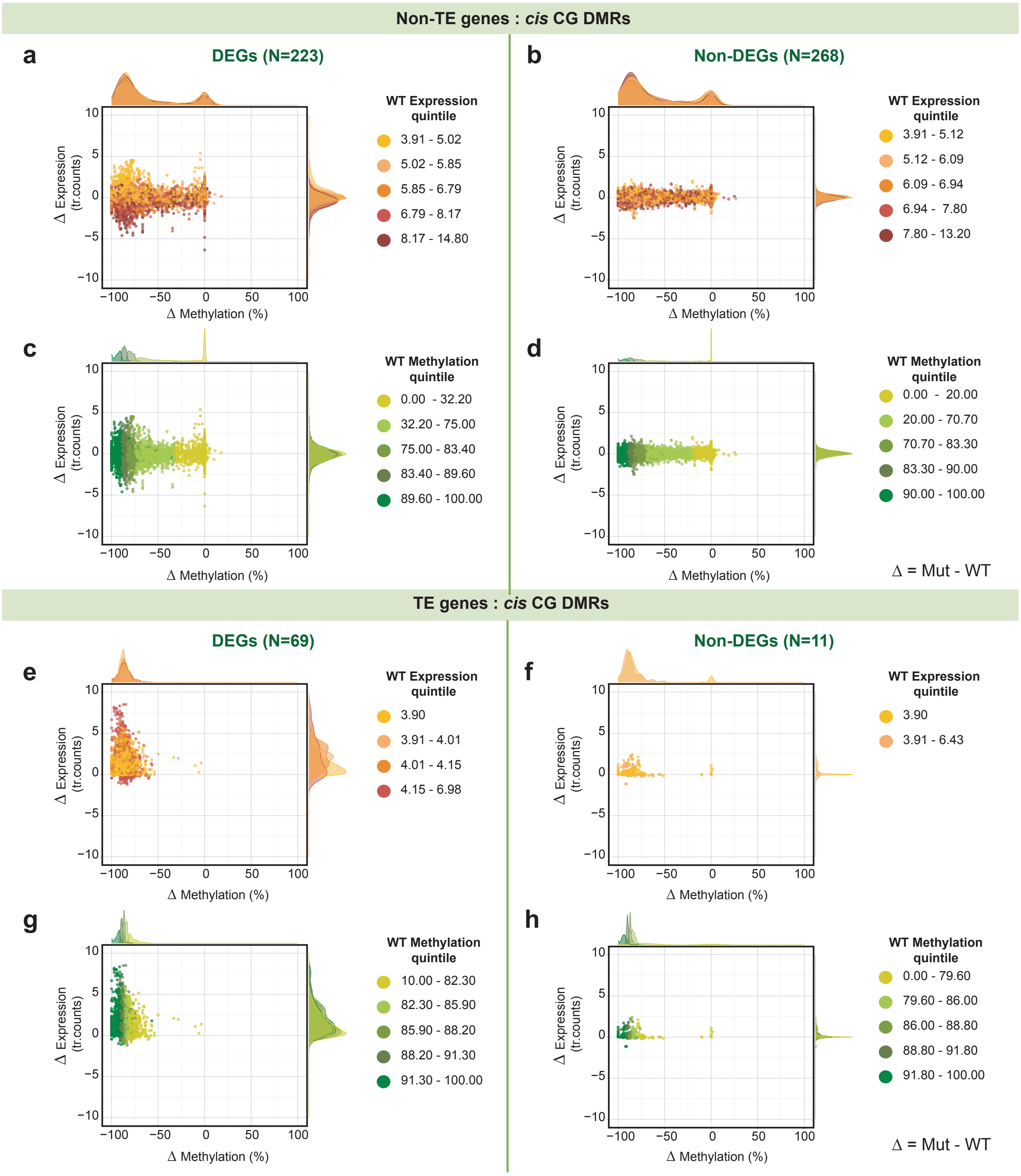

Figure S19. CG-DMRs in cis to Non-TE-genes (a-d) and TE-genes (e-h), colored by parental expression and methylation state. Scatter plots showing differences in CG methylation between *met1* mutants and wild-type plants against difference in gene expression. Dots in the scatter plot are colored by wild-type expression quintiles (a,b,e,f) and wild-type methylation quintiles (c,d,g,h) with x- and y-axis density distributions of each expression/methylation quintile. Expression levels are represented as transformed read counts and methylation levels are represented as %CG methylation in CG-DMRs.

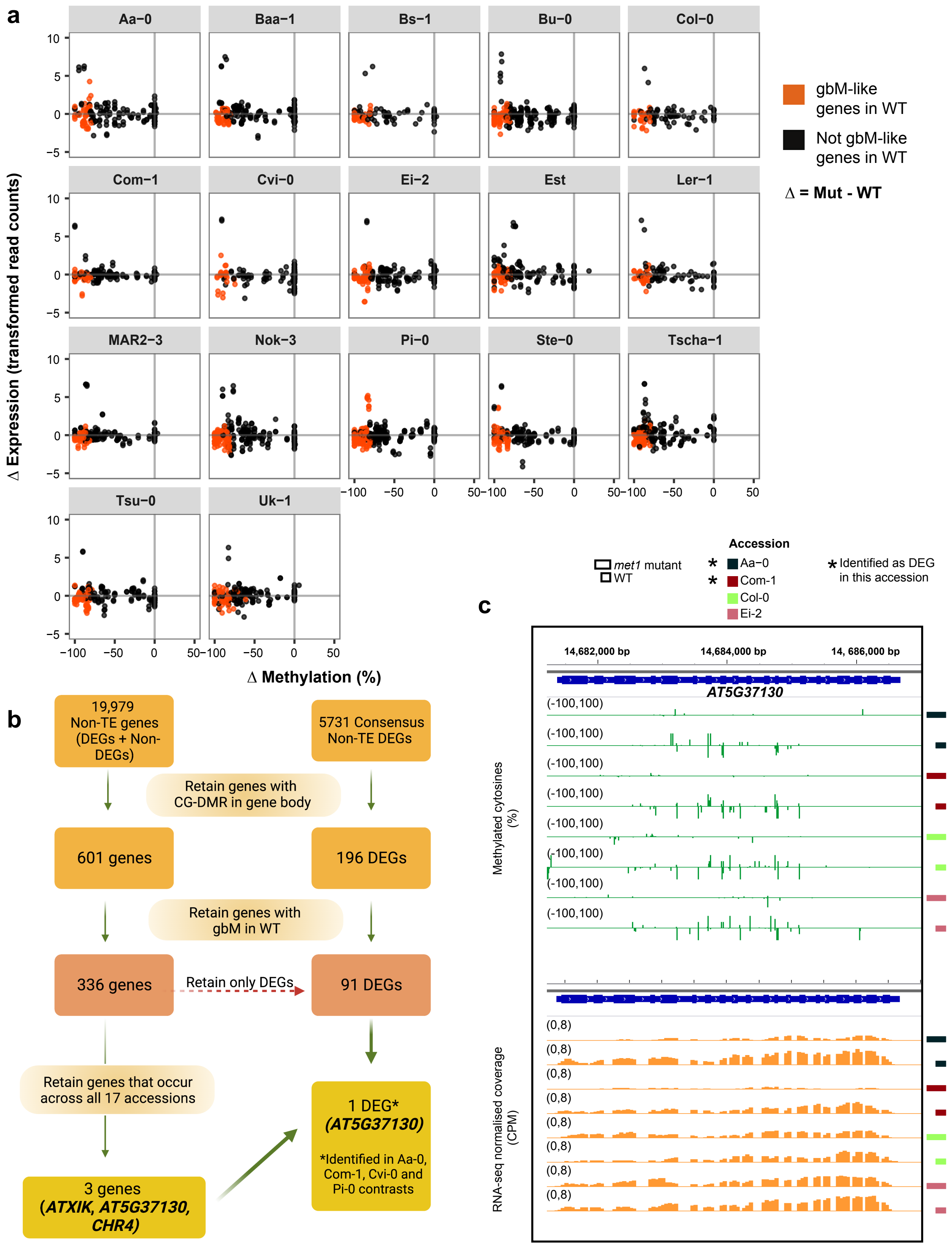

Figure S20. Variation in expression for wild-type gbM-like methylated genes in *met1* mutants of different accessions. (a) Methylation changes and associated gene expression changes for 91 gbM-like genes across 17 accessions. Orange colored dots represent gbM-like genes and black dots represent the same genes which are not gbM-like in other accessions. ΔMethylation represents *met1* - wild-type methylation, measured in % CG methylation level. ΔExpression represents *met1* - wild-type gene expression levels, measured in transformed read counts. (b) Diagram of identification of gbM-like genes across accessions. (c) Genome browser screenshot of methylated cytosines (% methylation in all cytosine contexts) and normalised RNA-seq read coverage (in CPM; counts per million) for four accessions in the *AT5G37130* locus.

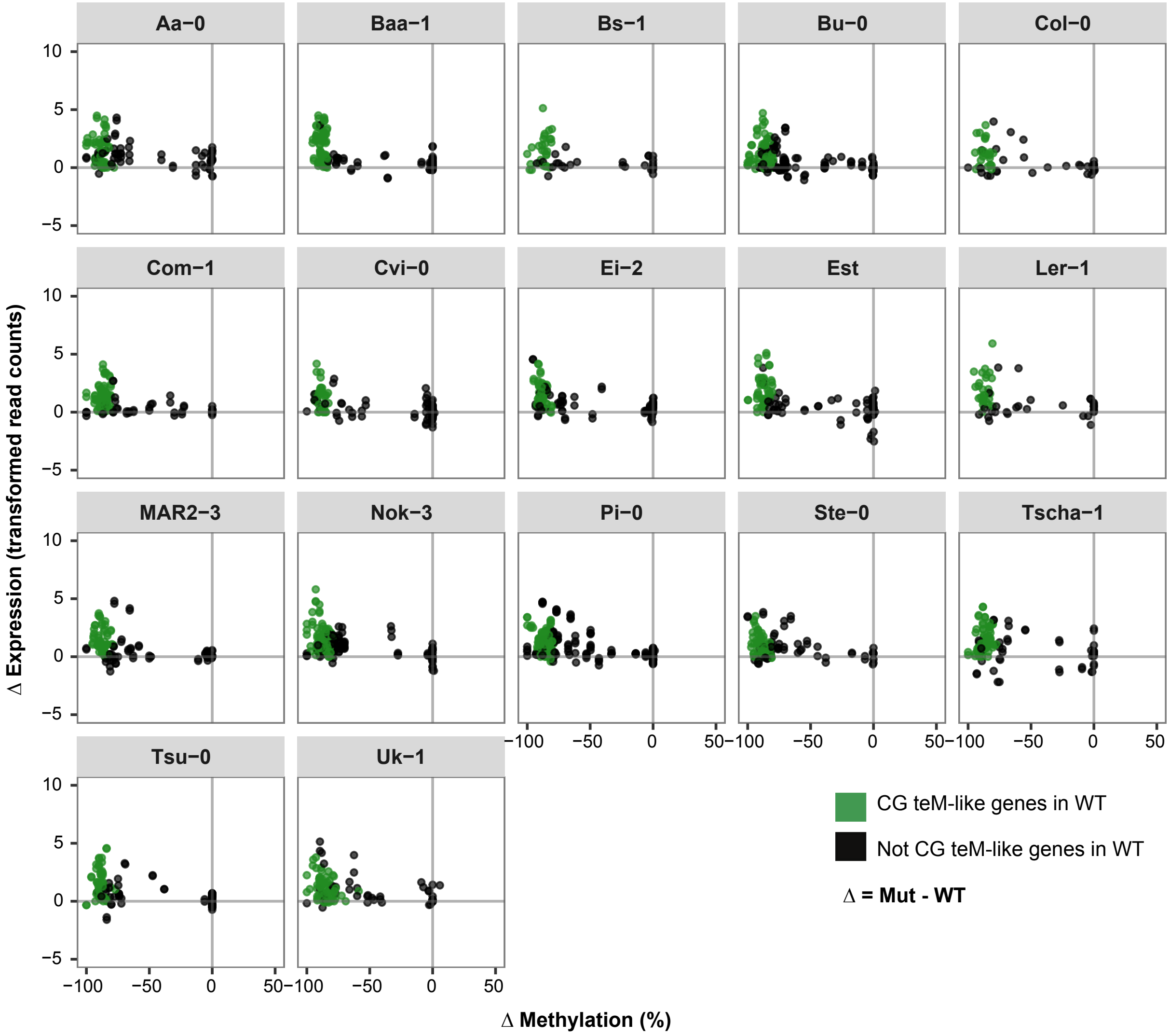

Figure S21. Methylation changes and associated gene expression changes for 57 CG teM-like genes across 17 accessions. Green colored dots represent CG teM-like genes and black dots represent the same genes which are not CG teM-like in other accessions.

ΔMethylation represents *met1* - wild-type methylation, measured in % CG methylation level.

ΔExpression represents *met1* - wild-type gene expression levels, measured in transformed read counts.

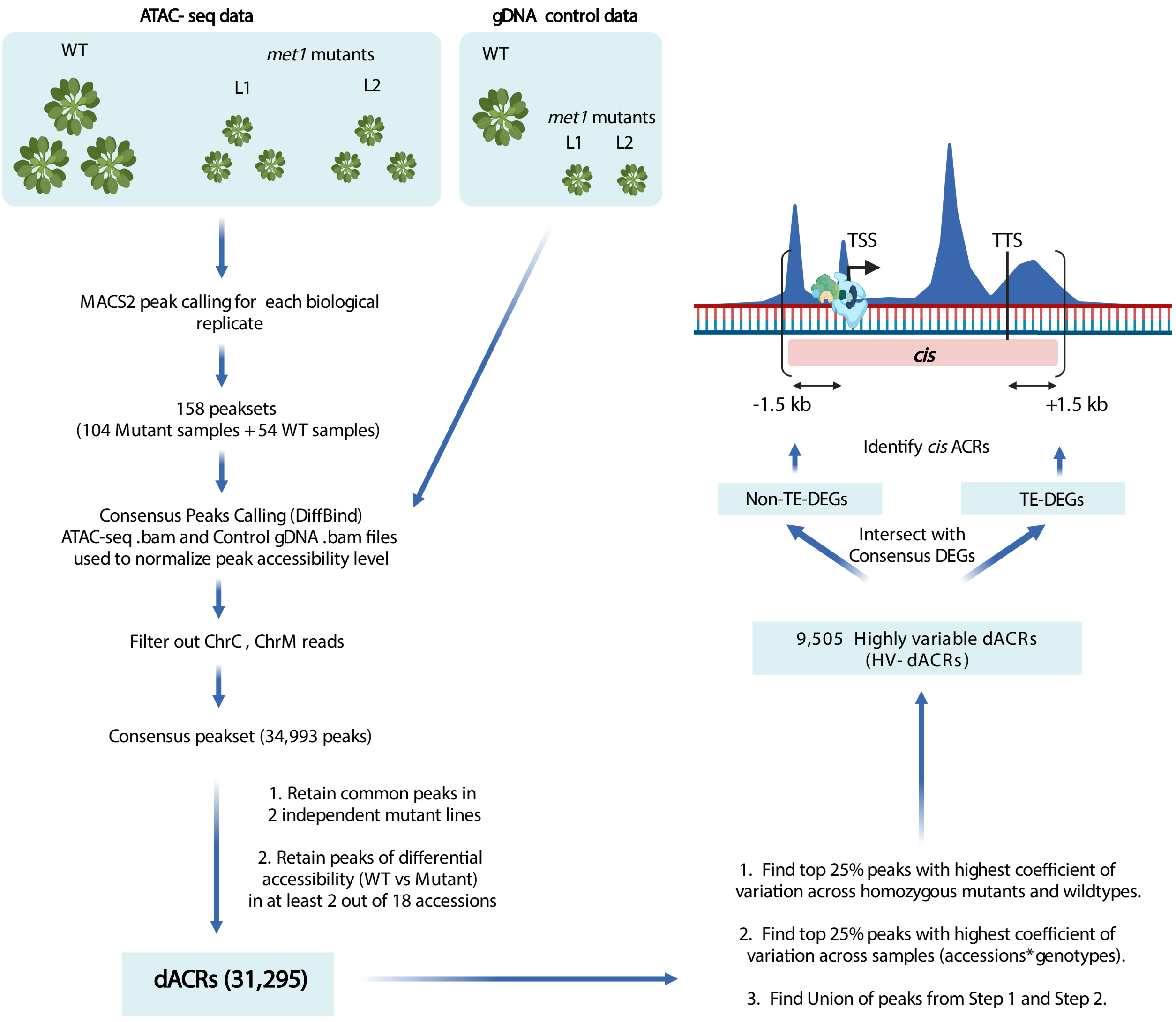

Figure S22. Diagram of ATAC-seq processing, generation of highly variable dACRs (HV-dACRs), and intersections with DEGs.

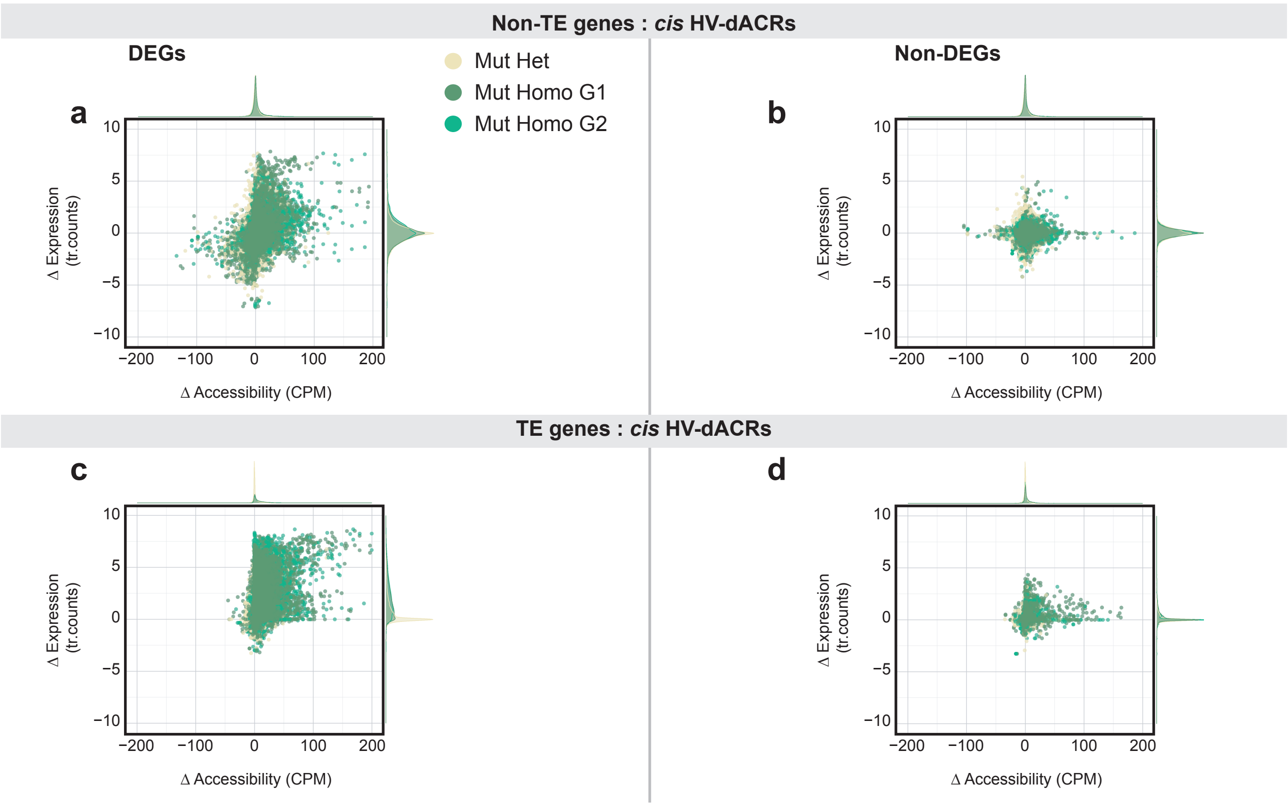

Figure S23. HV-dACRs in *cis* to TE genes (c,d) and Non-TE genes (a,b). Scatter plots showing difference in Chromatin accessibility between met1 mutants and wild-type plants against differences in gene expression. Dots in the scatter plot are colored by genotype of *met1* mutants; wildtypes ('WT'), heterozygous *met1* mutants ('Mut Het'), first generation homozygous *met1* mutants ('Mut Homo G1') and second generation homozygous *met1* mutants ('Mut Homo G2') with x- and y-axis density distributions of each genotype. Expression levels are represented as transformed read counts and accessibility levels are represented as TMM normalized values in counts per million (CPM).

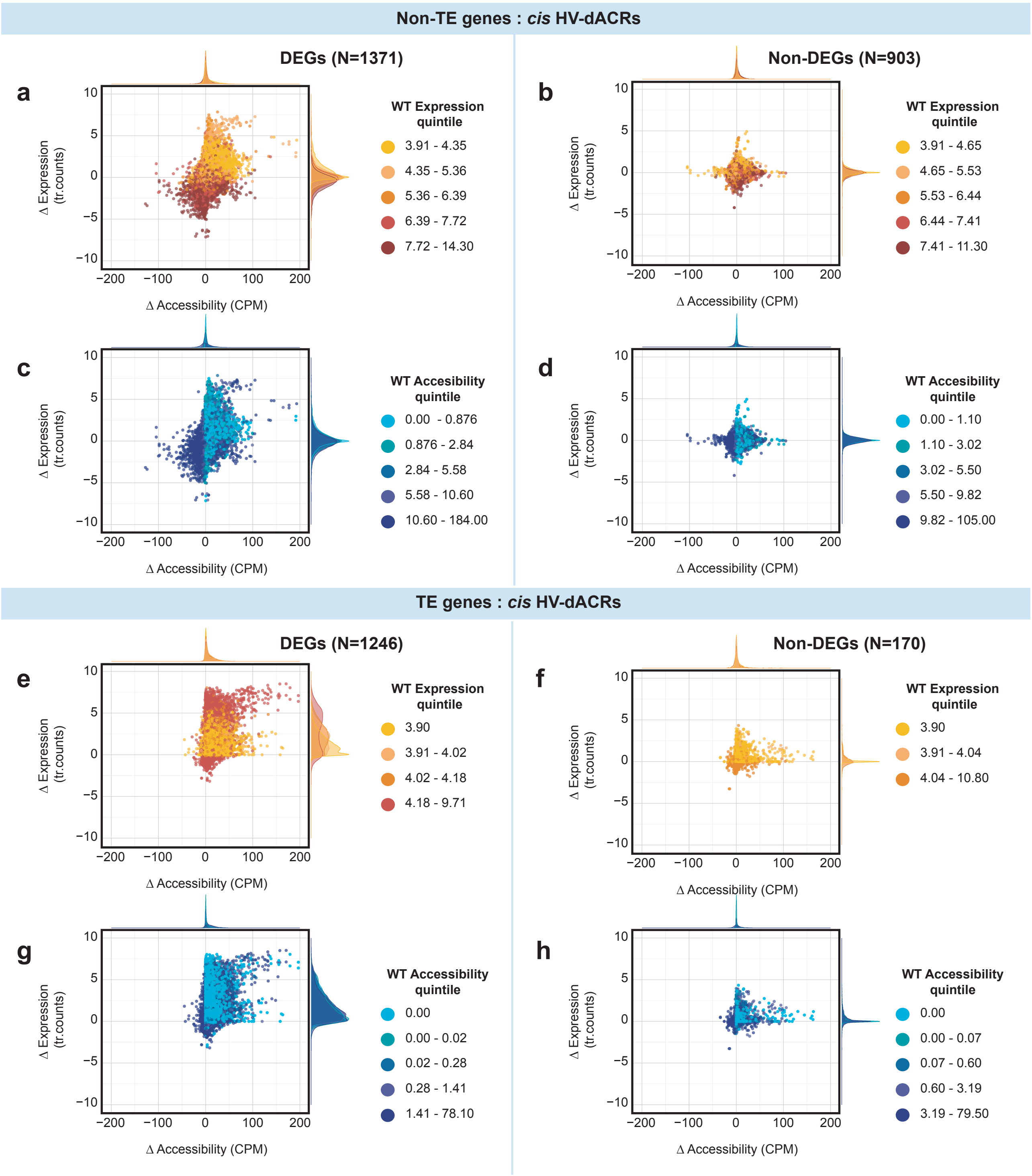

Figure S24. HV-dACRs in *cis* to TE-genes (a-d) and Non-TE-genes (e-h), colored by parental expression and chromatin accessibility state. Scatter plots showing differences in chromatin accessibility between *met1* mutants and wild-type plants against differences in gene expression. Dots in the scatter plot are colored by wild-type expression quintiles (a,b,e,f) and wild-type accessibility quintiles (c,d,g,h) with x- and y-axis density distributions of each expression/accessibility quintile. Expression levels are represented as transformed read counts and accessibility levels are represented as TMM normalized values in counts per million (CPM).

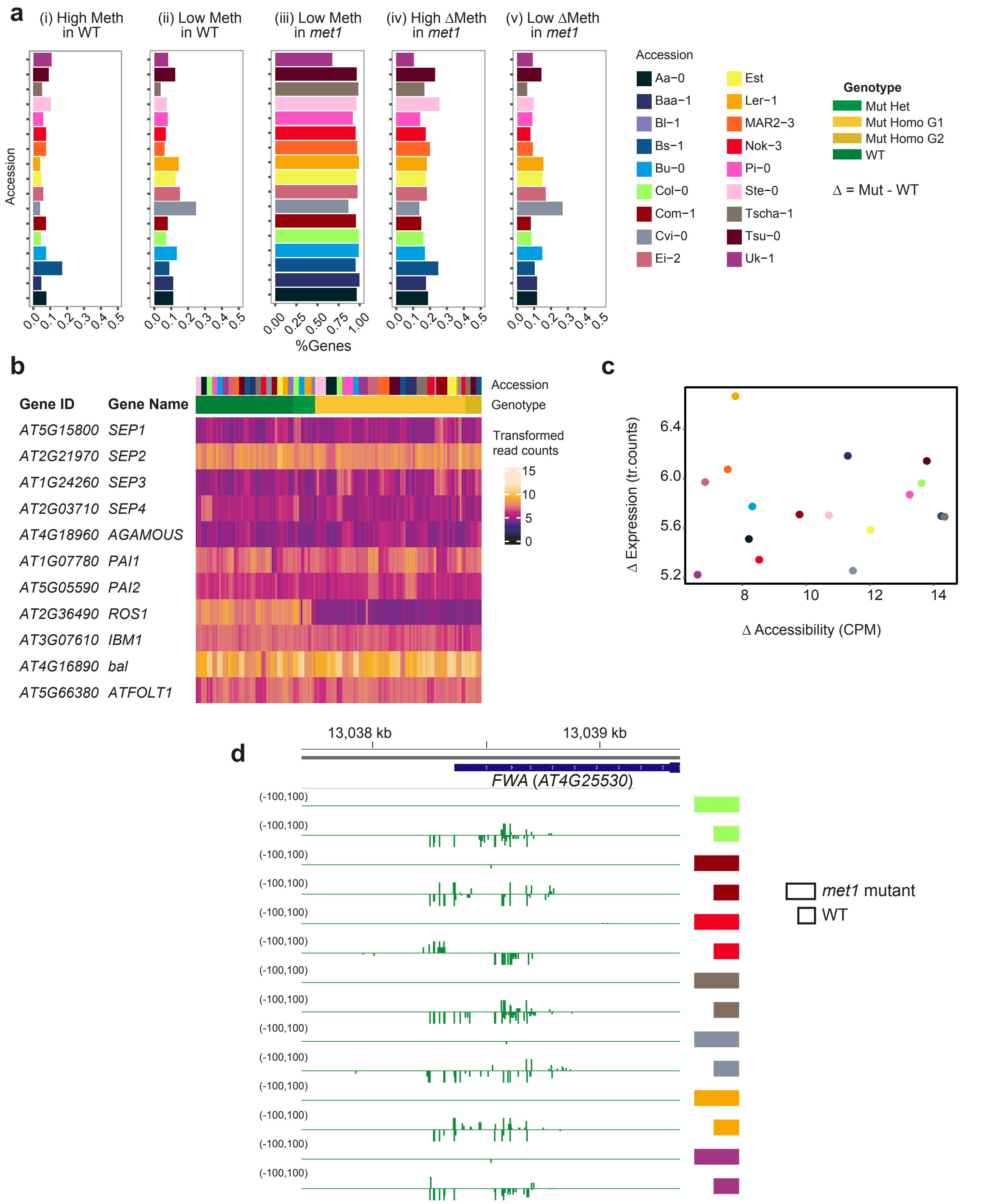

Figure S25. Variable epigenetic states of Non-TE genes across accessions. (a) Five panels showing fraction of genes in 17 accessions with (i) high CG methylation in wild type, (ii) low CG methylation in wild type, (iii) low CG methylation in *met1*, (iv) large methylation change in *met1*, (v) limited methylation change in met1 across 271 Non-TE genes. Colors represent different accessions. Low methylation is defined as CG methylation <= 10%, and high methylation is defined as CG methylation >= 90%. (b) Heatmap of transformed read counts at 11 epialleles across 158 RNA-seq libraries. The libraries are colored by accession-of-origin and genotype. (c) Scatterplot showing relationship between changes in accessibility and expression at the *FWA* locus. Accessibility is measured in counts per million (CPM) and expression is measured by transformed read counts. (d) Genome browser screenshot of methylated cytosines (all contexts) at the *FWA* locus for *met1* mutants and wild-type plants of seven accessions. Genotypes represented are wildtypes ('WT'), heterozygous *met1* mutants ('Mut Het'), first generation homozygous *met1* mutants ('Mut Homo G1') and second generation homozygous *met1* mutants ('Mut Homo G2').

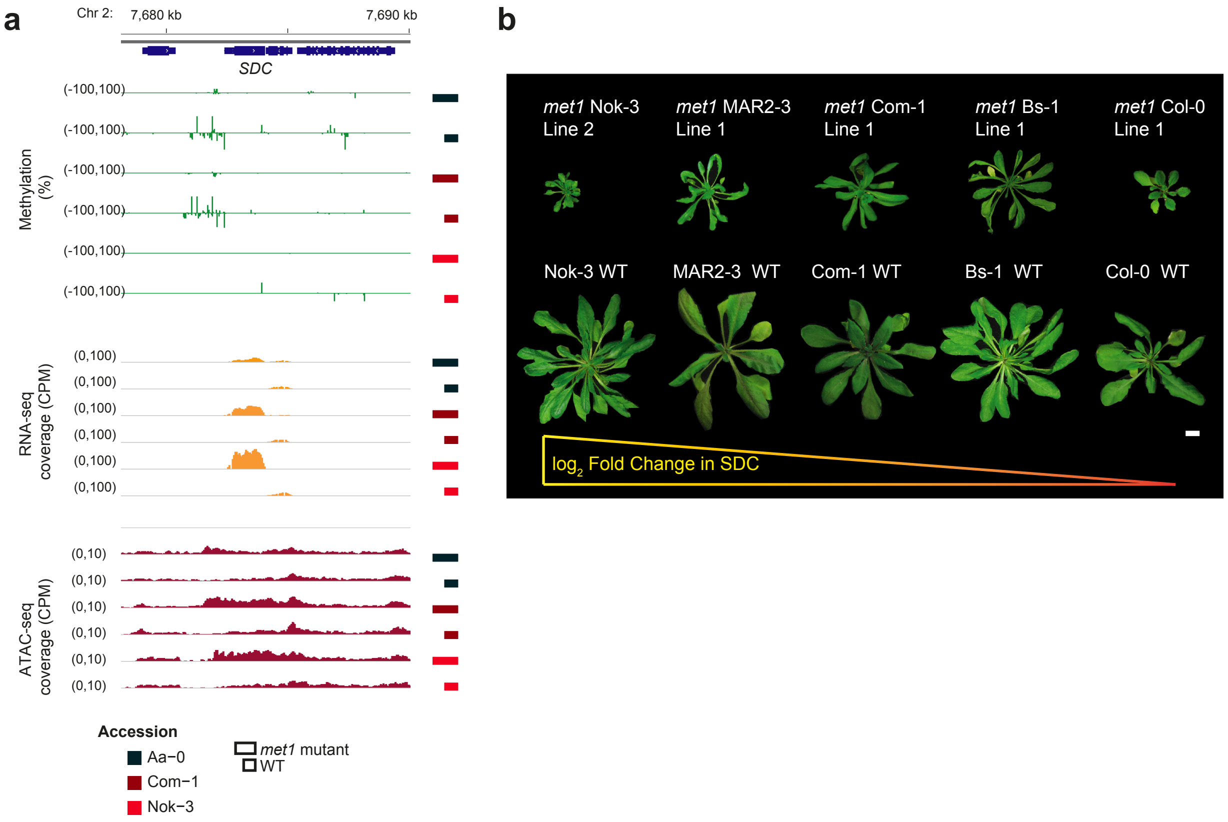

Figure S26. Epigenetic landscape at the *SDC* locus. (a) Genome browser screenshot of methylated cytosines, chromatin accessibility and RNA-seq read count in *met1* mutants and wildtypes of three accessions, Aa-0, Com-1 and Nok-3. (b) Rosettes of *met1* mutants and wild-type plants from five accessions ordered by $\log_2$ fold change in *SDC* expression. White scale bar denotes 1 cm.

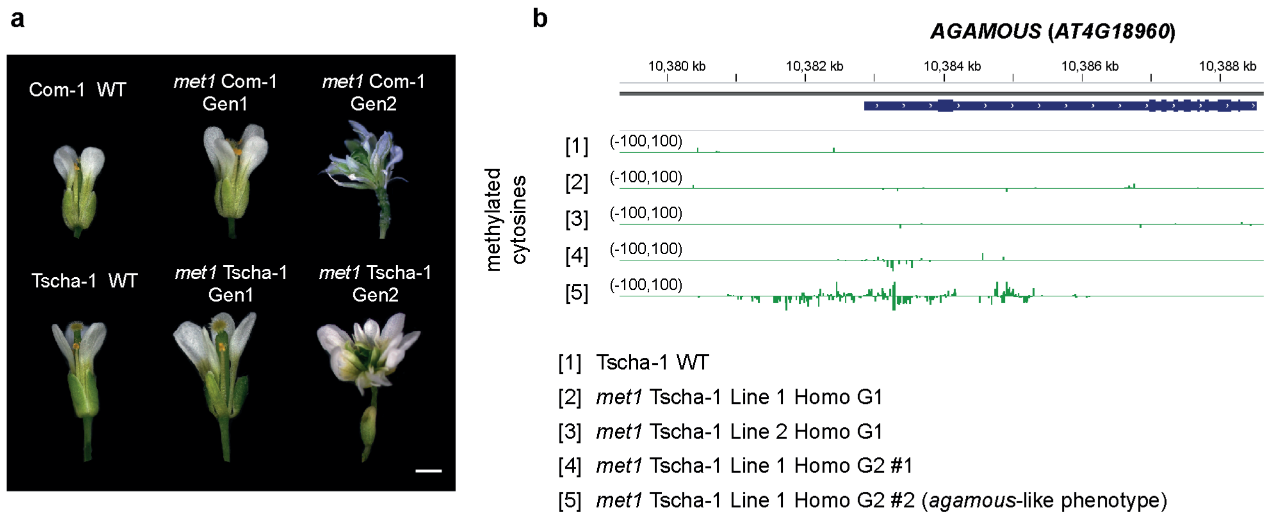

Figure S27. Transgenerational ag-like phenotypes in *met1* mutants of Com-1 and Tscha-1. A few *met1* mutant lines of the Tscha-1 and Com-1 accessions exhibited indeterminate flowers, a phenotype known to arise from genetic (Yanofsky et al. 1990) and epigenetic inactivation (Jacobsen et al. 2000) of the AG gene. BS-seq of one such line in Tscha-1 also showed an increase in methylation at this locus. (a) Flower phenotypes of wild-type and homozygous *met1* plants in two generations (G1 and G2). Scale bar denotes 1 mm. (b) Genome browser screenshot of methylated cytosines (all-contexts) at the *AG* locus for various Tscha-1 *met1* and wildtype lines. G1, Gen1; G2, Gen2; Homo, Homozygous.

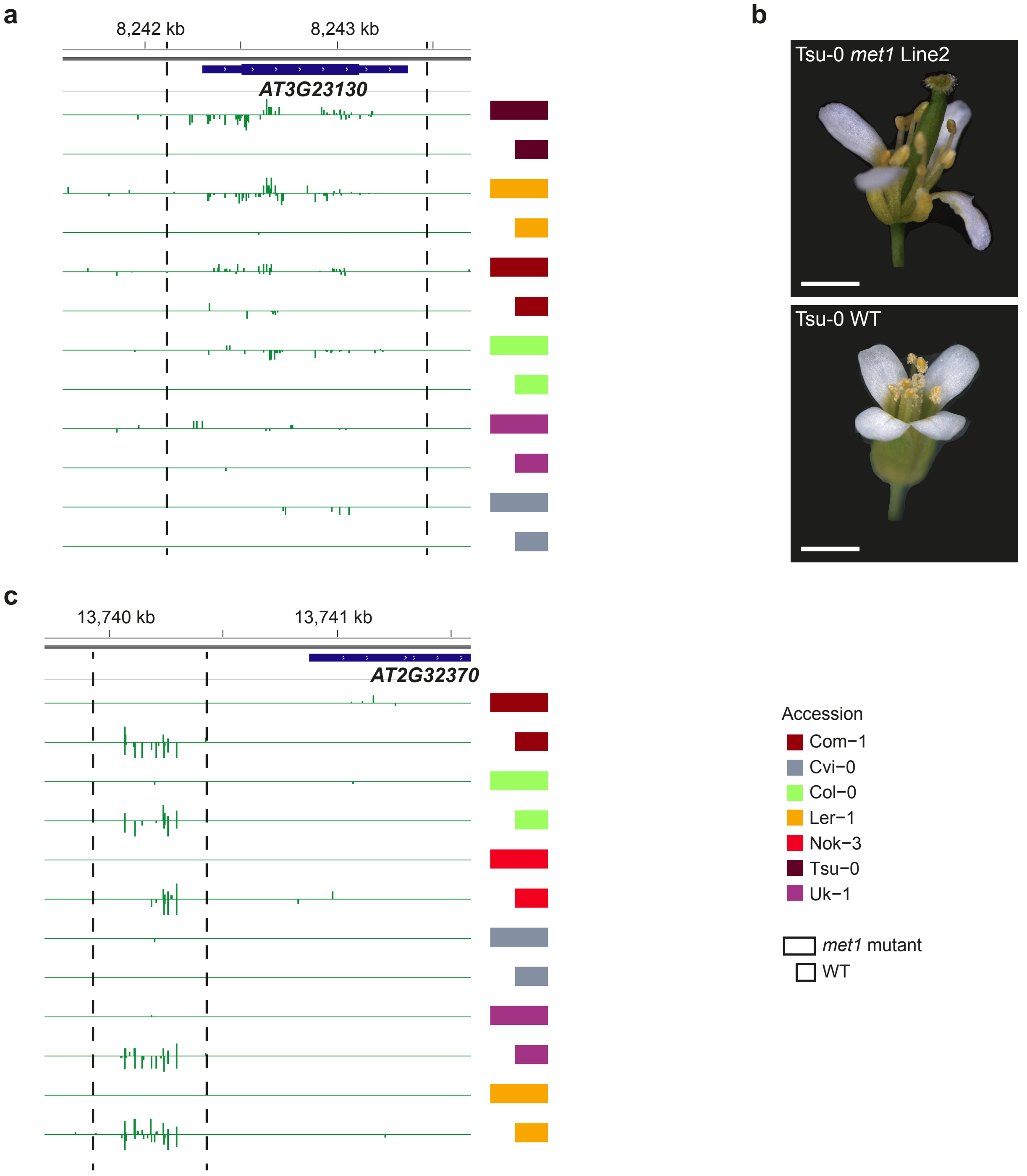

Figure S28. Variation in methylation levels at two epialleles in *met1* mutants and wild-type plants across a subset of accessions. (a) Genome browser view of methylated cytosines (all contexts) at the *SUP (AT3G23130)* locus. Gain of methylation in the gene body of *SUP* silences the gene and results in the formation of additional stamens (Jacobsen and Meyerowitz 1997). (b) representative image of a Tsu-0 *met1* mutant flower with nine stamens and a Tsu-0 WT flower with six stamens. (c) Genome browser view of methylated cytosines (all contexts) at the *HDG3 (AT2G32370)* locus. Scale bars represent 1mm.

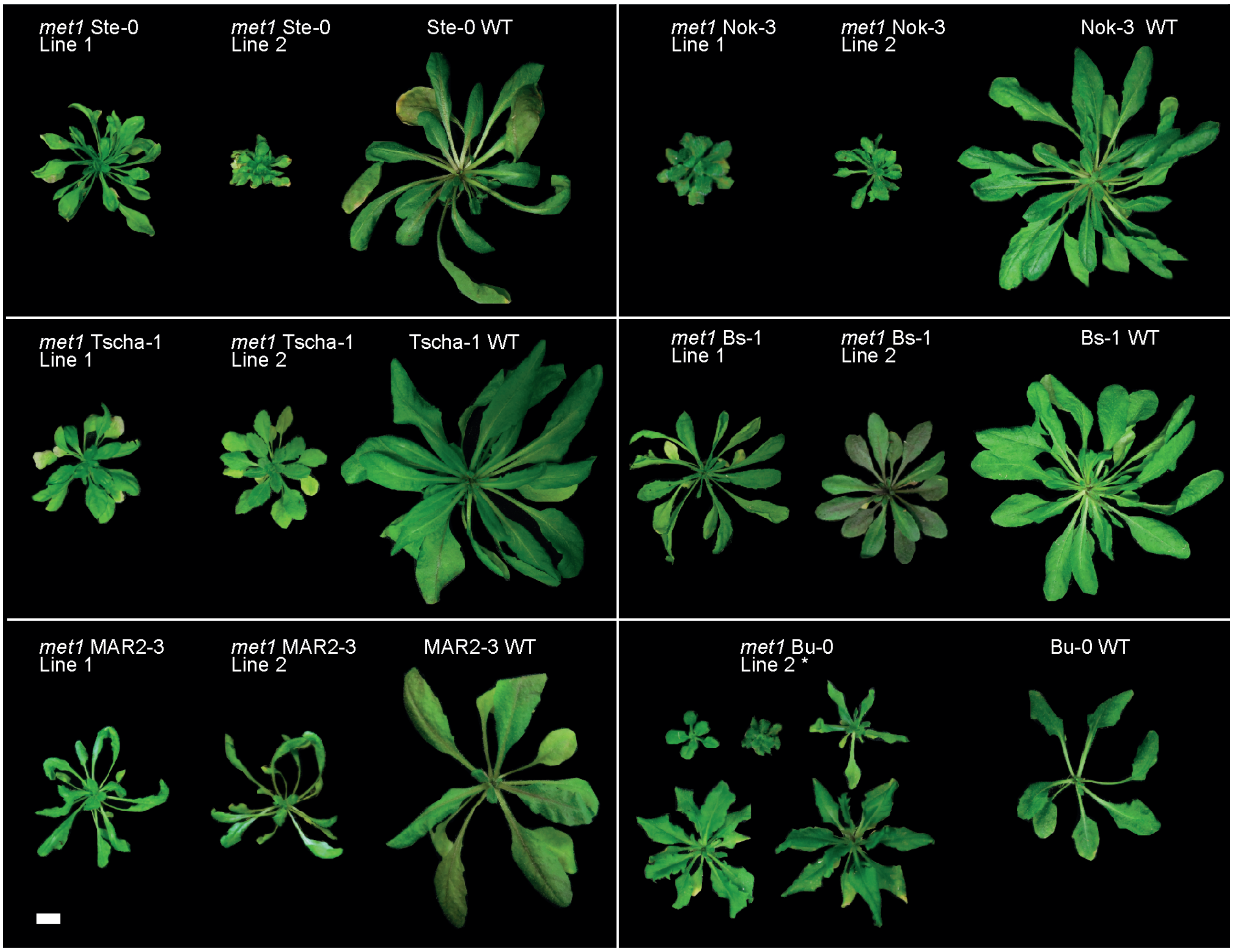

Figure S29. Rosettes of *met1* mutants in six accessions. Representative images of two independently derived mutant lines and a wild-type plant at six weeks after germination; scale bar denotes 1 cm. *met1* mutants of the accession Bu-0 are marked by an asterisk (*) since they were tetraploid and exhibited a wide range of phenotypes arising from different dosages of two mutant alleles and a wild-type allele.

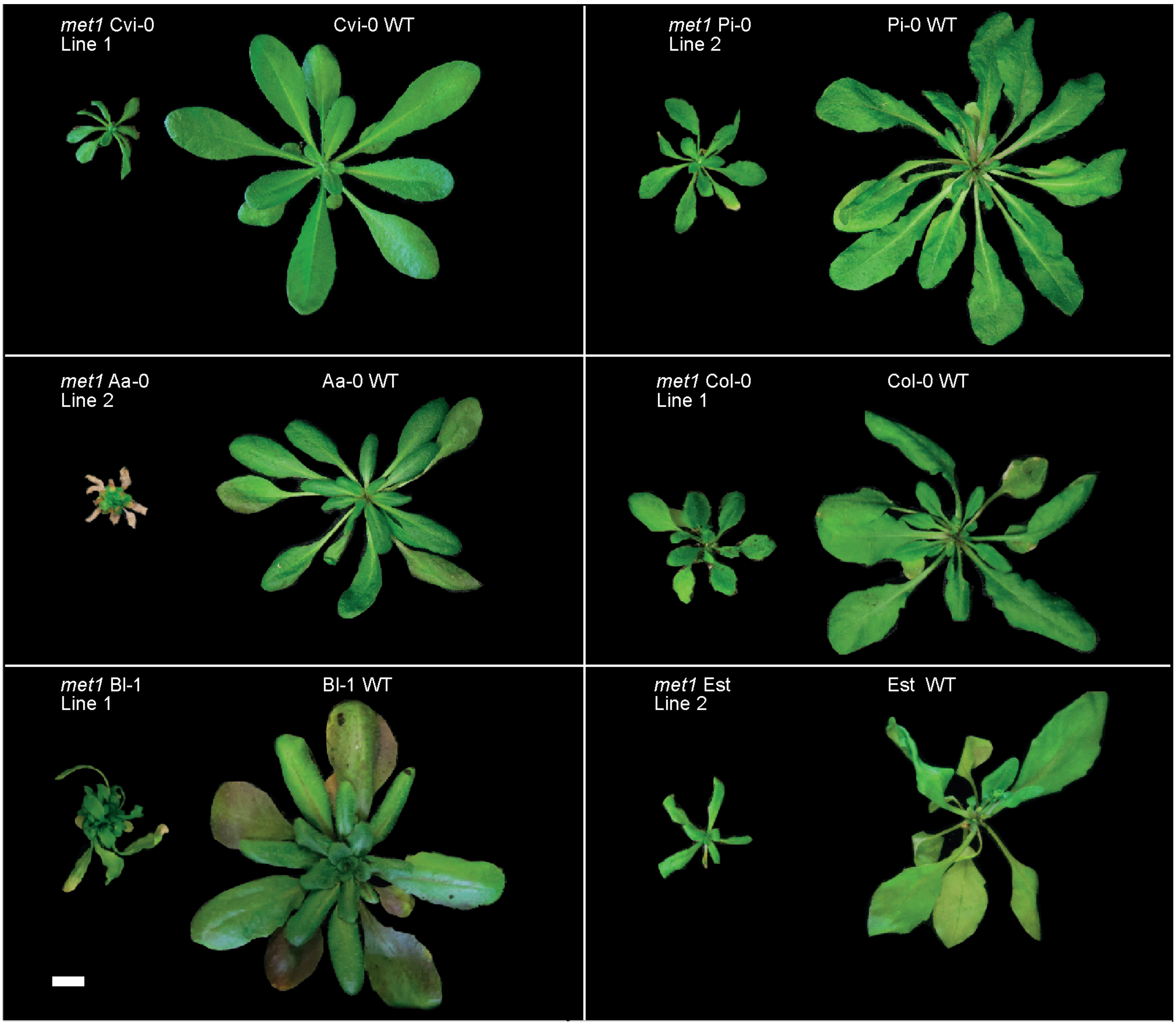

Figure S30. Rosettes of *met1* mutants in six accessions. Representative images of mutant and WT plants at six weeks after germination; white scale bar denotes 1 cm.

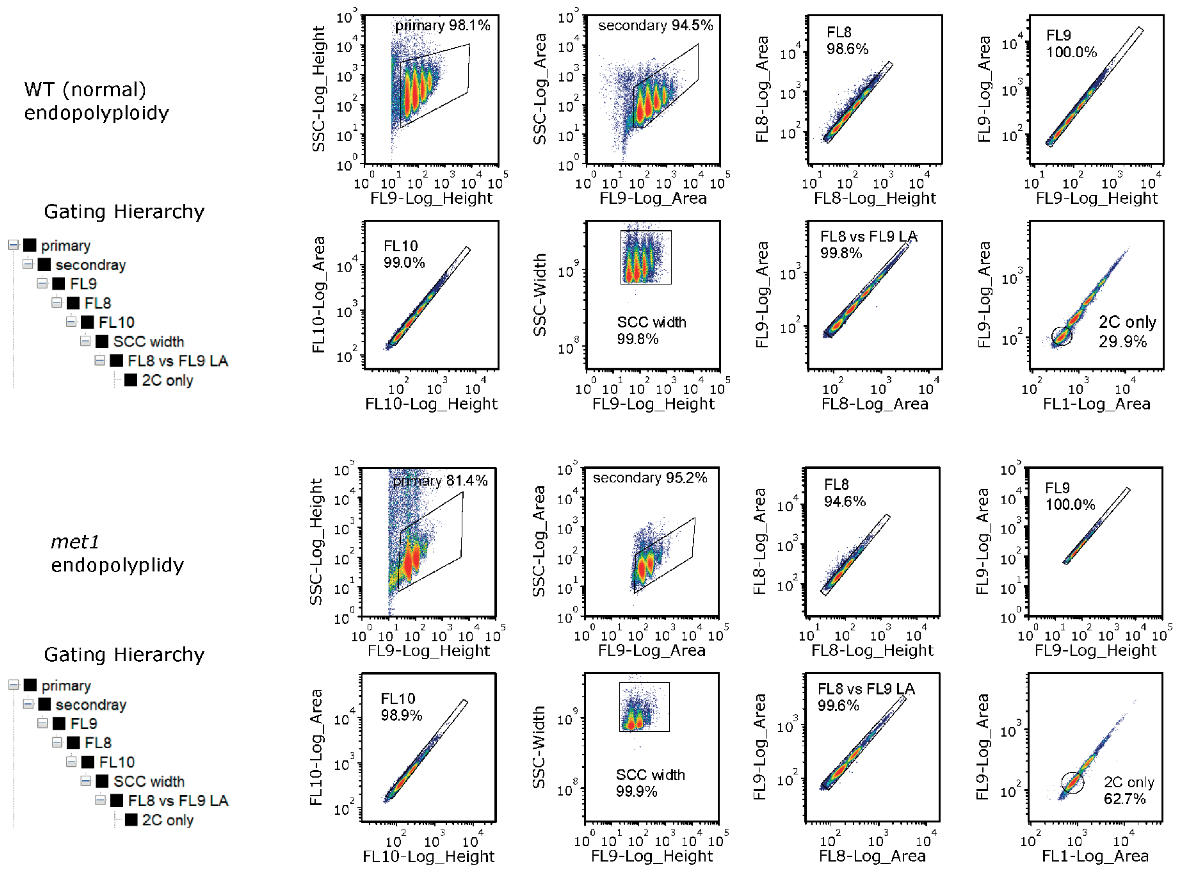

Figure S31. Reduced endopolyploidy in rosette leaf nuclei of *met1* mutants. An example of FACS gating profiles for WT and *met1* genotypes of the Nok-3 accession is shown.

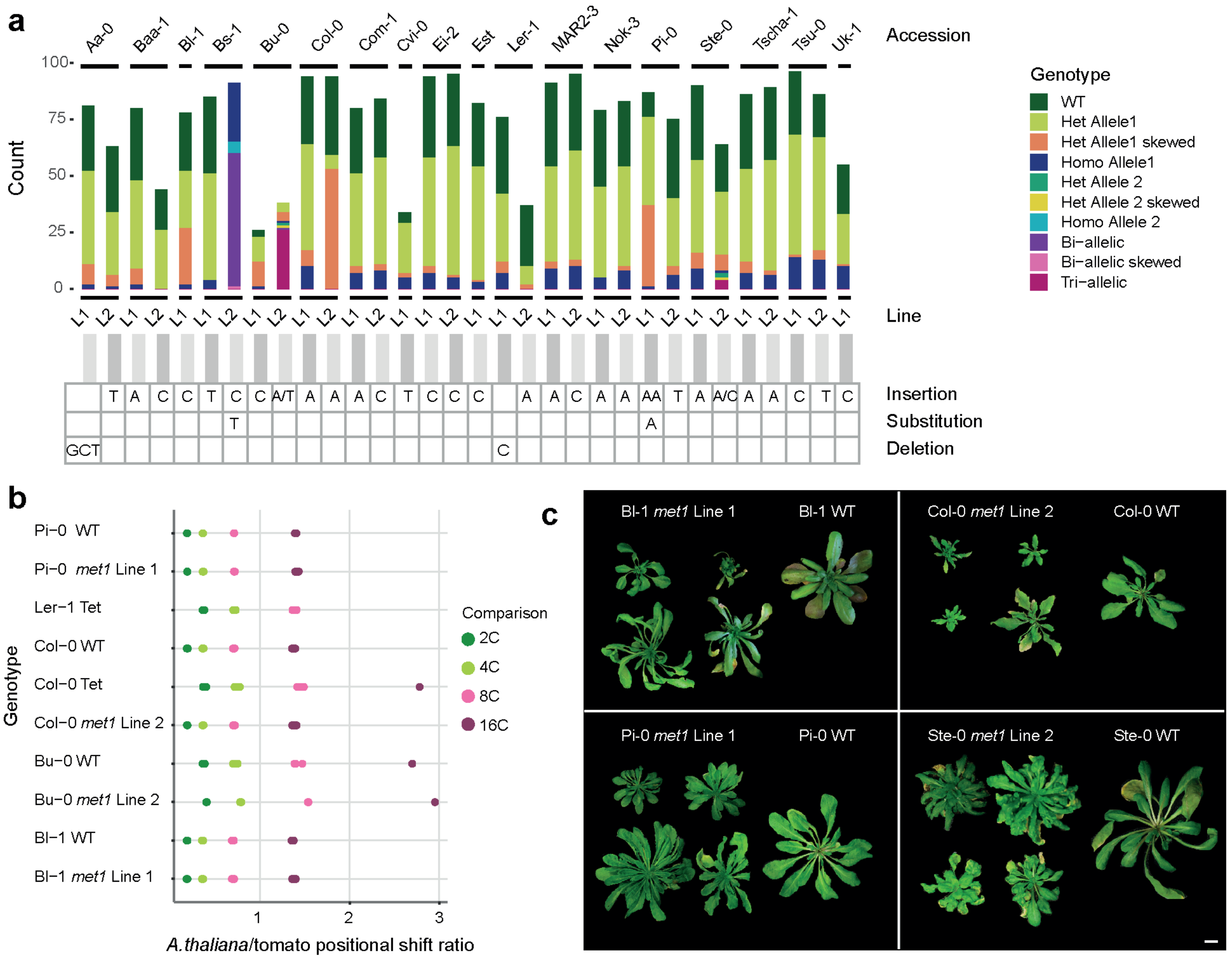

Figure S32. Segregation distortion in *met1* mutants and the presence of skewed heterozygous individuals. (a) Genotypes of segregating *met1* mutants representing sampled individuals and associated mutations for every line. (b) Scatter plot of endopolyploidy peak position ratios (from flow cytometry profiles) in candidate mutant lines and wild-type plants relative to the tomato internal standard. Col-0 and Ler-1 tetraploids ('Col-Tet' and 'Ler-Tet' respectively) were used as references for validating ploidy variation in candidate lines. (c) Phenotypic variation in heterozygous plants of Bl-1 Line 1, Col-0 Line 2, Pi-0 Line 1 and Ste-0 Line 2. Scale bar represents 1 cm.

REFERENCES

1. Yanofsky MF, Ma H, Bowman JL, Drews GN, Feldmann KA, Meyerowitz EM. The protein encoded by the Arabidopsis homeotic gene agamous resembles transcription factors. Nature. 1990;346:35–9.
2. Jacobsen SE, Sakai H, Finnegan EJ, Cao X, Meyerowitz EM. Ectopic hypermethylation of flower-specific genes in Arabidopsis. Curr Biol. 2000;10:179–86.
3. Jacobsen SE, Meyerowitz EM. Hypermethylated SUPERMAN epigenetic alleles in arabidopsis. Science. 1997;277:1100–3.
