# Supplementary material for: Canalization of genome-wide transcriptional activity in Arabidopsis thaliana accessions by MET1-dependent CG methylation
Source: Genome Biol. 2022 Dec 20;23:263. doi: 10.1186/s13059-022-02833-5 (PMC9768921; doi:10.1186/s13059-022-02833-5)
Supplement: Supplementary file 21 — Additional file 21. Extended Methods. [file 13059_2022_2833_MOESM21_ESM.pdf]

EXTENDED METHODS

Generation of consensus ATAC-seq peaks (ACRs) using DiffBind:

The following commands were used in R using the package "DiffBind" (where 'dataset_Acc' refers to the sample sheet containing details of MACS2-called peaks from individual libraries):

```
library(DiffBind)

dataset_Acc <- dba(sampleSheet="samplesheet_forDiffBind.csv")                    #1

dataset_Acc_consensus <- dba.peakset(dataset_Acc, consensus=-DBA_REPLICATE)      #2

dataset_Acc_consensus<- dba(dataset_Acc_consensus,
mask=dataset_Acc_consensus$masks$Consensus, minOverlap=1)                        #3

consensus_peaks <- dba.peakset(dataset_Acc_consensus, bRetrieve=TRUE)            #4

dataset_Acc<- dba.count(dataset_Acc, peaks=consensus_peaks,
score=DBA_SCORE_TMM_MINUS_FULL_CPM, summits=FALSE)
normCounts<- dba.peakset(dataset_Acc, bRetrieve=TRUE, DataType=DBA_DATA_FRAME)
```

To identify peak summits for each library (separate analysis, after completing steps #1-#4 above):

```
dataset_Acc<- dba.count(dataset_Acc, peaks=consensus_peaks, summits=TRUE,
score=DBA_SCORE_SUMMIT_POS)
summits <- dba.peakset(dataset_Acc, bRetrieve=TRUE, DataType=DBA_DATA_FRAME)
```

Feature intersections between DEGs, CG-DMRs and HV-dACRs

To intersect positions of each consensus DEG set (TE-DEG/Non-TE-DEG) with CG-DMRs, we identified the five closest CG-DMRs to each DEG. Based on their proximity to the DEG, each of these hits were classified as 'extended gene-body' (distances within 100bp upstream of the TSS and 100bp downstream of the TTS), 'cis upstream/downstream' (within 1.5kb upstream/downstream of the gene body) and 'trans' if not falling within the above classes. All DEGs with CG-DMR hits under the 'trans' category were filtered out. DEGs with unique or multiple 'gene-body' CG-DMRs were classified as 'GB' , while DEGs with unique/multiple CG-DMRs in gene-body and *cis*, or only in *cis* were classified as 'cis'.

Subsequently, gene expression counts and methylation counts for the corresponding CG-DMRs were extracted for these DEGs. Since many DEGs had multiple CG-DMRs associated with them, we only retained CG-DMRs which showed the highest difference in methylation level between mutant and WT for each mutant genotype, thereby aiming to represent only the strongest methylation signals that could explain gene expression differences.

For intersecting consensus DEGs with HV-dACRs, we used a similar approach, where the closest five HV-dACRs to each DEG were identified. After filtering out DEGs with HV-dACRs in 'trans', we retained all remaining DEGs (even with multiple 'gene-body' and 'cis' HV-dACRs) in a single category called 'cis'. For each DEG, only HV-dACRs with the highest difference in accessibility between mutant and WT for each mutant genotype were retained.

A more detailed explanation of the above methods is provided below :

1. Intersects between DEGs and CG-DMRs:

Consensus DEGs (from all accessions) were first split as 5731 Non-TE-DEGs and 1401 TE-DEGs. Next, each set was intersected with positions of consensus CG-DMRs (generated from all samples), using *bedtools closest*, to find the top 5 closest CG-DMRs to each DEG. Based on their proximity to the DEG, each of these hits were classified as 'gene-body' (closest distance >=-100 bp and <=100 bp), 'cis upstream' (closest distance <=-100 bp and >-1.5 kb), 'cis downstream' (closest distance >=100 bp and <=1.5 kb) and 'trans' (beyond 1.5 kb upstream or downstream).

#Example of command used (to find 5 closest hits of CG-DMRs to DEGs)

bedtools closest -a Consensus_NonTE-DEGs_coord.tab -b CG_DMRs_coord.bed -D a -k 5 > Consensus_NonTEDEGs_closestk5_CGDMRs.bed

All CG-DMR hits under the 'trans' category were filtered out. Next, the number of duplicate CG-DMR hits for each gene were counted. Each gene was further classified into the following categories: 'GB unique' (single CG-DMR hit in gene body), 'GB multi' (multiple CG-DMR hits in gene body), 'cis upstream' (single CG-DMR hit in cis upstream), 'cis downstream' (single CG-DMR hit in cis downstream), 'cis multi' (multiple cis CG-DMRs) and 'GB and cis' (multiple CG-DMRs in gene body and cis).

Genes classified as 'GB unique' and 'GB multi' were pooled together in a 'GB all' category. All remaining categories were pooled as a 'multiple cis regulatory' category. Gene

expression counts were obtained for genes in both pooled categories (for 158 samples and reduced to 73 samples to match the BS-seq dataset). Similarly, methylation levels for the CG-DMR corresponding to each gene were also obtained for 73 samples.

Next, each gene was scanned to examine duplicate CG-DMR hits and their methylation values. For each of the 73 samples, the CG-DMR carrying the highest difference between wild-type and mutant methylation levels was retained. These genes were subsequently plotted based on their methylation differences and gene expression differences.

2. Intersects between DEGs and HV-dACRs :

Each set of consensus DEGs (TE-DEGs and Non-TE-DEGs) was intersected with positions of HV-dACRs (generated from all samples) using *bedtools closest*, to find the top 5 closest HV-dACRs to each DEG. Based on their proximity to the DEG, each of these hits were classified as 'gene-body' (closest distance >=-100 bp and <=100 bp), 'cis upstream' (closest distance <=-100 bp and >-1.5 kb), 'cis downstream' (closest distance >=100 bp and <=1.5 kb) and 'trans' (beyond 1.5 kb upstream or downstream).

#Example of command used (to find 5 closest hits of HV-dACRs to DEGs)

bedtools closest -a Consensus_NonTEDEGs_coord.tab -b HV-dACRs_coord.bed -D a -k 5 > Consensus_NonTEDEGs_closestk5_HV-dACRs.bed

All HV-dACR hits under the 'trans' category were filtered out. All other genes and corresponding HV-dACR hits were pooled as a 'multiple cis regulatory' category. Gene expression counts were obtained for genes in each category (for 158 ATAC-seq samples and reduced to 73 samples to match the methylation dataset. Similarly, ATAC-seq accessibility levels (TMM) for the corresponding HV-dACR hit in each gene was also obtained for 158 samples and reduced to 73 samples.

Next, each gene was scanned to examine duplicate HV-dACR hits and their accessibility values. For each of the 73 samples, the HV-dACR carrying the highest difference between wild-type and mutant accessibility levels was retained. These genes were subsequently plotted based on these accessibility differences and gene expression differences.

3. Feature intersections between CG-DMRs and HV-dACRs (Additional File 2: Table S2):

CG-DMRs were intersected with positions of HV-dACRs using the *intersect* command of *bedtools*.
