# Supplementary material for: Canalization of genome-wide transcriptional activity in Arabidopsis thaliana accessions by MET1-dependent CG methylation
Source: Genome Biol. 2022 Dec 20;23:263. doi: 10.1186/s13059-022-02833-5 (PMC9768921; doi:10.1186/s13059-022-02833-5)
Supplement: Supplementary file 26 — Additional file 26: Review history. [file 13059_2022_2833_MOESM26_ESM.docx]

Review History

**First round of review**

**Reviewer 1**

**Were you able to assess all statistics in the manuscript, including the appropriateness of statistical tests used?**

Yes, and I have assessed the statistics in my report.

**Comments to author:**

In this study, the authors generate mutants of the DNA methyltransferase MET1 in 18 different ecotypes of the model species Arabidopsis and perform comprehensive genomic profiling in each of these mutant accessions. The key motivating question behind this study seems to be: to what extent does DNA methylation variation among closely related strains impact the transcriptome?

Given that the molecular function of MET1/DNMT1 is conserved since the common ancestor of all eukaryotes, one might expect loss of function to have similar impacts in ecotypes that share a recent common ancestor. However, this study shows substantial variation in the transcriptomes and phenotypes of closely related met1 mutants, suggesting that the interaction between DNA methylation and the underlying genetic sequence may evolve quickly, and that DNA methylation likely plays a role in maintaining consistency (or as the authors say, "canalization") in gene expression across diverse genetic lineages. The key finding of this manuscript is of interest to the field, and with a few corrections/revisions adequately addressed (outlined below) I believe this paper should be accepted for publication. I was also impressed with the thorough range of data presentation offered, which made understanding the data easier.

Major comments:

1) The authors rely heavily on the number of DEGs between WT and met1 mutants to draw conclusions about gene expression differences between accessions. This is problematic for two reasons. First, a gene may be differentially expressed in all accessions, but only reaching a threshold of significance in some. Figure 3E may actually illustrate this point for some genes - those down-regulated in one accession are often down-regulated in others, although outside the threshold of significance. Second, DEseq2 can be a little over-eager in calling differentially expressed genes when expression is low, as it predicts a fold-change value even if there are 0 reads in one of the comparison genotypes. Particularly if coverage is uneven across samples, this can (rarely) lead to artefacts and false positives in calling DEGs. I recommend that the authors employ a more quantitative approach to demonstrate the increase in expression variability between mutant met1 accessions. For example, the authors could calculate the coefficient of variation (CV) for every DEG between accessions (both WT and met1). If their main conclusion is correct, a violinplot of these values should show a greater range of CV values in the met1 group than the WT group.

2) My biggest issue with this paper is the work on chromatin accessibility and the analyses based on dACRs. The authors identify an enormous number of dACRs, such that it is difficult to draw meaningful conclusions about their relationship to gene expression or DNA methylation. With over 30,000 consensus dACRs identified, one must presume overlap with any gene or region of interest would occur by default (indeed, Fig. S11d suggests this). For associations between DMRs and expression the authors provide a control group of randomly selected genes, but such controls are not provided for associations with dACRs. Several conclusions drawn about chromatin accessibility are made with statements in the text such as: "In general, we found that genes with strongly reduced CG methylation in met1 mutants had a tendency to show drastic changes in chromatin accessibility" - if conclusions can be drawn based on the effect size of chromatin accessibility changes, then the authors should explore this more formally. I recommend the authors generate a smaller "stringent" set of dACRs with a larger effect size from which more accurate conclusions about relationship with expression/methylation can be drawn. Fig. 6b shows a subset of genes with substantial accessibility changes that are references in the text - is this not the biologically meaningful difference that should be viewed as "significant" rather than many thousand peaks all over the genome? I also think genome browser snapshots of the ATAC-seq data would help provide useful illustration and build confidence in the validity of the dACR peaks that have been called.

3) The boxplots in Fig. S11b also make me nervous about the biological relevance of smaller changes in chromatin accessibility. If met1 mutation is associated with increased chromatin accessibility overall, why should the heterozygous mutants or generation 2 mutants not also show increased accessibility relative to WT?

Minor corrections:

- The heatmaps of correlation coefficients in Figure 1D are not easy to interpret and don't offer much to the paper in my view. These could be removed and the remaining data in Figs. 1 and 2 combined into a single figure.

- p. 9 line 7 should reference Figure 2e, not 1e.

**Reviewer 2**

**Were you able to assess all statistics in the manuscript, including the appropriateness of statistical tests used?**

No, I do not feel adequately qualified to assess the statistics.

**Comments to author:**

The manuscript by Srikant et al reports the characterization of met1 mutants in different genetic backgrounds in Arabidopsis. The authors have obtained CRISRP null mutants for the methyltransferase MET1 in A. thaliana. MET1 is responsible for the deposition of methyl groups in CG context during replication. The 18 hypomethylated lines have been analyzed for their methylomes (paired BS-seq), transcriptome (RNAseq) and chromatin accessibility landscapes (ATAC-seq). All mutants display developmental phenotypes already described in Col-0 met1 mutants : dwarfism, flower development abnormalities. The methylome analyses shows that all lines have a strong reduction of CG methylation, confirming that the CRISPR mutations triggered a loss of function, in each tested genetic background. Surprisingly the impact of met1 mutation on gene expression (and compared to their relative WT control), is quite different for each accession.

I enjoyed reading this manuscript. The dataset is impressive and of high quality. The methods are clearly described, notably for computational analyses (command lines stated). The figures are clear and nicely presented. The literature cited is appropriate. Overall, this work is a very important ressource that opens the way for future studies.

I have two main comments that, I hope, will be helpful to authors.

First comment: One of most surprising finding of this work relies on the indirect effect of CG methylation on gene expression. MET1 direct targets (or epialleles) are rare and well-documented (see FWA, SDC, SUP, SNC1 etc) and stably found in the different genetic backgrounds (note that they are also stable inherited in met1 epiRILs for instance). In contrast, MET1 indirect targets are numerous but not clearly documented and seem to be background specific (hence more difficult to investigate). The indirect effect is cited (Page 20 line 18, Page 21 line 28, Page 25 line 48) but it could be highlighted in the abstract, as a main finding. While plant epigeneticists have focused for decades on a few targets, this might be only the tip of the iceberg. Accession specific CG methylation could have an adaptive role in each accession. The implication for evolution, while not yet understood, could be huge.

Second comment: Because the 18 genomes are not all available, all datasets have been mapped to Col-0, as a proxy. This is an important limitation of this work. While this limitation is clearly stated in the last sentence of the manuscript (a genome assembly would make the ressource even more valuable), I think the impact of structural variation (SV) has been underestimated in the discussion. SVs can originate from the genetic background, but also from the mutation it self (see CNV at the bal mutant in ddm1 mutant, from the Richards' lab). These SVs could affect TE insertion polymorphisms but also gene copy number for instance.

Minor comments:

1) Figure S23(b): I found interesting that ROS1 is the only MET1 conserved direct target in this Figure. Why not move this to the main figures?

2) Figure 6 and page 18 line 18: Maybe I missed this information: out of GbM genes (for each accession), how many are DEGs? What is the overlap of this category of genes in the different accessions (representing evolutionary conserved MET1 direct targets)?

3) Figure 3: Aren't ULP1 considered as TEs? or related to TEs? (one is a MULE)

4) Possible typo?: Col-o / Col-0 (e.g page 26 line 4).

**Authors Response**

**Point-by-point responses to the reviewers’ comments:**

Reviewer #1:

#1: The authors rely heavily on the number of DEGs between WT and met1 mutants to draw conclusions about gene expression differences between accessions. This is problematic for two reasons. First, a gene may be differentially expressed in all accessions, but only reaching a threshold of significance in some. Figure 3E may actually illustrate this point for some genes - those down-regulated in one accession are often down-regulated in others, although outside the threshold of significance. Second, DEseq2 can be a little over-eager in calling differentially expressed genes when expression is low, as it predicts a fold-change value even if there are 0 reads in one of the comparison genotypes. Particularly if coverage is uneven across samples, this can (rarely) lead to artefacts and false positives in calling DEGs. I recommend that the authors employ a more quantitative approach to demonstrate the increase in expression variability between mutant met1 accessions. For example, the authors could calculate the coefficient of variation (CV) for every DEG between accessions (both WT and met1). If their main conclusion is correct, a violinplot of these values should show a greater range of CV values in the met1 group than the WT group.

*Response: We first removed genes with low read count across all samples, which left 21,675 genes that were used to call DEGs (under the two-factor interaction model ~genotype + accession + genotype:accession; where genotype = wild-type or mutant; using nbinomWald test). With these DEGs, we generated the 18 accession-specific contrasts and the all-met1-vs-all-wild-type contrast. We are aware that DESeq2 can infer large changes for genes with very low expression in some samples. In our study, it is likely that many such genes would be TE-genes, since TEs are typically silenced in wild type and activated upon hypomethylation, or imprinted genes that are generally silenced in vegetative tissue and also activated by hypomethylation.*

*To demonstrate that variation in expression levels across accessions is higher for met1 than wild-type samples, we extracted transformed read counts for each of the 158 samples across 10,151 Non-TE-DEGs and 1,524 TE-DEGs (combined set of DEGs identified from all-met1-vs-al-wild-type and 17 accession-specific contrasts) and generated a violin plot of CV values (Figure S6). The mutants indeed show significantly higher variation in expression levels than wild-type samples (Wilcoxon test p-value < 2.2e-16), with the difference being more prominent for TE-DEGs than Non-TE-DEGs. This is probably due to TE-DEGs being more widely shared across accessions than Non-TE-DEGs, and TEs being very often completely silenced in wild type, and therefore varying less between wild-type samples. We generated similar plots for the 291 universal DEGs across all accessions. Since these are mostly TE-DEGs, a stark difference in CV distribution is observed between mutants and wild-type samples.*

*These results are currently presented as an additional supplementary figure (Figure S6). Considering that the manuscript contains over 30 supplementary figures, we would like to ask for guidance whether or not to include Figure S6 in the final set of supplementary figures.*

#2: My biggest issue with this paper is the work on chromatin accessibility and the analyses based on dACRs. The authors identify an enormous number of dACRs, such that it is difficult to draw meaningful conclusions about their relationship to gene expression or DNA methylation. With over 30,000 consensus dACRs identified, one must presume overlap with any gene or region of interest would occur by default (indeed, Fig. S11d suggests this). For associations between DMRs and expression the authors provide a control group of randomly selected genes, but such controls are not provided for associations with dACRs. Several conclusions drawn about chromatin accessibility are made with statements in the text such as: "In general, we found that genes with strongly reduced CG methylation in met1 mutants had a tendency to show drastic changes in chromatin accessibility" - if conclusions can be drawn based on the effect size of chromatin accessibility changes, then the authors should explore this more formally. I recommend the authors generate a smaller "stringent" set of dACRs with a larger effect size from which more accurate conclusions about relationship with expression/methylation can be drawn. Fig. 6b shows a subset of genes with substantial accessibility changes that are references in the text - is this not the biologically meaningful difference that should be viewed as "significant" rather than many thousand peaks all over the genome? I also think genome browser snapshots of the ATAC-seq data would help provide useful illustration and build confidence in the validity of the dACR peaks that have been called.

*Response: We agree that the large number of dACRs increases the chance of overlaps with genes, therefore making it more challenging to accurately interpret relationships between dACRs, DEGs and DMRs. Therefore, we reduced the 31,295 dACRs to obtain 9,505 highly variable dACRs (HV-dACRs) using the following criteria: The coefficients of variation (CV) of mean accessibility levels between homozygous mutants and wild-type samples, and across all samples (acessions*genotype) were identified for all dACRs, and only the dACRs in the top 25% for both were retained, resulting in 9,505 highly variable dACRs (HV-dACRs). This is described in Figure S22 of the revised manuscript. A UMAP projection and heatmap of accessibility levels across HV-dACRs is shown in revised Figure 3c-d. The HV-dACRs were subsequently intersected with DEGs, and the variation in expression and accessibility at these DEGs is shown in Figure S23 and Figure S24.*

*When intersecting the HV-dACRs with DEGs and DMRs, we found fewer genes (N=164) carrying both DMRs and HV-dACRs in cis (compared to N=407 when we used all dACRs). While the trends remain overall similar to our previous results, we find a clearer relationship between DEGs, DMRs and dACRs - where highly hypomethylated genes tend to exhibit increased chromatin accessibility and upregulated gene expression in met1 mutants (Figure 5b-c). We have also elaborated on these observations in the main text of the revised manuscript.*

*In Figure 5a of the revised manuscript, we show examples of DEGs with different epigenetic features, and also include genome browser screenshots (methylation, normalised RNA-seq read coverage, normalised ATAC-seq read coverage) of ROS1, which seems to be similarly downregulated across all accessions (as also observed by Reviewer 2, who suggested that this gene could be highlighted among the main figures). Genome browser tracks have also been updated in Figure S26 of the revised manuscript, where we examined the SDC locus in three accessions.*

#3: The boxplots in Fig. S11b also makes me nervous about the biological relevance of smaller changes in chromatin accessibility. If met1 mutation is associated with increased chromatin accessibility overall, why should the heterozygous mutants or generation 2 mutants not also show increased accessibility relative to WT?

*Response: We have regenerated boxplots of mean accessibility levels using the 9,505 HV-dACRs (Figure S13a), and the distinction between genotypes is now clearer.*

#4: The heatmaps of correlation coefficients in Figure 1D are not easy to interpret and don't offer much to the paper in my view. These could be removed and the remaining data in Figs. 1 and 2 combined into a single figure.

*Response: We have combined Figures 1 and 2 into the new Figure 1. The additional heatmaps from Figure 1 were moved to the new Figure S1.*

#5: p. 9 line 7 should reference Figure 2e, not 1e.

*Response: Corrected.*

Reviewer #2:

#1: One of most surprising finding of this work relies on the indirect effect of CG methylation on gene expression. MET1 direct targets (or epialleles) are rare and well-documented (see FWA, SDC, SUP, SNC1 etc) and stably found in the different genetic backgrounds (note that they are also stable inherited in met1 epiRILs for instance). In contrast, MET1 indirect targets are numerous but not clearly documented and seem to be background specific (hence more difficult to investigate). The indirect effect is cited (Page 20 line 18, Page 21 line 28, Page 25 line 48) but it could be highlighted in the abstract, as a main finding. While plant epigeneticists have focused for decades on a few targets, this might be only the tip of the iceberg. Accession specific CG methylation could have an adaptive role in each accession. The implication for evolution, while not yet understood, could be huge.

*Response: We agree that indirect effects of knocking out MET1 on gene expression are likely to be common, especially because many Non-TE-DEGs do not have a nearby CG-DMR or HV-dACR. The following new sentence in the abstract emphasizes this finding:*

*“Incidentally, most differentially expressed genes do not exhibit altered chromatin accessibility or CG methylation in cis, suggesting that the absence of MET1 can have profound indirect effects on gene expression that vary substantially from accession to accession.”*

#2: Because the 18 genomes are not all available, all datasets have been mapped to Col-0, as a proxy. This is an important limitation of this work. While this limitation is clearly stated in the last sentence of the manuscript (a genome assembly would make the resource even more valuable), I think the impact of structural variation (SV) has been underestimated in the discussion. SVs can originate from the genetic background, but also from the mutation it self (see CNV at the bal mutant in ddm1 mutant, from the Richards' lab). These SVs could affect TE insertion polymorphisms but also gene copy number for instance.

*Response: We agree that accession-specific genomes will greatly enhance the value of our dataset. We had tried to use information on TE presence-absence variants from [1], identified from short-read sequencing data using the SPLITREADER pipeline [2]. However, we found several reads mapping to regions annotated as “absence-variants” relative to Col-0, likely arising due to mapping artefacts or differences in mapping parameters. Therefore, we did not pursue this analysis further. With regards to structural variants arising due to the MET1 mutation, we agree that TE mobilization could be a confounder, but it is usually only seen after several rounds of inbreeding [3-5]. Our study mostly used first-generation homozygous mutants and the impact of new TE insertions will hopefully be minor.*

*As suggested, we elaborate on the importance of genetic variation in the discussion, citing relevant literature. We also discuss the value of complete genome assemblies instead of having to rely on the Col-0 reference genome.*

#3: Figure S23(b): I found interesting that ROS1 is the only MET1 conserved direct target in this Figure. Why not move this to the main figures?

*Response: ROS1 is downregulated in all accessions, even though it does not pass the significance threshold when calling DEGs for some accessions, and therefore is not among the universal Non-TE-DEGs (Figure 2d). Considering the well-known interplay between MET1 and ROS1 in maintaining methylation levels [6], we agree that the conserved effects of MET1 mutation on ROS1 downregulation in different accessions should be highlighted. We now show genome browser screenshots of methylation, RNA-seq read coverage and ATAC-seq read coverage at the ROS1 locus for three accessions in Figure 5a.*

#4: Figure 6 and page 18 line 18: Maybe I missed this information: out of GbM genes (for each accession), how many are DEGs? What is the overlap of this category of genes in the different accessions (representing evolutionary conserved MET1 direct targets)?

*Response: Out of 19,979 Non-TE genes with sufficient read counts, 601 have a CG-DMR in their gene-body. From these, 336 genes are gbM-like in wild type of at least one accession, with all 336 having lost almost all methylation substantially (reduction to under 10%) in the corresponding met1 mutant. Ninety-one out of the 336 genes are DEGs in at least one accession. We have now included a schematic of how we identified the various gbM-like genes, and a genome browser screenshot of gene body methylation and RNA-seq normalised read coverage at the AT5G37130 locus in Figure S20. We did not examine gbM-like DEGs that do not overlap with DMRs.*

#5: Figure 3: Aren't ULP1 considered as TEs? or related to TEs? (one is a MULE)

*Response: ULP-1 sequences are known to be present within Mutator-like transposable elements in Cucumis melo ("CUMULEs"), but there is no functional evidence that similar CUMULEs in rice and A. thaliana assist in TE mobilization [7]. They are also not annotated as 'transposable-element genes' in TAIR10. To highlight the potential links to TEs, we separated these genes in Figure 2d. Thank you for this suggestion!*

#6: Possible typo?: Col-o / Col-0 (e.g page 26 line 4).

*Response: Sorry, this seems to have been an issue with PDF conversion.*

*References:*

*1. Baduel P, Leduque B, Ignace A, Gy I, Gil J Jr, Loudet O, et al. Genetic and environmental modulation of transposition shapes the evolutionary potential of Arabidopsis thaliana. Genome Biol. 2021;22:138.*

*2. Baduel P, Quadrana L, Colot V. Efficient Detection of Transposable Element Insertion Polymorphisms Between Genomes Using Short-Read Sequencing Data. In: Cho J, editor. Plant Transposable Elements: Methods and Protocols. New York, NY: Springer US; 2021. p. 157–69.*

*3. Mirouze M, Reinders J, Bucher E, Nishimura T, Schneeberger K, Ossowski S, et al. Selective epigenetic control of retrotransposition in Arabidopsis. Nature. 2009;461:427–30.*

*4. Yi H, Richards EJ. Gene duplication and hypermutation of the pathogen Resistance gene SNC1 in the Arabidopsis bal variant. Genetics. 2009;183:1227–34.*

*5. Stokes TL, Kunkel BN, Richards EJ. Epigenetic variation in Arabidopsis disease resistance. Genes Dev. 2002;16:171–82.*

*6. Lei M, Zhang H, Julian R, Tang K, Xie S, Zhu J-K. Regulatory link between DNA methylation and active demethylation in Arabidopsis. Proc Natl Acad Sci U S A. 2015;112:3553–7.*

*7. van Leeuwen H, Monfort A, Puigdomenech P. Mutator-like elements identified in melon, Arabidopsis and rice contain ULP1 protease domains. Mol Genet Genomics. 2007;277:357–64.*

*Apart from the revisions highlighted above, we made the following changes during the revision:*

*1) Genome browser screenshots have been updated to show the same colors for methylation in the forward and reverse strands (Figures S25, S26, S27, S28).*

*2) Figure S22 has been updated to represent intersections between HV-dACRs and consensus DEGs.*

*3) Panels (a) and (c) in Figure S25 have been updated based on the intersections between HV-dACRs, consensus DEGs and DMRs.*

*4) Figure S13 has been updated to represent mean accessibility at HV-dACRs, and distance of HV-dACRs to TEs and Non-TE genes.*

*5) Metaplots of accessibility levels across TEs, genes and DEGs in Figure S14 have been updated. These were previously generated using dACR accessibility values for each sample, represented across the entire length of every accessible region. We have now identified peak summits specific to each sample and peak region, and generated the same metaplots (retaining the accessibility value for every peak) by representing every peak region as 100 bp upstream and downstream of the peak summit. The trend of increased accessibility across TEs and DEGs in met1 mutants remains unchanged.*

**Second round of review**

**Reviewer 1**

In my initial review of this manuscript, I found the paper to be a strong contribution to the field but had concerns about two important aspects. First, I was concerned that comparing the number of DEGs was not the most accurate way to quantitatively assess expression variability, and second, I felt the analysis of ATAC-seq data could be improved with greater stringency.

I am delighted to see that my suggestions have been dispatched with aplomb. The revisions are all of excellent quality and bolster an already-strong manuscript. I do think that the violin plots of expression CV are more informative than assessments of #DEGs, particularly as the title and abstract of the paper lean heavily on the role of CG methylation in canalizing expression. My personal view is that the violin plots of CV provide a strong, simple and quantitative demonstration of this main conclusion of the paper, capturing the extent of canalization in WT vs met1 in a single image. I'd recommend ideally finding a way to squeeze one of the violins into main Figures 1 or 2, or at the very least keeping the supplemental figure as it is currently.

I also think the ATAC-seq analysis is much improved and the associations between chromatin accessibility and methylation are much clearer.

-- Ben Williams

**Reviewer 2**

It was a constructive review. Looking forward to the published manuscript.

**Authors Response**

**Point-by-point responses to the reviewers’ comments:**

Reviewer #1:

In my initial review of this manuscript, I found the paper to be a strong contribution to the field but had concerns about two important aspects. First, I was concerned that comparing the number of DEGs was not the most accurate way to quantitatively assess expression variability, and second, I felt the analysis of ATAC-seq data could be improved with greater stringency.

I am delighted to see that my suggestions have been dispatched with aplomb. The revisions are all of excellent quality and bolster an already-strong manuscript. I do think that the violin plots of expression CV are more informative than assessments of #DEGs, particularly as the title and abstract of the paper lean heavily on the role of CG methylation in canalizing expression. My personal view is that the violin plots of CV provide a strong, simple and quantitative demonstration of this main conclusion of the paper, capturing the extent of canalization in WT vs met1 in a single image. I'd recommend ideally finding a way to squeeze one of the violins into main Figures 1 or 2, or at the very least keeping the supplemental figure as it is currently.

I also think the ATAC-seq analysis is much improved and the associations between chromatin accessibility and methylation are much clearer.

-- Ben Williams

Reviewer #2:

It was a constructive review. Looking forward to the published manuscript.

*Response: We thank the reviewers for their feedback on our revisions. As suggested by Reviewer 1, we have now incorporated the violin plots of expression CV into the main Figure 1.*
